# Survival and quality of life after first-time diagnosis of brain metastases: a multicenter, prospective, observational study

**DOI:** 10.1016/j.lanepe.2024.101181

**Published:** 2024-12-19

**Authors:** Olav Erich Yri, Guro Lindviksmoen Astrup, Astrid Telhaug Karlsson, Rene van Helvoirt, Marianne Jensen Hjermstad, Kristin Moksnes Husby, Jon Håvard Loge, Jo-Åsmund Lund, Tonje Lundeby, Ørnulf Paulsen, Eva Skovlund, Marius-Ioan Taran, Rebecca Rootwelt Winther, Nina Aass, Stein Kaasa

**Affiliations:** a Department of Oncology, Oslo University Hospital, PO Box 4950 Nydalen, Oslo, 0424, Norway; b European Palliative Care Research Centre (PRC), Institute for Clinical Medicine, PO Box 1171, Blindern, Oslo, 0318, Norway; c Department of Oncology, Sorlandet Hospital Trust, PO Box 416 Lundsiden, Kristiansand, 4604, Norway; d Department of Surgery, Vestre Viken Hospital Trust, PO Box 800, Drammen, 3004, Norway; e Clinic for Cancer Treatment and Rehabilitation, Møre and Romsdal Hospital Trust, PO Box 1600, Ålesund, 6026, Norway; f Department of Oncology and Hematology, Telemark Hospital Trust, PO Box 2900 Kjørbekk, Skien, 3710, Norway; g Department of Public Health and Nursing, Faculty of Medicine and Health Sciences, Norwegian University of Science and Technology (NTNU), PO Box 8905, Trondheim, 7491, Norway; h Department of Oncology and Hematology, Vestfold Hospital Trust, PO Box 2168, Tønsberg, 3103, Norway; i Department of Health Sciences, Faculty of Medicine and Health Services, Norwegian University of Science and Technology, PO Box 1517, Ålesund, 6025, Norway

**Keywords:** Brain metastases, Anticancer treatment, Treatment decisions, Radiotherapy, Futile treatment

## Abstract

**Background:**

A major concern in anticancer treatment (ACT) of brain metastases (BM) is exposing patients with short expected survival to treatments that negatively impact on quality of life (QoL). Such futile ACT at the end of life is time-consuming and burdensome for patients and their families and entails unnecessary healthcare costs. Refraining from ACT is challenging for both physicians and patients. This study aimed to provide real-life data on survival after BM diagnosis and patient reported outcomes (PROs) after ACT to identify risk factors for futile treatment and to support BM treatment decisions.

**Methods:**

This multi-center, prospective, observational study recruited consecutive patients with first-time BM from November 2017 to March 2021. Patients were followed until death or study end (October 1st, 2023). Clinical factors associated with survival were analyzed by the Cox’ proportional hazards model. Changes in PROs after BM treatment were described according to Eastern Cooperative Oncology Group (ECOG) performance status, survival, and treatment groups.

**Findings:**

For the total cohort (*N* = 912), median overall survival (mOS) after BM diagnosis was 5.9 months (95% confidence interval [CI] 5.2–6.7). ECOG 2–4, uncontrolled extracranial metastases, and ≥5 BM were associated with short survival. In patients treated with radiotherapy, survival for patients with ECOG 2 and those with ECOG 3–4 was similar and particularly short for the whole brain radiotherapy (WBRT) group (ECOG 2: 2.9 months [95% CI 2.3–3.5]; ECOG 3–4: 2.1 [1.5–2.7]). Patients surviving <6 months after BM diagnosis reported worse QoL scores two months after ACT; patients surviving >6 months reported stable scores over time.

**Interpretation:**

Patients with ECOG 2–4, especially those with uncontrolled extracranial metastases and ≥5 BM, are at risk for futile ACT. BM treatment guidelines should strongly caution against ACT to patients with expected survival <6 months and specifically advise against WBRT.

**Funding:**

The 10.13039/501100006095South-Eastern Norway Regional Health Authority; The 10.13039/100008730Norwegian Cancer Society.

**Research in context**

**Evidence before this study**

We searched PubMed until March 1, 2017 for randomized clinical trials (RCTs) and prospective cohort studies regarding brain metastases (BM) treatment and quality of life after treatment. Terms used were related to “brain metastases treatment” (*i.e.,* “brain neoplasms”, “surgery”, “radiotherapy”, “treatment(s)”, “quality of life”, and “patient-reported outcomes”), published in English language. Several retrospective studies, prospective observational studies, and randomized clinical trials (RCTs) were retrieved. Before 1990, most RCTs concerned different whole brain radiotherapy (WBRT) regimes, with or without potential effect-enhancing drugs. After 1990, more RCTs increasingly focused on combinations of surgery, stereotactic or whole brain radiotherapy and/or novel systemic drugs, with overall survival and/or intracranial control as outcomes. In RCTs, Eastern Cooperative Oncology Group (ECOG) 0–2 and/or Karnofsky performance scale (KPS) ≥ 70 and 1–4 BM were the most common inclusion criteria; only one larger trial focused on patients with short expected survival. The two identified BM treatment guidelines were unclear regarding which groups of patients that are unlikely to benefit from anticancer treatment. Although palliative care alone was acknowledged for patients with short expected survival, radiotherapy was kept as an option despite low level of evidence (class 3 or B). The definitions of “good” and “poor” performance status (PS) were also not clear-cut. Most of the prospective studies that included patient reported outcomes (PROs) had low patients numbers; only four identified studies included more than 200 patients. The studies suggested deterioration in several aspects of quality of life (QoL) for patients with short survival after RT, but attrition rates in follow-up QoL data were high as many patients died within three months after anticancer treatment, especially after WBRT.

**Added value of this study**

This is, to our knowledge, the largest prospective, observational cohort study on BM treatment effects including PROs for up to one year after inclusion. It confirms the dismal survival after the BM diagnosis in a high proportion of patients and demonstrates a decline in central QoL domains such as global QoL, physical function, and fatigue in patients with <6 month survival and/or ECOG ≥ 2, especially after RT. The study also identifies ECOG 2 as a significant risk factor for short survival for patients with BM and confirms and strengthens the observations from a very restricted number of RCTs of minimal benefit after RT in patients with short expected survival.

**Implications of all the available evidence**

In order to prevent futile anticancer treatment, caution is warranted against anticancer treatment for patients with BM and an estimated survival <6 months.

## Introduction

Anticancer treatment (ACT) in patients with brain metastases (BM) aims at achieving intracranial tumor control to prolong survival and improve symptoms, functioning, and quality of life (QoL). However, as survival may be short for many patients following a BM diagnosis, exposing those with the poorest prognosis to ACT carries the risk of inflicting burdensome side-effects towards the end of life without achieving meaningful benefits. It is therefore paramount to identify groups of patients with BM that are unlikely to benefit from ACT.

BM treatment options include surgery, radiotherapy (RT), systemic treatments, and best supportive care (BSC), alone or in combinations. International guidelines^1^, ^2^, ^3^, ^4^, ^5^ describe treatment indications guided by estimated survival, patient-related factors (*i.e.,* performance status [PS], age) and indicators of tumor burden (*i.e.,* number of BM, status of extracranial disease).^6^, ^7^, ^8^ These factors are also reflected in prognostic scoring systems such as the recursive partitioning analysis (RPA) classification^6^ and the diagnosis-specific graded prognostic assessment (DS-GPA).^8^ Briefly, indications for surgery are lesions accessible for resection, rapid relief of raised intracranial pressure, and/or need for biopsy. Post-operative RT is recommended, preferably as stereotactic RT (SRT). SRT is an alternative for patients with 1–4 BM < 4 cm and good PS (Eastern Cooperative Oncology Group [ECOG] 0–2 and/or Karnofsky performance scale [KPS] ≥ 70) with whole brain RT (WBRT) mainly reserved for patients with multiple (≥5) BM not candidates for surgery or SRT. Systemic treatments with intracranial effects are available as first-line BM treatment, especially for asymptomatic patients with targetable mutations and/or in need of concomitant treatment of extracranial disease.^1^, ^2^, ^3^, ^4^, ^5^

The guidelines are less precise regarding when to refrain from ACT. Although BSC alone is recommended for patients with an expected survival of less than three months, poor PS and/or limited systemic therapeutic options,^1^, ^2^, ^3^, ^4^, ^5^ the updated ASTRO and EANO-ESMO guidelines still include RT as an alternative for these patients.^3^^,^^5^ Subsequently, RT is frequently offered to patients with a short life expectancy despite limited scientific evidence from clinical trials.^9^^,^^10^

Patient reported outcomes (PROs) provide direct information of patient's own evaluation of symptoms and QoL. Such evaluations are important elements to consider in treatment decisions and patient follow-up.^11^ PROs can be collected prospectively in observational studies, randomized clinical trials (RCTs), and in routine clinical care, although the latter is rarely systematically done. The primary outcomes applied in RCTs on BM treatments are mainly survival or intracranial control^12^, ^13^, ^14^, ^15^, ^16^, ^17^ with PROs being secondary outcomes,^12^, ^13^, ^14^, ^15^, ^16^, ^17^, ^18^, ^19^, ^20^, ^21^ with some exceptions.^10^^,^^22^ As RCTs commonly exclude patients with ECOG 3–4/KPS < 70 or >4 BM, the transferability of results from RCTs to real-life populations may be limited. Prospective studies investigating PROs are heterogeneous in cohort compositions, sizes, designs and methods, often with high attrition rates.^23^, ^24^, ^25^, ^26^, ^27^, ^28^, ^29^, ^30^ Taken together, larger, unselected patient cohort studies focusing on associations between BM treatment, survival and PROs are scarce. To provide optimal patient-centered care and facilitate true shared decision-making,^11^ more knowledge about the associations between the benefits and burdens of ACT is needed.

To this end, a prospective, observational Norwegian study including consecutive patients diagnosed with first-time BM was initiated to obtain real-life data on patient- and disease-related factors, initial BM treatment, and PROs to inform treatment decisions. The present article reports overall survival and PROs over time in the total patient cohort.

The following research questions are addressed: How does provision of treatment adhere to guidelines applicable during the study period? What is the survival after first-time BM diagnosis? Which factors are associated with survival and which are informative for tumor-directed treatment decisions? How do PROs and QoL develop after BM treatment?

## Methods

This prospective, observational study included consecutive patients diagnosed with first-time BM from November 2017 through March 2021 at five hospitals in the Central- and South-East health regions of Norway. Three of these provide RT; two are referral centers for RT in their regions. Diagnostics, treatment decisions, and follow-up were per routine at each participating center.

### Participants

Patients were identified through systematic screening of radiology reports, referrals for BM treatment, or patients scheduled to start BM treatment. Eligible patients were ≥18 years with first-time BM from solid cancers verified radiologically and/or by biopsy, and able to provide written consent in Norwegian. Exclusion criteria were primary brain tumors, hematological malignancies, or relapse/progression of previous BM. Patients were included at start of initial BM treatment or within two weeks after BM resection.

### Treatments

Treatments included surgery, SRT, WBRT, systemic treatments, and BSC, either alone or in combination. Radiotherapy was conducted according to local treatment protocols. SRT was given in 1–5 fractions with 3–25 gray (Gy) per fraction (one patient received 4 Gy × 9 to a larger, single BM). WBRT was given as two opposing fields or VMAT (20 Gy in five fractions or 30 Gy in 10 fractions). RT was given with linear accelerators (LINACs); gamma knife was not used.

### Data collection

Demographic and clinical data related to treatment and status of the primary tumor and intra- and extracranial metastases (ECM) were recorded at inclusion and every three months up to 24 months after inclusion or death. Complete data list and definitions are summarized in Supplementary Table S1. Follow-up data were accrued from the medical records from regular patient hospital visits closest to the three-monthly date. Data not registered in the medical records were recorded as “missing”. Radiological and pathological data were extracted from routine reports, with date of BM diagnosis and number of BM derived from the first radiology report explicitly describing BM (CT scans only if MRI was not performed). Date of primary cancer was retrieved from the Norwegian Cancer Registry. For patients with a history of more than one primary cancer, the one most likely to be the cause of BM was defined as primary cancer. Controlled ECM was defined as no evidence of or stable ECM at radiology evaluation closest in time to BM diagnosis. Uncontrolled ECM was defined as progressive ECM, diagnosed concomitantly with BM, or unknown status.

### Prognostic scores

DS-GPA was calculated as described by Sperduto et al.^8^ ECOG status was converted to KPS as follows: ECOG 0: KPS 90–100, ECOG 1: KPS 80, ECOG 2: KPS 60–70, ECOG 3–4: KPS 50–10.^31^ RPA was calculated according to Gaspar et al.,^6^ with ECOG 0–2 converted to KPS ≥ 70, ECOG 3–4 to KPS < 70. A simplified model using ECOG (0–1 vs. 2–4), status of ECM (controlled vs. uncontrolled), and number of BM (1–4 vs. ≥5) was compared to the DS-GPA calculations.

### Patient-reported outcomes

PROs were collected using the European Organisation for Research and Treatment of Cancer (EORTC) questionnaire for palliative care (EORTC QLQ-C15-PAL)^32^ and the diagnosis-specific module EORTC QLQ-BN20^33^ at study inclusion, then monthly by postal mail for up to 12 months thereafter. If not returned, only one reminder was sent. Questionnaire details (validity, content, scoring, and clinically significant change) are provided in the Supplementary Material.

### Statistical analysis

Overall survival was estimated from date of the BM diagnosis to death from any cause using the Kaplan–Meier method and compared between groups by the log rank test. Patients alive were censored at the last date of survival follow-up (Oct. 1st, 2023). Multivariable analyses were performed by Cox’ proportional hazards model. All clinical variables registered at baseline were included in the model. Multivariable analyses were performed for the total patient cohort and repeated separately for each treatment group. The proportional hazards assumption was checked by visual inspection of log-minus-log plots.

Mean PRO scores at time of inclusion (baseline) and at month 2 after inclusion are presented according to ECOG PS at inclusion, survival after date of BM diagnosis, and treatments. For patients with complete PROs at both time points the mean difference between scores at baseline and month 2 with a 95% confidence interval (95% CI) is presented. Missing data in the EORTC questionnaires were handled according to the EORTC scoring manual, in which procedures for handling missing item responses is described (see Supplementary Material for details). Clinically meaningful changes (defined as ≥10 points change)^34^ are presented. All domains from the EORTC QLQ-C15-PAL and BN20 questionnaires were analyzed, with overall QoL, physical functioning (PF), and fatigue as the primary outcomes based on previous literature and clinical experience.

SPSS Statistics for Windows, version 29 (IBM Corp., Armonk, N.Y., USA) and STATA version 18 (Metrika Consulting AB, Stockholm, Sweden) were used.

### Ethical considerations

All patients provided written informed consent. The study was approved by The Regional Committee for Medical and Health Research Ethics in South-Eastern and Middle Norway health care regions (REK no. 2017/1358) and the data protection officer at each hospital. All procedures were in accordance with the 1975 Helsinki declaration and later amendments. This study is registered with ClinicalTrials.gov (NCT03346655).

### Role of the funding sources

The funders of the study had no role in study design, data collection, data analysis, data interpretation, or writing of the report.

## Results

### Patient characteristics

Of 1406 patients screened, 980 were included and 912 were eligible for analyses (Supplementary Figure S1). Median age was 69 (21–96), 54% were female. Non-small cell lung cancer (44%), melanoma (16%), and breast cancer (14%) were most frequent (Table 1, Supplementary Table S2).

**Table 1:** Baseline patient demographics and clinical characteristics for the total patient cohort and for each separate treatment group.

	All patients	Treatment groups				
		Surgery	SRT	WBRT^a^	Systemic	BSC
	*N* (%)	*N* (%)	*N* (%)	*N* (%)	*N* (%)	*N* (%)
	912 (100)	155 (17)	306 (34)	370 (41)	31 (3)	50 (6)
Sex						
Male	419 (46)	65 (42)	156 (51)	161 (44)	16 (52)	21 (42)
Female	493 (54)	90 (58)	150 (49)	209 (57)	15 (48)	29 (58)
Age (median; min-max)	(69; 21–96)	(66; 21–85)	(70; 27–96)	(69; 27–92)	(66; 28–82)	(74; 41–88)
<50	78 (9)	18 (12)	23 (8)	30 (8)	6 (19)	1 (2)
50–59	130 (14)	30 (19)	38 (12)	53 (14)	6 (19)	3 (6)
60–69	262 (29)	46 (30)	88 (29)	106 (29)	8 (26)	14 (28)
70–79	346 (38)	54 (35)	123 (40)	139 (38)	10 (32)	20 (40)
≥80	96 (11)	7 (5)	34 (11)	42 (11)	1 (3)	12 (24)
ECOG perfomance status						
0–1	519 (57)	105 (68)	205 (67)	181 (49)	20 (65)	8 (16)
2	214 (24)	34 (22)	64 (21)	104 (28)	4 (13)	8 (16)
3–4	163 (18)	14 (9)	33 (11)	77 (21)	6 (19)	33 (66)
Missing	16 (2)	2 (1)	4 (1)	8 (2)	1 (3)	1 (2)
Primary cancer						
Breast	129 (14)	25 (16)	30 (10)	68 (18)	2 (7)	4 (8)
Lung	406 (45)	52 (34)	155 (51)	161 (44)	14 (45)	24 (48)
Melanoma	148 (16)	30 (19)	52 (17)	48 (13)	10 (32)	8 (16)
Colorectal	86 (9)	23 (15)	29 (10)	30 (8)	1 (3)	3 (6)
Kidney	32 (4)	4 (3)	7 (2)	17 (5)	1 (3)	3 (6)
Other^b^	111 (12)	21 (14)	33 (11)	46 (12)	3 (10)	8 (16)
Number of BM						
1	324 (36)	108 (70)	166 (54)	28 (8)	7 (23)	15 (30)
2	134 (15)	21 (14)	73 (24)	31 (8)	4 (13)	5 (10)
3–4	133 (15)	16 (10)	61 (20)	40 (11)	6 (19)	10 (22)
≥5, incl. leptomeningeal	316 (35)	10 (7)	6 (2)	268 (72)	13 (42)	19 (38)
Missing	5 (1)	0 (0)	0 (0)	3 (1)	1 (3)	1 (2)
Symptomatic						
No	161 (18)	9 (6)	76 (25)	45 (12)	18 (58)	13 (26)
Yes	751 (82)	146 (94)	230 (75)	325 (88)	13 (42)	37 (74)
ECM absent/present						
Absent	186 (20)	67 (43)	66 (22)	46 (12)	1 (3)	6 (12)
Present	726 (80)	88 (57)	240 (78)	324 (88)	30 (97)	44 (88)
ECM status						
None or stable	391 (44)	109 (70)	142 (46)	123 (33)	4 (13)	13 (26)
At diagnosis	279 (31)	34 (22)	92 (30)	116 (31)	18 (58)	19 (38)
Progression	218 (24)	12 (8)	62 (20)	121 (33)	8 (26)	15 (30)
Not evaluated/missing	24 (3)	0 (0)	10 (3)	10 (3)	1 (3)	3 (6)
Steroids at inclusion^c^						
No or at SRT only	186 (20)	47 (30)	93 (30)	22 (6)	15 (48)	9 (18)
Yes	725 (80)	108 (70)	213 (70)	348 (94)	15 (48)	41 (82)
Missing	1 (1)	0 (0)	0 (0)	0 (0)	1 (3)	0 (0)
Targetable mutations^d^						
No	463 (51)	78 (50)	139 (45)	197 (53)	19 (61)	30 (60)
Yes	449 (49)	77 (50)	167 (55)	173 (47)	12 (39)	20 (40)

Number and proportion (%) of patients having the characteristic. SRT: Stereotactic radiotherapy. WBRT: Wholde brain radiotherapy. BSC: Best supportive care. ECM: Extracranial metastases.

a WBRT includes partial brain (*N* = 4).

b See Supplementary Table S2 for detailed specification.

c Use of steroids at time of inclusion.

d Mutation or positivity for at least one of the following: ALK, EGFR, BRAF, HER-2, KRAS, MSI, NRAS, PD-L1, Progesterone, ROS1, Estrogen.

### Initial BM treatment

Patients were grouped according to initial BM treatment: Surgery (*N* = 155/17%), SRT (*N* = 306/34%), WBRT (*N* = 370/41%), systemic (*N* = 31/3%), and BSC (*N* = 50/5%; Table 1, Supplementary Figure S2). Post-operative RT to the resection cavity was given to 121/155 (78%) patients (SRT 66%, WBRT 24%, partial brain 10%). Of these, 24% received RT within 30 days after surgery (86% within 60; 97% within 90 days). Patient- and clinical characteristics for the total cohort and each treatment group are presented in Table 1.

### Survival

**Fig. 1:**
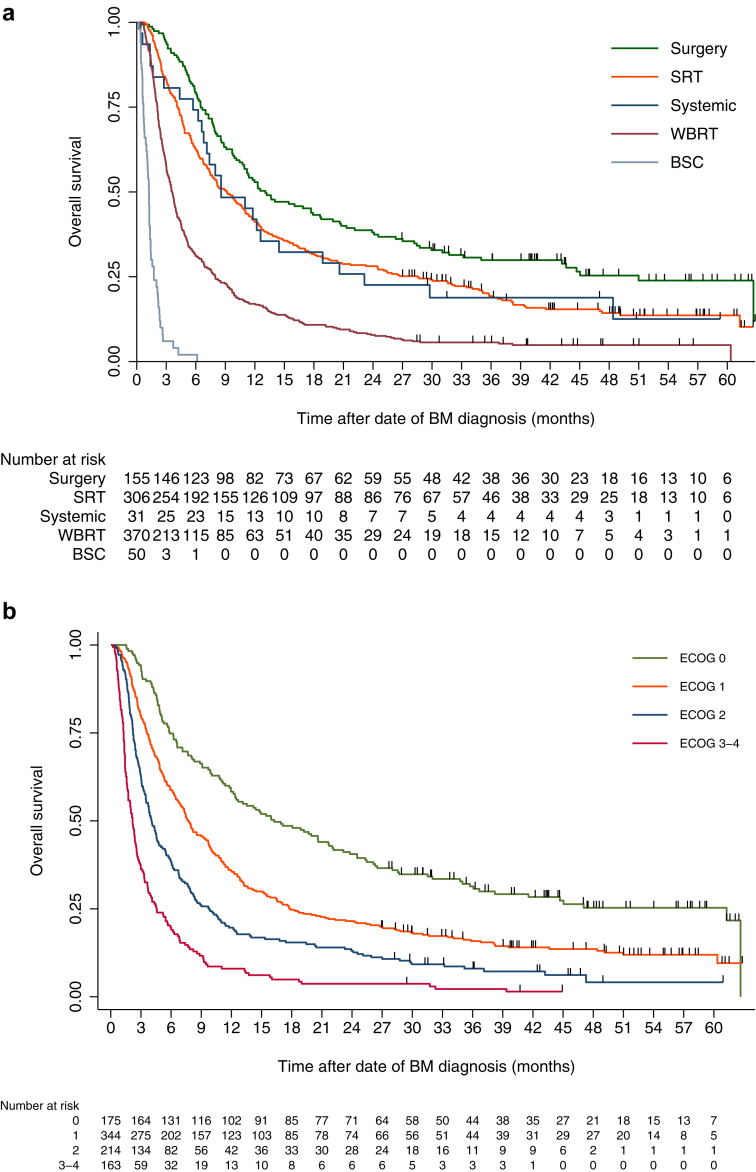

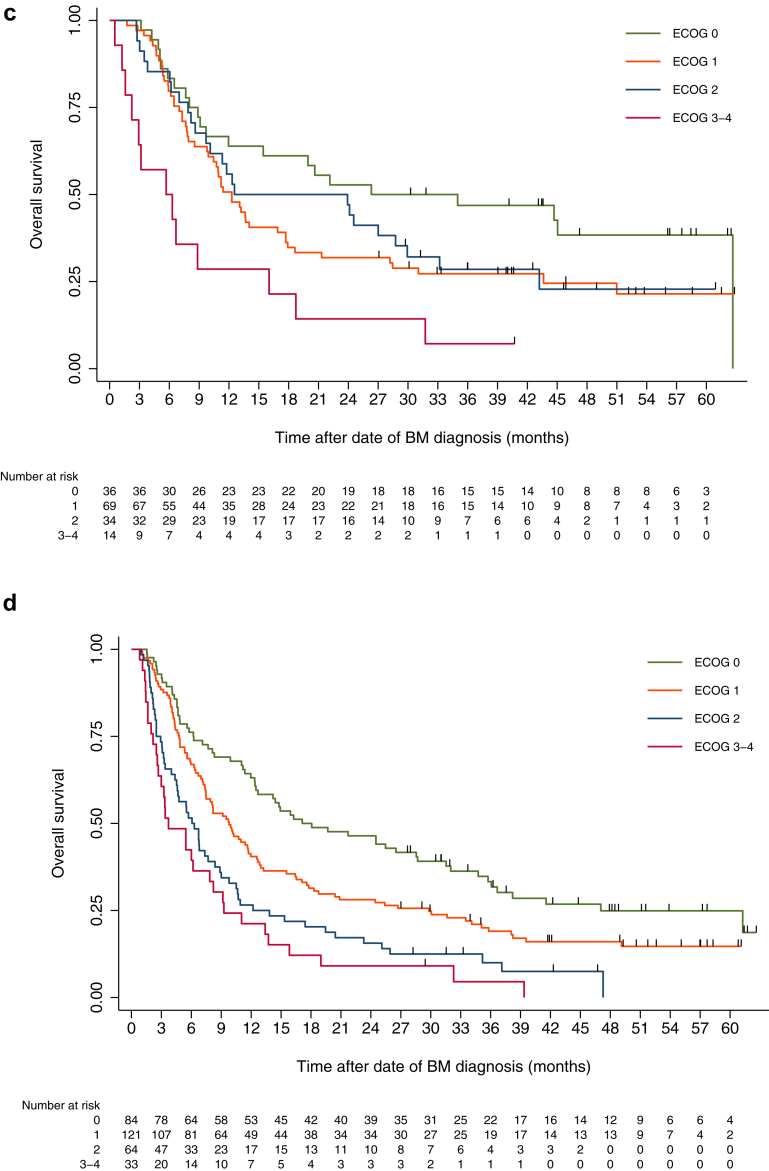

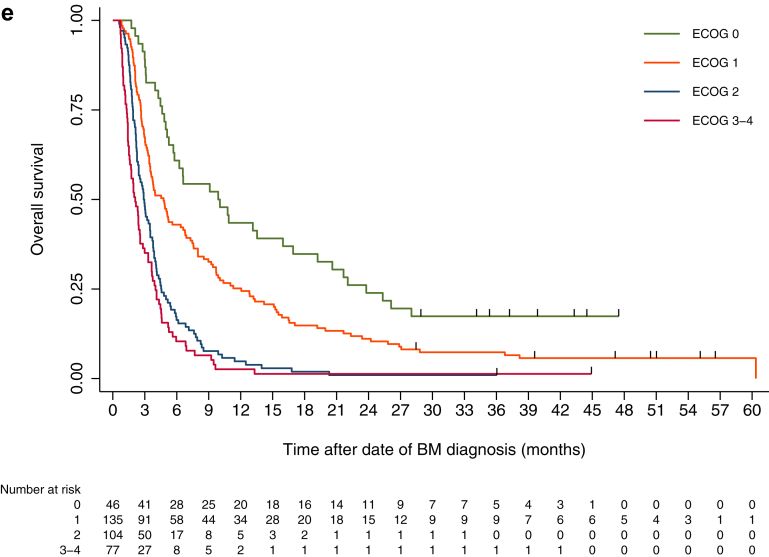
**Kaplan–Meier curves for survival (months) stratified by treatment groups and ECOG-status. Median overall survival (mOS) and 95% confidence intervals (95% CI)**. a) Survival by treatment groups. Overall: 5.9 (95% CI 5.2–6.7), Surgery: 13.1 (8.7–17.5), SRT: 9.2 (7.3–11.0), WBRT: 3.6 (3.2–4.0), Systemic: 8.6 (3.8–13.4), BSC: 1.2 (1.1–1.4). b) Survival by ECOG, total patient cohort. Overall: 5.9 (5.2–6.7), ECOG 0: 16.2 (10.9–21.7), ECOG 1: 7.7 (6.3–9.0), ECOG 2: 4.0 (3.4–4.7), ECOG 3–4: 2.2 (1.8–2.5). c) Survival by ECOG, Surgery group. Overall: 13.1 (8.7–17.5), ECOG 0: 26.3 (0.0–56.7), ECOG 1: 12.3 (9.7–14.9), ECOG 2: 12.6 (0.0–30.2), ECOG 3–4: 5.7 (0.0–11.5). d) Survival by ECOG, SRT group. Overall: 9.3 (7.5–11.0), ECOG 0: 17.1 (8.1–26.2), ECOG 1: 9.8 (7.4–12.2), ECOG 2: 6.1 (4.1–8.0), ECOG 3–4: 3.7 (1.2–6.2). e) Survival by ECOG, WBRT group. Overall: 3.6 (3.2–4.0), ECOG 0: 9.9 (5.1–14.7), ECOG 1: 4.8 (3.6–5.9), ECOG 2: 2.9 (2.3–3.5), ECOG 3–4: 2.1 (1.5–2.7). Survival calculated by Kaplan–Meier analyses. mOS: Median overall survival. CI: Confidence interval. SRT: Stereotactic radiotherapy. WBRT: Whole brain radiotherapy. BSC: Best supportive care. ECOG: Eastern Cooperative Oncology Group performance status.

At the last update (October 1st, 2023, minimum follow-up 30 months), 799 patients (88%) had died. mOS after the date of BM diagnosis was 5.9 months (95% CI 5.2–6.7) for the total cohort (surgery group 13.1 months (8.7–17.5); SRT 9.2 months (7.3–11.0); WBRT 3.6 months (3.2–4.0); systemic 8.6 months (3.8–13.4); BSC 1.2 months (1.1–1.4), Fig. 1a).

For the total cohort, multivariable analyses showed significant associations with poorer survival for ECOG ≥ 2, age ≥70, male sex, >1 BM, uncontrolled ECM, colorectal cancer, and absence of targetable mutations (Table 2), with the highest HR for ECOG ≥ 2, uncontrolled ECM, ≥5 BM, and colorectal cancer. In the surgery group, significant associations were found for ECOG 3–4, uncontrolled ECM, colorectal cancer and no targetable mutations, and for ECOG 2–4 and uncontrolled ECM in both RT groups. Additionally, age ≥70 and colorectal cancer were associated with poorer survival for the SRT and male sex for the WBRT group (Supplementary Table S3). Small patient numbers precluded multivariable analyses in the systemic and BSC groups.

**Table 2:** Hazard ratios for overall survival in the total patient cohort.

	All patients (*N* = 912)				
	*N*	Cox proportional hazards model			
		Univariable		Multivariable (*N* = 892)	
		HR (95% CI)	p	HR (95% CI)	p
Age					
<70	470	1		1	
≥70	442	1.52 (1.32–1.75)	<0.001	1.37 (1.18–1.60)	<0.001
Sex					
Male	419	1		1	
Female	493	0.84 (0.73–0.96)	0.01	0.83 (0.71–0.97)	0.02
ECOG performance status					
0–1	519	1		1	
2	214	1.85 (1.56–2.20)	<0.001	1.85 (1.55–2.20)	<0.001
3–4	163	3.54 (2.93–4.26)	<0.001	2.95 (2.43–3.58)	<0.001
Missing	16				
Primary cancer					
Breast	129	1		1	
Lung	406	1.45 (1.16–1.80)	<0.001	1.04 (0.81–1.32)	0.77
Colorectal	86	2.20 (1.65–2.95)	<0.001	1.59 (1.15–2.20)	0.01
Melanoma	148	1.28 (0.99–1.67)	0.06	0.81 (0.61–1.09)	0.16
Kidney	32	1.50 (1.00–2.04)	0.05	0.93 (0.59–1.45)	0.74
Others	111	1.55 (1.18–2.04)	0.01	1.09 (0.80–1.49)	0.58
Number of BM					
1	324	1		1	
2–4	267	1.39 (1.17–1.66)	<0.001	1.40 (1.17–1.68)	<0.001
≥5 (incl lepto)	316	1.94 (1.64–2.29)	<0.001	1.99 (1.66–2.38)	<0.001
Missing	5				
Symptomatic					
No	161	1		1	
Yes	751	1.20 (0.99–1.44)	0.06	1.05 (0.86–1.27)	0.64
ECM status					
Controlled	391	1		1	
Uncontrolled	521	1.76 (1.53–2.03)	<0.001	1.55 (1.33–1.79)	<0.001
Targetable mutations^a^					
Yes	448	1		1	
No	464	1.46 (1.27–1.68)	<0.001	1.30 (1.11–1.52)	0.001

Estimates from Cox proportional hazards models. The multivariable model comprises all clinical variables included in the unadjusted models. HR: Hazard ratio. CI: Confidence interval. ECM: Extracranial metastases.

a Mutation or positivity for at least one of the following: ALK, EGFR, BRAF, HER-2, KRAS, MSI, NRAS, PD-L1, Progesterone, ROS1, Estrogen.

### Survival according to ECOG status

For the surgery group, ECOG 3–4 was associated with shorter survival compared to both ECOG 0–1 and ECOG 2. For the SRT and WBRT groups, both ECOG 2 and 3–4 were associated with shorter survival compared to ECOG 0–1. For both RT groups, median survival was similar and the survival curves for ECOG 2 and ECOG 3–4 were almost overlapping (SRT group: ECOG 2 mOS 6.1 months (4.1–8.0), ECOG 3–4 3.7 months (1.2–6.2); WBRT group: ECOG 2 2.9 months (2.3–3.5), ECOG 3–4 2.1 months (1.5–2.7) [Fig. 1b–e, Supplementary Table S4]).

### Prognostic scores

DS-GPA and RPA calculations provided significant associations between DS-GPA classes and RPA classes and survival (p < 0.001). In the simplified model, significant associations with survival was also found for the calculated subgroups (p < 0.001, Supplementary Table S5).

### Patient reported outcomes (PROs)

Overall, 740 (81%) patients completed at least one PRO-assessment (Supplementary Table S6). Attrition was high (Supplementary Table S7). For responders who died during the 12 months of PROs assessment, median time from last response to death was 5.0 weeks. Compared to responders, non-responders were more likely to be older (mean age 69 vs. 66), have ECOG 3–4 and to have shorter survival (mean mOS 5.5 vs. 11.5, data not shown).

### PROs over time by ECOG status at inclusion

**Fig. 2:**
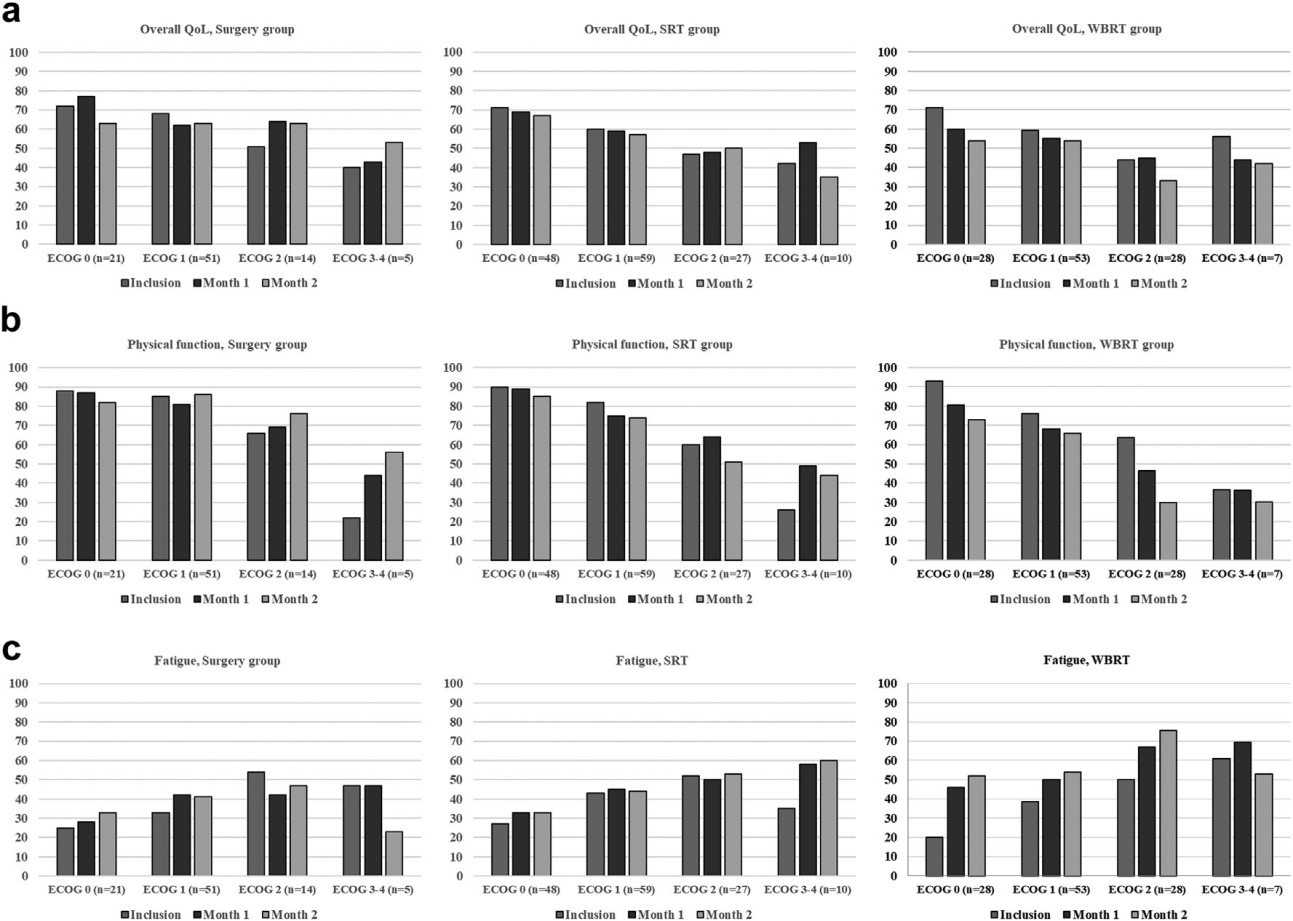
**Patient reported outcome scores (mean) at inclusion, month 1 and month 2 stratified by treatment group and ECOG-status**. Only patients responding at all three time points in each ECOG group were included in the analyses. a) Overall quality of life (QoL). b) Physical function. c) Fatigue. Higher scores for overall QoL and PF indicate better function. Higher scores for fatigue indicate worse symptoms. QoL: Quality of life. SRT: Stereotactic radiotherapy. WBRT: Whole brain radiotherapy. ECOG: Eastern Cooperative Oncology Group performance status.

At month 2, compared to time of inclusion into the study, PRO scores remained stable or improved for overall QoL, physical functioning (PF), and fatigue for the surgery group (*N* = 72), regardless of ECOG status (Fig. 2, Supplementary Table S8). For SRT patients (*N* = 121), mean overall QoL scores remained stable across ECOG groups, whereas for the ECOG 3–4 group, PF improved and fatigue worsened (Fig. 2, Supplementary Table S9). In contrast, for the WBRT group (*N* = 101), mean scores were either stable or worse for these domains regardless of ECOG status (Fig. 2, Supplementary Table S10). Attrition precluded comparisons over time in the systemic and BSC groups.

### PROs over time by survival after BM diagnosis

**Fig. 3:**
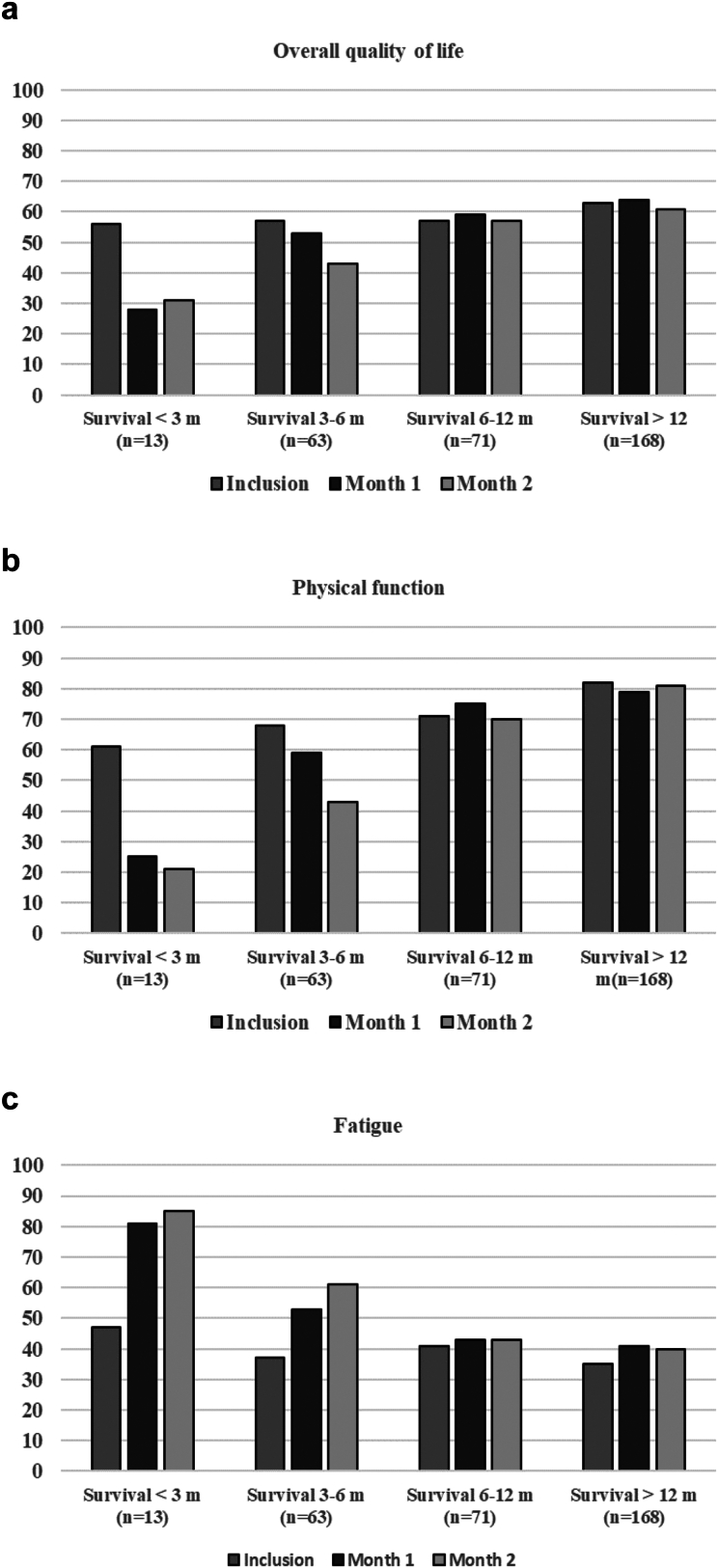
**Patient reported outcome scores (mean) at inclusion, month 1 and month 2 stratified by survival**. Only patients responding at all three time points in each group were included in the analyses. a) Overall quality of life (QoL). b) Physical function. c) Fatigue. Higher scores for overall QoL and PF indicate better function. Higher scores for fatigue indicate worse symptoms. QoL: Quality of life. SRT: Stereotactic radiotherapy. WBRT: Whole brain radiotherapy.

PRO scores analyzed by subgroups according to survival after BM diagnosis (<3 months, 3–6 months, 6–12 months, >12 months) showed that at month 2, patients in the <3 months (*N* = 13) and 3–6 months (*N* = 63) survival groups reported worse mean scores for overall QoL, PF, fatigue, and most other domains. For patients in the 6–12 month (*N* = 71) and >12 month (*N* = 168) groups, mean scores remained stable for overall QoL and PF; for fatigue, patients in the >12 month group reported worse scores (Fig. 3, Supplementary Table S11). When analyzing patients with non-lung cancer and lung cancer separately, an identical pattern of change in mean scores for overall QoL, PF, and fatigue was found. In both groups, patients with survival less than six months had worse scores in all three domains at month 2 (Supplementary Tables S12 and S13).

### PROs over time by treatment groups

At month 2, compared to inclusion, scores remained stable for QoL, PF, and fatigue for patients in the surgery (*N* = 79) and the systemic (*N* = 8) groups. In contrast, SRT patients (*N* = 122) reported worse mean scores for PF and fatigue, whereas WBRT patients (*N* = 105) reported worse mean scores for QOL, PF, and fatigue. Attrition precluded follow-up analysis in the BSC group (Supplementary Table S14).

## Discussion

In this study of 912 patients with first-time BM, 30% of the patients died within 3 months and 50% within 6 months after the BM diagnosis. Patients with survival <6 months and/or ECOG 2–4 reported worse scores for important PRO items such as overall QoL, PF, and fatigue over time. Most patients received anticancer treatment (ACT), the majority radiotherapy (RT). The justification of such treatments towards end of life must be questioned. The results of the present study should bring BM treatment guidelines to state that ACT, RT in particular, is to be avoided for patients with an estimated survival <6 months. This proposal is radical but important to avoid time-consuming and burdensome treatment at end of life.

Overall, treatment decisions in this study were in agreement with international and Norwegian guidelines available during the study period (2017–2021)^1^^,^^2^ and their recent updates.^3^, ^4^, ^5^ Surgery was predominantly offered to symptomatic patients in good PS with controlled ECM; these patients had relatively long survival and stable or improved PROs over time. On the other hand, although not demonstrated by RCTs, SRT may replace surgery in many patients who do not need immediate relief of raised intracranial pressure. In the present study, SRT could most likely have been an alternative for some of the patients in the Surgery group. The present study was not designed to address this question.

In the guidelines, post-operative RT, preferably SRT, is recommended.^1^, ^2^, ^3^, ^4^, ^5^ The Norwegian breast cancer guideline recommends RT within 30 days after resection, as suggested in one RCT.^15^ In the present study, only 78% had post-operative RT, few within 30 days, and 24% had post-operative WBRT. Our study design precludes definite analysis of these guideline deviations.

For patients unsuitable for surgery,^10^ our study indicates that decisions regarding SRT or WBRT were mainly based on number of BM and, to a lesser degree, PS. Although RCTs have demonstrated equal survival, SRT is preferred over WBRT for patients with 1–4 BM and ECOG 0–2/KPS ≥ 70, due to a higher risk of cognitive decline associated with WBRT.^1^, ^2^, ^3^, ^4^, ^5^^,^^18^, ^19^, ^20^ Accordingly, the majority of patients treated with WBRT had ≥5 BM, although for some patients in the WBRT group with 1–4 BM, SRT could most likely have replaced WBRT. We can only speculate why patients with ECOG 3–4 were referred to and accepted for RT. Doctors may wish to try any available treatment option, unfortunately often focusing most on treating the tumor and less on patient centered issues. The consequence may be a too optimistic attitude to the effects of RT. Also, as PS is often not assessed systematically between referral and admission, a deterioration in PS may have been overlooked by the RT center.

Survival was particularly short in the WBRT group. A large RCT published in 2016 (the QUARTZ study) demonstrated equal survival and quality-adjusted life-years (QALYs) for BSC alone compared to WBRT plus BSC for NSCLC patients not candidates for surgery or SRT.^10^ As a non-randomized, observational study, our study cannot determine whether patients with short survival would have worse, equal, or better PRO scores after two months without RT/WBRT. However, most patients in the WBRT group and a substantial proportion of the SRT group, especially those with ECOG 2–4, reported a decline in quality of life after ACT. Despite the limitations in an observational design it is difficult to argue for any clinical benefit of the ACT in this sub-cohort of patients. On the other hand, we cannot rule out that selected patients may have improved neurologic symptoms from RT, *i.e.,* for specific symptom causing BM lesions treatable by SRT. Nevertheless, fatigue and drowsiness are symptoms frequently reported as troublesome by patients with advanced cancer. Although disease progression may cause most of these symptoms, our study indicates increased symptom levels in these domains after radiotherapy, particularly WBRT. As no further RCTs on WBRT versus BSC are likely (with reference to attempts having failed^35^ and the struggle of recruiting patients to the QUARTZ study), we believe our study represents the best and most recent empirical evidence for not exposing patients with short expected survival to RT, WBRT in particular but probably also SRT. Both the survival- and PRO analyses in our study indicate that the findings of the QUARTZ study may also apply to non-lung primary cancer entities.

Patient selection to systemic treatments was also according to guidelines, with a lower proportion of controlled ECM and a higher proportion of non-symptomatic patients compared to the other treatment groups. Patients with melanoma or lung cancer constituted the majority of this treatment group, reflecting the documented anticancer effects for immune therapy and targeted therapies in these cancers.^3^^,^^4^ Short survival in the BSC group shows that the most vulnerable patients were correctly offered BSC alone; a pattern in selection criteria was difficult to identify in this rather small group.

SRT is increasingly offered to patients with >4 BMs, as it may be considered less time-consuming and burdensome and with lower risk for neurocognitive side effects compared to WBRT. The updated guidelines (ASTRO/ASCO-ANO-ASTRO/EANO-ESMO),^3^, ^4^, ^5^ mentions SRS/SRT as an option for patients with >4 BM, but the strength of the recommendations is weak (evidence low, recommendation weak,^4^ EANO: II, B; ESMO: II, B^3^). The guidelines also emphasize the importance of survival estimation in this regard (“such that they will experience those benefits”),^4^ “favorable prognosis”,^5^ and/or “good PS” (*i.e.,* ECOG ≥ 2/KPS ≥ 70).^3^, ^4^, ^5^ These recommendations are informed by prospective or retrospective observational studies,^36^, ^37^, ^38^, ^39^ with a majority of lung cancer patients and ECOG 0–2/KPS ≥ 70. In all these studies, higher KPS and status of extra-cranial disease are factors associated with superior survival, in line with the findings in our study.

The risk for neurocognitive side effects after SRS/SRT is not negligible. In a study comparing SRS to SRS + WBRT to patients with 1–3 BM,^19^ the proportions of patients with cognitive decline at 3 months were 64% vs. 92%, respectively. In a study comparing postoperative SRS with WBRT,^20^ time to cognitive deterioration was 3.7 vs. 3.0 months—statistically significant, but with marginal clinical relevance.

While technically feasible, the evidence supporting SRS/SRT to >4 BM to patients with an estimated survival less than six months is limited. Our results show that many patients with ECOG 2–4 are at risk for short survival, especially those with uncontrolled ECM and >4 BM. They also indicate that patients with survival <6 months do not benefit from ACT. Although SRT is less time consuming for the patient with usually less severe acute side-effects than WBRT, it still demands travel and time for the patient and an unsustainable financial, technical and human resources cost for the health care system. Thus, the question whether patients should be exposed to ACT at all near end of life still remains. A continuous focus on (futile) ACT may displace important planning and talks about end-of-life care, risking to delay advance care planning that is proved to reduce symptom burden towards death both for the patients and the next-of-kin. The resources used for futile ACT should rather be directed to these processes.

PS and status of extracranial disease (ECD) have long been suggested as important prognostic factors for patients with BM.^40^^,^^41^ In our study, ECOG PS, status of ECM, and molecular alterations were strong predictors for survival, compliant to previous publications. A recent publication from this study highlighted the impact of PS on survival after RT for BM in non-small cell lung cancer patients^42^ whereas in a retrospective analysis in breast cancer patients with BM, KPS ≤ 60 correlated with shorter brain-specific progression-free survival.^43^ In a meta-analysis with >5000 patients with BM with various primary cancers, patients with stable ECD had better OS compared to patients with progressive or unstable ECD,^7^ and a retrospective study with 1281 patients with various primary cancers found that KPS 90–100, controlled ECD, and targetable alterations were associated with long-term (>5 year) survival.^44^

The reasons for patients being offered futile treatments are complex. The scientific evidence for ACT for the substantial group of BM patients with short life time expectancy is limited.^10^^,^^45^ Consequently, the strength of the corresponding guideline recommendations for these patients are weak,^1^, ^2^, ^3^, ^4^, ^5^ and presented as “omission of WBRT should be considered”.^3^^,^^5^ Combined with limited precision in prognostic estimates and many doctors having challenges with end-of-life discussions, wishing to prolong life at almost all cost,^46^ this may entail a “treatment-prone” culture. Currently, the updated ASTRO-SNO-ASTRO guideline is the most clear, stating that radiotherapy should not be offered to patients with asymptomatic brain metastases and KPS ≤ 50 or less, or KPS < 70 and no systemic therapy options.^4^

International BM treatment guidelines define “good” PS as KPS ≥ 70,^1^^,^^4^ ECOG 0–2^5^ or do not provide a clear definition at all.^2^^,^^3^ The prognostic RPA- and DS-GPA classifications incorporates KPS < 70 as a poor prognostic factor.^6^^,^^8^ Equivalent ECOG and KPS scores have been debated. ECOG 2 has been suggested equivalent to KPS scores ranging from 50 to 70.^31^^,^^47^^,^^48^ Our study contributes evidence to support guideline recommendations to refrain from RT to patients with ECOG 3–4/KPS < 70.^2^^,^^4^ However, it also strongly indicates that ECOG 2 should be regarded as “poor” PS and a risk factor for short survival equivalent to ECOG 3–4/KPS < 70.

Our study confirms the prognostic potential of the DS-GPA^8^ and the RPA.^6^ However, a simplified approach using ECOG, ECM status, and number of BM provided survival estimates in the same range as DS-GPA, indicating that such a simplified scoring may replace the somewhat cumbersome DS-GPA calculations in the treatment decision making process. This simplified score is an ad-hoc analyses and needs further evaluation and validation, which will be subject for up-coming analyses of our study, together with evaluations of the predictive performance of different prognostic scores. In multivariable analyses of the total patient cohort in our study, presence of targetable mutations was associated with superior survival. This is also in line with the DS-GPA.^8^ This indicates that the potential for obtaining control of both intra- and extracranial disease with further systemic treatment should be considered in BM treatment decision making.

Presentation of mean PRO scores was restricted to 2 months follow-up due to high attrition at later time points, especially in patients with short survival. Patients with survival <6 months from BM diagnosis experienced poorer QoL, PF, and more fatigue over time, particularly patients with ECOG 2–4 in the WBRT group. Furthermore, patients surviving >6 months displayed stable or improved symptom levels. Whereas a systematic review reported stable or improved symptoms over time after SRT,^30^ prospective studies on QoL after WBRT report worse scores for several domains, most consistently fatigue.^23^, ^24^, ^25^, ^26^, ^27^, ^28^ However, in two recent studies, long-term survivors after WBRT (defined as >3 months in these studies) tended to report stable or improved levels for several QoL domains,^28^^,^^29^ high-lightning the importance of patient selection concerning ACT. With estimated survival >6 months, patients may experience stabilization or improvement of symptoms. However, with expected survival <3–6 months, our data indicate that intracranial ACT does not relieve symptoms but probably inflict acute side-effects, increased symptom burden and steal valuable time for the patient away from home and family.

There are indications that a ≥10 point numerical change in PRO scores may be considered clinically meaningful.^34^ Therefore, such changes are presented in this study, although a numerically similar change in two or more domains may represent very different clinical effects for the patient (*i.e.,* vomiting versus emotional function).

Analyzing PROs retrospectively according to survival may confer confirmation bias. Our presentations of results should be understood as descriptive for defined patient subgroups to provide support to treatment decision-making processes. The low patient number responding at month 2, especially in the ECOG 3–4 groups, indicates high attrition. The attrition in the present study aligns with previous prospective BM studies reporting PRO data, with 20–62% of patients completing questionnaires at one or three months,^23^, ^24^, ^25^, ^26^^,^^28^ including trials with selected patients in good PS.^49^ A short survival after the last response indicate rapid deterioration and death as probable reasons for attrition in our cohort, supported by the relatively low attrition among responders surviving >12 months. As the “most fit” patients are likely to respond, symptom scores may be biased towards better levels. Towards the end of life, patients will often prefer to stay at home and to be with their family. By adding systematic use of PROs to the decision making, informed wishes and preferences of the patient and family members can and must be explored and acknowledged by the treatment team leading to honest—but challenging—discussions about end-of-life care.

Strengths of this study include the prospective design, and a large, population-based patient cohort with PROs assessments over a long follow-up period. Its limitations are follow-up based on data from routine clinical visits and that the majority of patients were recruited after referral to RT centers, which may confer referral bias. With more patients recruited at primary hospitals, including more patients with poorer PS and those selected for BSC alone, the result could have been lower survival estimates whereas including more patients selected for initial systemic treatment would potentially result in better survival. Another limitation is the high proportion of patients assigned to WBRT, that now may be offered SRT instead, even with >4 BM. As the vast majority of BM patients are still considered for RT, our survival results in a large proportion of these patients may be considered population based and informative in future treatment selections. The high attrition rate in completion of PROs among the different subgroups limits the strength of the findings in these groups but is indicative of the vulnerability of many of patients with BM.

In conclusion, the effects of ACT on symptoms and function for patients with short expected survival (<6 months) are questionable. Clinicians must carefully consider how to apply ACT to these patients. Patients with ECOG 0–1 are likely to have an expected survival time sufficient to benefit from intracranial ACT. For patients with ECOG 3–4, expected survival is too short to justify ACT, although some patients with localized, symptom-causing lesions may be considered for surgical resection or SRT. Patients with ECOG 2, especially those with uncontrolled extra-cranial disease, limited options for further systemic ACT, and/or five or more BM, should be carefully evaluated in order to prevent futile ACT, particularly radiotherapy. Our data suggest that BM treatment guidelines need to be revised accordingly. Structured, systematic consultations, digitally supported with PROMS and up-to-date guidelines, may be a way forward to improve BM treatment decision making.

## Contributors

OEY, NA, JHL, MJH, and SK conceptualized, designed, and supervised the study. OEY, GLA, and ATK have directly accessed and verified the underlying data reported in the manuscript. OEY, GLA, and ES conducted the data analyses. OEY, GLA, and SK drafted the manuscript. OEY, GLA, ATK, RvH, KMH, JÅL, ØP, MIT, and RRW included patients and provided data to the study. All authors contributed to the interpretation of results and critically reviewed the manuscript and accept responsibility for the decision to submit for publication.

## Data sharing statement

The data underlying the results reported in this Article, as well as the study protocol and statistical analysis plan, will be made available after deidentification immediately after publication and for 3 years subsequently. Researchers who provide an acceptable proposal and purpose can contact olavy@ous-hf.no. To gain access, data requestors will need to sign a data exchange agreement that requires approval by the Data Protection Official at the Oslo University Hospital. Anonymized data subsets may be requested and can be uploaded to a suitable repository.

## Declaration of interests

All authors report funding of study/research infrastructure by the Norwegian South-East Regional Health Authority and The Norwegian Cancer Society, and reimbursement of article processing charges by the University of Oslo, Norway.

## Appendix A Supplementary data

Supplementary Material

Study Protocol
