# Supplementary material for: Survival and quality of life after first-time diagnosis of brain metastases: a multicenter, prospective, observational study
Source: Lancet Reg Health Eur. 2024 Dec 19;49:101181. doi: 10.1016/j.lanepe.2024.101181 (PMC11728971; doi:10.1016/j.lanepe.2024.101181)
Supplement: Supplementary Material [file mmc1.docx]

**Supplementary material**

**Contents**

**Details on the EORTC questionnaires EORTC QLQ-C15-PAL and EORTC QLQ-BN20………………………2**

**Supplementary Figure 1. Inclusion flowchart……………………………………………………………………….3**

**Supplementary Figure 2.** **Initial brain metastases treatment…………………………………………………..…..4**

**Supplementary Figure 3. Kaplan-Meier curves for survival by prognostic scores……………………………….5**

**Supplementary Table 1. Data item list and definitions ....………………………………………………………….8**

**Supplementary Table 2. Detailed information on all primary cancers……………………………………..……10**

**Supplementary Table 3. Uni- and multivariable analyses for the surgery, SRT, and WBRT groups……...….11**

**Supplementary Table 4. Median overall survival (95%CI) per treatment group and ECOG-status………….12**

**Supplementary Table 5. Prognostic scores. DS-GPA, RPA, and simplified score for lung-, colorectal-, breast-, kidney cancer, and melanoma………………………………………………………………………………………13**

**Supplementary Table 6. Patient-reported outcomes from inclusion to 12 months, by treatment group………14**

**Supplementary Table 7. Number of study participants and responders at each assessment (attrition)……….16**

**Supplementary Table 8. Change in PROs from inclusion to month 2 for complete responders at M0-M2, by ECOG-status at inclusion, surgery group……………………………………………………………………...…..17**

**Supplementary Table 9. Changes in PROs from inclusion to month 2 for complete responders at M0-M2, by ECOG-status at inclusion, SRT group……………………………………………………………………………...19**

**Supplementary Table 10. Changes in PROs from inclusion to month 2 for complete responders at M0-M2, by ECOG-status at inclusion, WBRT group…………………………………………………………………….…….21**

**Supplementary Table 11. Changes in PROs from inclusion to month 2 for complete responders at M0-M2 by survival groups according to survival after date of BM diagnosis………………………………………………..23**

**Supplementary Table 12. Change in patient-reported outcomes from inclusion to month 2 for complete responders at M0-M2, by survival groups according to survival after date of BM diagnosis, lung cancer group …………………………………………………………………………………………………………………………25Supplementary Table 13. Change in patient-reported outcomes from inclusion to month 2 for complete responders at M0-M2, by survival groups according to survival after date of BM diagnosis, non-lung cancer group…...……………………………………………………………………………………………………………..26**

**Supplementary Table 14. Change in PROs from inclusion to month 2 for complete responders at M0-M2, by treatment group…………………………………………………………………………………………………...…27**

**Supplementary Table 15. Change in patient-reported outcomes from inclusion to month 2 for complete responders at M0-M2, by ECOG-status at inclusion, lung cancer group…………………………………….….29**

**Supplementary Table 16. Change in patient-reported outcomes from inclusion to month 2 for complete responders at M0-M2, by ECOG-status at inclusion, non-lung cancer group…………………………………..30**

**Supplementary Table 17 Change in patient-reported outcomes from inclusion to month 2 for complete responders at M0-M2, by DS-GPA group………………………………………………………………………….31**

**Supplementary Table 18. Change in patient-reported outcomes from inclusion to month 2 for complete responders at M0-M2, by RPA group………………………………………………………………………………32**

**Supplementary Table 19. Change in patient-reported outcomes from inclusion to month 2 for complete responders at M0-M2, by Simplified score group………………………………………………………………….33**

**Details on the EORTC questionnaires EORTC QLQ-C15-PAL and EORTC QLQ-BN20**

The EORTC questionnaire for palliative care (EORTC QLQ-C15-PAL) (1) comprises 15 items assessing physical and emotional function (PF/EF), seven symptom scales (fatigue, pain, nausea/vomiting, dyspnea, sleep disturbances, appetite loss and constipation) and global QoL (qQoL). The diagnosis-specific module EORTC QLQ-BN20, (2) originally developed for primary brain tumors, has 13 items comprising four scales (visual disorder, motor dysfunction, communication deficits and future uncertainty), and seven symptom items. Three scales (visual disorder, motor dysfunction, communication deficits) and four symptom items (headaches, seizures, drowsiness, weakness of legs) were used in this study. All items are scored on 1–4-point Likert scales except the overall QoL going from 1–7. Scores are linearly transformed to 0–100 scales. A higher score indicates better overall QoL and PF/EF, but higher symptom burden for the symptom scales. Both questionnaires were scored based on EORTC scoring procedure (<https://www.eortc.org/app/uploads/sites/2/2018/02/SCmanual.pdf>) and missing item responses were handled according to the EORTC scoring manual. At least 50% of items in each scale must be completed in order to produce a score, meaning that if one or more items are missing, the score is set to missing for all scales except the EORTC QLQ-C15-PAL pain scale, and the EORTC QLQ-BN20 visual disorder, motor dysfunction, and communication deficit scales.A ≥10 point change in score is considered clinically meaningful, i.e. perceptible to patients. (3) The EORTC QLQ-C15-Pal has been used extensively and has shown good measurement properties, including content and construct validity, internal consistency, absolute measurement error and interpretability (1, 4). The EORTC QLQ-BN20 has been widely used in trials with BM patients as it has shown good measurement properties in primary brain cancer patients. (2, 5) The C15-PAL and BN20 have been validated in BM patients. (6)

1. Groenvold M, Petersen MA, Aaronson NK, Arraras JI, Blazeby JM, Bottomley A, et al. The development of the EORTC QLQ-C15-PAL: a shortened questionnaire for cancer patients in palliative care. European journal of cancer. 2006;42(1):55-64.

2. Osoba D, Aaronson NK, Muller M, Sneeuw K, Hsu MA, Yung WK, et al. The development and psychometric validation of a brain cancer quality-of-life questionnaire for use in combination with general cancer-specific questionnaires. Quality of life research : an international journal of quality of life aspects of treatment, care and rehabilitation. 1996;5(1):139-50.

3. Osoba D, Rodrigues G, Myles J, Zee B, Pater J. Interpreting the significance of changes in health-related quality-of-life scores. Journal of clinical oncology : official journal of the American Society of Clinical Oncology. 1998;16(1):139-44.

4. van Roij J, Fransen H, van de Poll-Franse L, Zijlstra M, Raijmakers N. Measuring health-related quality of life in patients with advanced cancer: a systematic review of self-administered measurement instruments. Quality of life research : an international journal of quality of life aspects of treatment, care and rehabilitation. 2018;27(8):1937-55.

5. Ģiga L, Pētersone A, Čakstiņa S, Bērziņa G. Comparison of content and psychometric properties for assessment tools used for brain tumor patients: a scoping review. Health and quality of life outcomes. 2021;19(1):234.

6. Caissie A, Nguyen J, Chen E, Zhang L, Sahgal A, Clemons M, et al. Quality of life in patients with brain metastases using the EORTC QLQ-BN20+2 and QLQ-C15-PAL. International journal of radiation oncology, biology, physics. 2012;83(4):1238-45.

**Supplementary Figure 1. Inclusion flowchart**

1406 patients screened

Patients excluded:

- 35 previous BM
- 6 withdrew consent
- 8 too long time interval from diagnosis to inclusion
- 11 unconfirmed brain metastases
- 6 other primaries (primary CNS tumors (5), CNS lymphoma (1) )
- 1 dural metastases only
- 1 unknown

Patients not included:

- 42 declined participation
- 22 not sufficient Norwegian skills
- 108 logistic reasons
- 67 deemed too poor to participate
- 24 during corona lock-down
- 124 other/unknown

912 patients included in final analyses

980 patients included

**Supplementary Figure 2.** **Initial brain metastases treatment**.


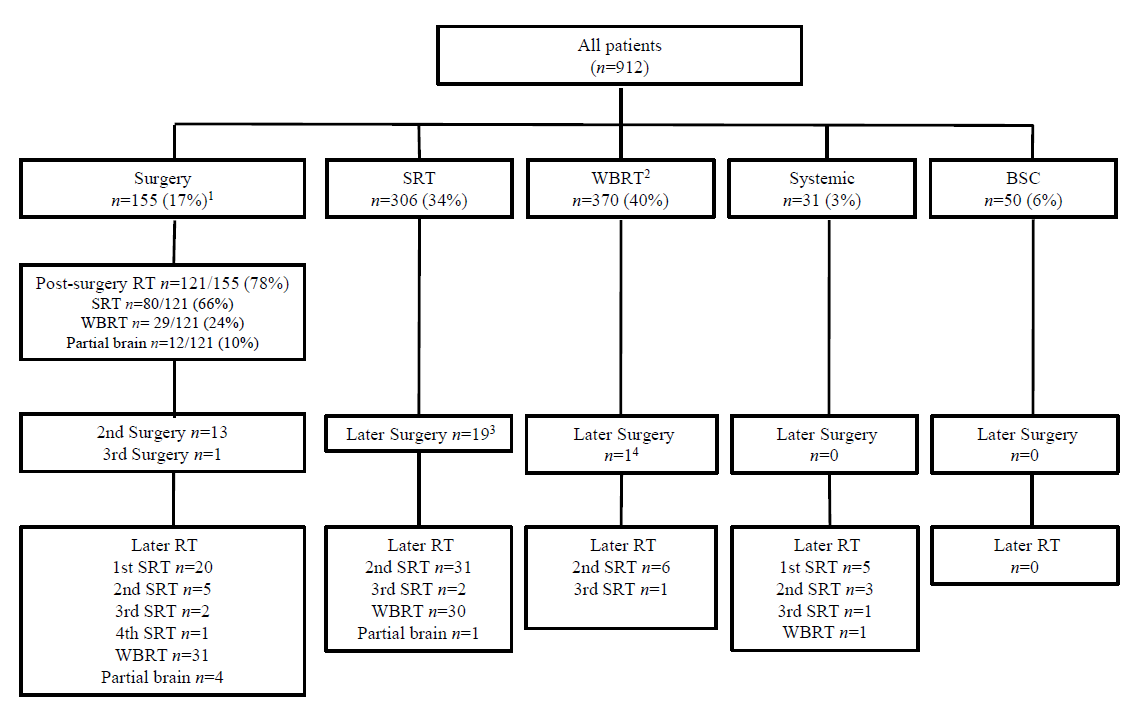


**Initial brain metastases treatment**. Initial BM treatments. SRT: Stereotactic radiotherapy, WBRT: Whole brain radiotherapy, Systemic: Systemic treatment, BSC: Best supportive care, RT: Radiotherapy. ^1^Including one patient with biopsy only. ^2^Including four patients treated with partial brain RT (none of these had later RT). ^3^One patient had second surgery. ^4^After partial brain as initial treatment.

**Supplementary Figure 3**

A)


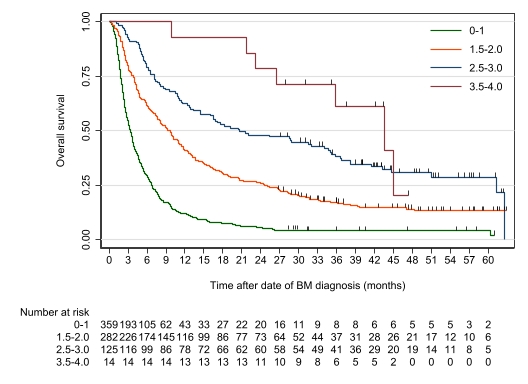


B)


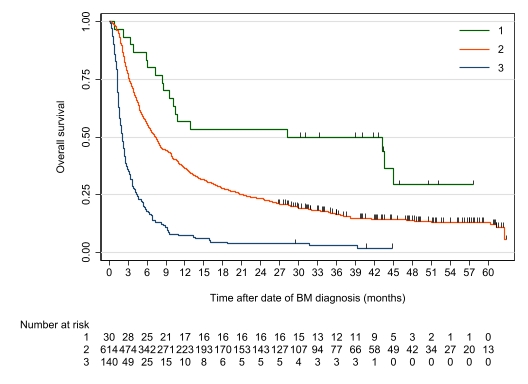


C)


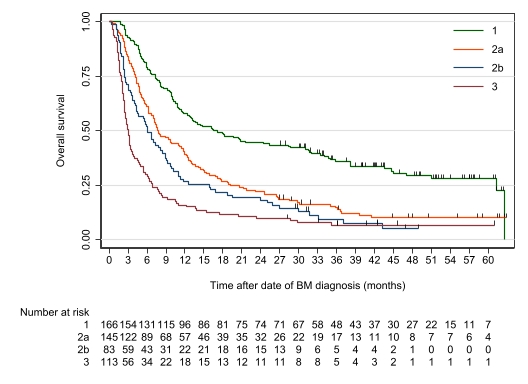


D)


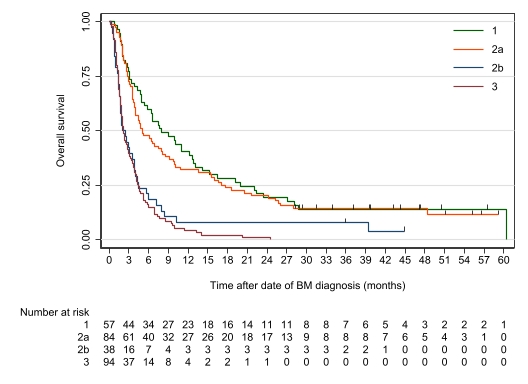


**Kaplan-Meier curves for survival by prognostic scores.** DS-GPA score, RPA class and simplified scores across treatment groups for lung-, breast-, colorectal-, kidney cancer, and melanoma patients. Patients are stratified by DS-GPA score, RPA class and simplified scores for patients with 1-4 and ≥5 BM. Median overall survival (mOS) after BM diagnosis and 95% confidence intervals (95% CI). A) Survival by DS-GPA score (N=780): 0-1: 3.4 (2.9-3.8), 1.5-2.0: 9.3 (7.5-11.0), 2.5-3.0: 20.6 (10.4-30.9), 3.5-4.0: 43.6 (29.2-58.1). p>0.001. B) Survival by RPA class (N=784): Class 1: 28.2 (0.0-73.2), Class 2: 7.4 (6.5-8.2), Class 3: 2.1 (1.6-2.6). p<0.001. C) Survival by simplified score, 1-4 BM (N=507): Group 1: 16.5 (10.5-22.5), Group 2a: 7.4 (6.0-8.8), Group 2b: 6.1 (4.6-7.6), Group 3: 3.0 (2.5-3.5). p<0.001. D) Survival by simplified score, ≥5 BM (N=273): Group 1: 7.9 (4.2-11.6), Group 2a: 4.0 (3.2-4.8), Group 2b: 2.1 (1.0-3.1), Group 3: 2.2 (1.8-2.6), p<0.001. Survival calculated by Kaplan-Meier analyses. mOS: Median overall survival. CI: Confidence interval. BM: Brain metastases. ECM: Extra-cranial metastases. DS-GPA: Disease-specific graded prognostic assessment. RPA: Recursive partitioning analysis. Simplified scores: 1: ECOG 0-1/controlled ECM, 2a: ECOG 0-1/ECM uncontrolled, 2b: ECOG 2-4/ECM controlled, 3: ECOG 2-4/ECM uncontrolled.

**Supplementary Table 1. Data item list and definitions**

| **Variable** | **Entry/definition** | **Assessed at**  **(I=Inclusion, F= follow-up)** |
| --- | --- | --- |
| **Listed in Table 1** | | |
| **Sex** | Male, female | I |
| **Age** | Age calculated from year of birth to year of brain metastasis diagnosis | I |
| **Primary cancer** | Primary cancer from which brain metastases originates.  “Other”: Gynecological (n=23: Ovary [*n*=11], tube [*n*=5], uterus [*n*=4], cervix [*n*=3]), esophageal (*n*=12), pancreatic (*n*=5), gastric (*n*=2), duodenal (*n*=1), GIST (*n*=1), urothelial (*n*=12), prostate (*n*=9), testicular (*n*=5), ear-nose-throat (*n*=9), sarcoma (*n*=3), thyroid (*n*=2), non-mela*n*oma skin (*n*=2), anal (*n*=1), unknown primary (*n*=25) | I |
| **Number of brain metastases** | Number of brain metastases described on diagnostic intracranial radiology (CT and/or MRI). If further radiology during treatment planning revealed additional brain metastases, the number was not changed | I |
| **Eastern Cooperative Oncology Group (ECOG)-status** | 0: Fully active, no performance restrictions  1: Physical activity restricted; fully ambulatory and able to carry out light work  2: Full self-care capability, unable to carry out any work activities. Ambulatory >50% of time awake  3: Limited self-care capability, confined to bed or chair >50% of time awake  4: Completely disabled. No self-care capability. Totally bed- or chairbound  Adapted from: Oken MM, Creech RH, Tormey DC, et al. Toxicity and response criteria of the Eastern Cooperative Oncology Group. Am J Clin Oncol 1982; 5:649. | I, F |
| **Extracranial disease (ECD) status** | Status of extracranial disease (ECD; primary cancer and metastases to organs outside CNS) at evaluation closest in time to brain metastasis diagnosis. Controlled: No evidence of ECD, stable disease or response to treatment. Unstable: ECD diagnosed at time of brain metastasis diagnosis (concomitant), progressive disease and/or unknown status | I |
| **Symptomatic brain metastases** | Yes: Brain metastases diagnosed due to symptoms suspicious of brain metastases  No: Brain metastases diagnosed without symptoms (i.e due to metastases screening, accidentally, screening for other study participation, etc) | I |
| **Steroids** | Use of steroids for intracranial symptoms at time of inclusion or follow-up | I, F |
| **Targetable mutations** | Mutation or positivity for at least one of the following: ALK, EGFR, BRAF, HER-2, KRAS, MSI, NRAS, PD-L1, Progesterone, ROS1, Estrogen confirmed by pathology reports |  |
| **Data collection – demographics and background** | | |
| **Registration date** | Date of registration of data in question | I, F |
| **Date of clinical visit** | Date of consultation from which data are collected | F |
| **Height** | Cuurent height (cm) at inclusion | I |
| **Weight** | Current weight (kg) at consultation | I, F |
| **Living conditions** | Home (house, appartment, etc), institution | I, F |
| **Care needs** | Need for help in daily activities (home nursing, next-of-kin, others, none) | I, F |
| **Assistance from palliative care teams since previous follow-up?** | Yes/No | I, F |
| **Hospital admissions since previous follow-up?** | Yes/No. If yes: Number of admissions | F |
| **Co-morbidity** | According to Charlson co-morbidity index: Charlson ME, Pompei P, Ales KL, MacKenzie CR. A new method of classifying prognostic comorbidity in longitudinal studies: development and validation. J Chronic Dis. 1987;40(5):373-83 | I |
| **Data collection – brain metastases diagnosis and follow-up** | | |
| **Biochemistry** | Hemoglobin (g/dL), C-reactive protein (mg/L), lactate dehydrogenase (U/L), albumin (g/L). Date of sampling (no more than 2 weeks before or after date of registry) | I, F |
| **If symptomatic brain metastases before diagnosis, which symptoms?** | Headache, nausea/vomiting, seizures, paresis of hand/arm/leg, hemiparesis, hemiparalysis, facial nerve paresis, sensory deficits, visual, unsteadiness, cognitive, personality changes, dizziness, aphasia, memory, others | I |
| **Date of brain metastasis diagnosis** | Date of first cerebral CT and/or MRI describing brain metastases | I |
| **Localization of brain metastases** | Supra/infratentorial, lobe, leptomeningeal | I |
| **Diameter of the largest brain metastasis** | Diameter of largest brain metastasis (mm) on diagnostic cerebral radiology | I |
| Use of steroids at time of inclusion. If «Yes», describe type and dosage | Yes: Patient uses steroids at time of inclusion  No: Patient does not use steroids at time of inclusion  At SRT only: Patient using steroids only in relation to SRT procedures/treatment  Unknown: Information of steroid use missing | I |
| **Clinical signs of progression of brain metastases since previous follow-up?** | Does the patient record mention symptoms suspicious of progression of brain metastases? Yes/No | F |
| **Radiological progression of brain metastases since previous follow-up?** | Yes: Definite progression described from cerebral CT and/or MRI, OR re-evaluation reveals progression of brain metastases  No: No progression of brain metastases described  Not performed: If no cerebral radiology performed since last follow-up | F |
| **Date of radiological progression of brain metastases** | Date of definite or re-evaluated cerebral CT/MRI describing progression | F |
| **If radiological progression of brain metastases – location?** | Local – progression of known lesions  Distant – new lesions  Both: Both local and distant progression | F |
| Use of steroids within the last week before follow-up. If «Yes», describe type and dosage | Yes: Patient record describes use of steroids  No: Patient record describes no use of steroids  Unknown: Patient record does not contain any information on use of steroids | I, F |
| **Tumor-directed reatment (surgery, radiotherapy and/or systemic) of brain metastases since previous follow-up?** | Yes: Tumor-directed treatment given  No: No tumor-directed treatment given  If yes, indicate which treatment(s). If systemic tumor-directed treatment, state which type | F |
| **Status (alive or deceased)** | Alive or deceased | F |
| **Date of death** | If deceased, date of death | F |
| **Data collection – extracranial disease (primary tumor and metastases)** | | |
| **Primary cancer histology** | Adenocarcinoma, squamous, urothelial, small cell, neuroendocrine, germinal, unknown/other | I |
| **Status of primary cancer at inclusion** | Absent, stable, progressive, unknown | I |
| **Extracranial metastases at inclusion?** | Yes/no. If yes, date of diagnosis of first extracranial metastases | I |
| **Status of extracranial metastases at inclusion** | None, stable, progressive, concomitant, unknown | I |
| **Systemic treatment of primary cancer/extracranial metastases prior to inclusion** | Yes: Tumor-directed treatment given  No: No tumor-directed treatment given  If yes, state which type | I |
| **Clinical and/or biochemical signs of progression of extracranial disease since previous follow-up?** | Does the patient record mention symptoms or biochemistry suspicious of progression of extracranial disease? Yes/No | F |
| **Radiological progression of of extracranial disease since previous follow-up?** | Yes: Definite progression described from extracranial CT and/or MRI, OR re-evaluation reveals progression of extracranial disease  No: No progression of extracranial disease  described  Not performed: If no extracranial disease  radiology performed since last follow-up | F |
| **Date of radiological progression of extracranial disease** | Date of definite or re-evaluated extracranial CT/MRI describing progression | F |
| **Systemic tumor-directed treatment(s) of extracranial disease since previous follow-up?** | Yes: Tumor-directed treatment given  No: No tumor-directed treatment given  If yes, state which type | F |
| **Radiotherapy to extracranial organs since previous follow-up?** | Yes: Radiotherapy given (state which organs)  No: No radiotherapy given | F |

**Supplementary Table 2. Detailed information on all primary cancers (n = 912)**

| **Primary cancer** | ***N*** | **%** |
| --- | --- | --- |
| Lung  NSCLC  SCLC  Melanoma  Breast  Colorectal  Renal  Gynecological  Ovary  Tube  Uterus  Cervical  Upper GI tract  Esophagus  Pancreatic  Gastric  Duodenal  GIST  Urological  Urothelial  Prostate  Testicular  Unknown primary  Other  Head/neck  Sarcoma  Thyroid  Non-melanoma skin  Anal | 406  370  35  148  129  86  32  23  11  5  4  3  21  12  5  2  1  1  26  12  9  5  25  17  9  3  2  2  1 | 44·5  91·4  8·6  16·2  14·1  9·4  3·5  2·5  1·2  0·5  0·4  0·3  2·3  1·3  0·5  0·2  0·1  0·1  2·9  1·3  1·0  0·5  2·7  1·9  1·0  0·3  0·2  0·2  0·1 |

NSCLC: Non-small cell lung cancer. SCLC: Small-cell lung cancer. GI: Gastrointestinal. GIST: Gastrointestinal stromal tumor

**Supplementary Table 3 Uni- and multivariable analyses for the surgery, SRT, and WBRT groups**

|  | **Surgery patients (*N*=155)** | | | | | **SRT patients (*N*=306)** | | | | | | **WBRT patients (*N*=370)** | | | | |
| --- | --- | --- | --- | --- | --- | --- | --- | --- | --- | --- | --- | --- | --- | --- | --- | --- |
|  |  | **Cox proportional hazards model** | | | |  | **Cox proportional hazards model** | | | | |  | **Cox proportional hazards model** | | | |
|  |  | **Univariable** | | **Multivariable (*N*=153)** | |  | **Univariable** | | | **Multivariable (*N*=302)** | |  | **Univariable** | | **Multivariable (*N=*360)** | |
|  | *N* | **HR (95% CI)** | ***p*** | **HR (95% CI)** | ***p*** | ***N*** | **HR (95% CI)** | ***p*** | **HR (95% CI)** | | ***p*** | ***N*** | **HR (95% CI)** | ***p*** | **HR (95% CI)** | ***p*** |
| Age |  |  |  |  |  |  |  |  |  | |  |  |  |  |  |  |
| <70 | 93 | 1 |  | 1 |  | 149 | 1 |  | 1 | |  | 189 | 1 |  | 1 |  |
| ≥70 | 60 | 1·53 (1·05-2·22) | 0·027 | 1·27 (0·84-1·93) | 0·259 | 157 | 1·63 (1·27-2·09) | <0·001 | 1·45 (1·12-1·89) | | 0·005 | 181 | 1·46 (1·18-1·80) | <0·001 | 1·24 0·98-1·56) | 0·078 |
| Sex |  |  |  |  |  |  |  |  |  | |  |  |  |  |  |  |
| Male | 65 | 1 |  | 1 |  | 156 | 1 |  |  | |  | 161 | 1 |  | 1 |  |
| Female | 90 | 0·71 (0·49-1·03) | 0·067 | 0·68 (0·44-1·04) | 0·074 | 150 | 0·86 (0·67-1·10) | 0·218 | 0·99 (0·76-1·30) | | 0·957 | 209 | 0·72 (0·59-0·89) | 0·003 | 0·78 (0·61-0·99) | 0·043 |
| ECOG |  |  |  |  |  |  |  |  |  | |  |  |  |  |  |  |
| 0-1 | 105 | 1 |  | 1 |  | 205 | 1 |  | 1 | |  | 181 | 1 |  | 1 |  |
| 2 | 34 | 1·07 (0·68-1·69) | 0·768 | 1·07 (0·67-1·72) | 0·776 | 64 | 1·87 (1·39-2·53) | <0·001 | 1·77 (1·31-2·41) | | <0·001 | 104 | 2·28 (1·76-2·95) | <0·001 | 2·26 (1·73-2·96) | <0·001 |
| 3-4 | 14 | 2·66 (1·47-4·82) | 0·001 | 2·94 (1·47-5·87) | 0·002 | 33 | 2·52 (1·72-3·70) | <0·001 | 2·50 (1·65-3·79) | | <0·001 | 77 | 3·07 (2·32-4·08) | <0·001 | 2·80 (2·08-3·77) | <0·001 |
| Missing | 2 |  |  |  |  | 4 |  |  |  | |  | 8 |  |  |  |  |
| Primary |  |  |  |  |  |  |  |  |  | |  |  |  |  |  |  |
| Breast | 25 | 1 |  | 1 |  | 30 | 1 |  | 1 | |  | 68 | 1 |  | 1 |  |
| Lung | 50 | 1·90 (1·01-3·56) | 0·046 | 0·98 (0·48-2·01) | 0·964 | 155 | 1·83 (1·15-2·91) | 0·010 | 1·53 (0·94-2·51) | | 0·090 | 161 | 1·36 (1·02-1·83) | 0·039 | 0·91 (0·64-1·28) | 0·572 |
| Colorectal | 23 | 3·92 (1·94-7·91) | <0·001 | 2·67 (1·23-5·81) | 0·013 | 29 | 3·34 (1·89-5·91) | <0·001 | 2·03 (1·07-3·86) | | 0·031 | 30 | 2·89 (1·84-4·52) | <0·001 | 1·38 (0·83-2·30) | 0·220 |
| Melanoma | 30 | 1·78 (0·90-3·54) | 0·098 | 0·85 (0·39-1·86) | 0·675 | 52 | 1·70 (1·00-2·87) | 0·049 | 1·29 (0·72-2·31) | | 0·394 | 48 | 1·66 (1·13-2·44) | 0·010 | 0·99 (0·64-1·53) | 0·964 |
| Kidney | 4 | 0·97 (0·27-3·47) | 0·960 | 0·46 (0·12-1·80) | 0·263 | 7 | 2·33 (0·99-5·48) | 0·053 | 1·26 (0·48-3·32) | | 0·638 | 17 | 1·42 (0·82-2·46) | 0·212 | 0·97 (0·52-1·82) | 0·935 |
| Others | 21 | 1·84 (0·88-3·83) | 0·104 | 1·04 (0·44-2·46) | 0·932 | 33 | 2·36 (1·35-4·11) | 0·003 | 1·53 (0·83-2·82) | | 0·178 | 46 | 1·57 (1·07-2·30) | 0·021 | 1·04 (0·66-1·63) | 0·871 |
| Number of BM |  |  |  |  |  |  |  |  |  | |  |  |  |  |  |  |
| 1 | 108 | 1 |  | 1 |  | 166 | 1 |  | 1 | |  | 28 | 1 |  | 1 |  |
| 2-4 | 37 | 1·14 (0·74-1·77) | 0·552 | 1·11 (0·66-1·87) | 0·697 | 134 | 1·18 (0·92-1·51) | 0·199 | 1·26 (0·96-1·64) | | 0·091 | 71 | 1·05 (0·67-1·64) | 0·837 | 1·26 (0·79-2·02) | 0·334 |
| ≥5 (incl lepto) | 10 | 1·46 (0·73-2·92) | 0·281 | 2·19 (0·98-4·90) | 0·056 | 6 | 0·90 (0·37-2·20) | 0·820 | 0·87 (0·34-2·24) | | 0·779 | 268 | 0·90 (0·61-1·34) | 0·612 | 1·08 (0·71-1·65) | 0·707 |
| Missing |  |  |  |  |  |  |  |  |  | |  | 3 |  |  |  |  |
| Symptomatic |  |  |  |  |  |  |  |  |  | |  |  |  |  |  |  |
| No | 9 | 1 |  | 1 |  | 76 | 1 |  | 1 | |  | 45 | 1 |  | 1 |  |
| Yes | 146 | 1·61 (0·66-3·96) | 0·295 | 1·55 (0·60-4·02) | 0·371 | 230 | 1·21 (0·91-1·62) | 0·192 | 1·08 (0·79-1·47) | | 0·635 | 325 | 1·16 (0·84-1·60) | 0·383 | 0·95 (0·68-1·34) | 0·785 |
| Status ECD |  |  |  |  |  |  |  |  |  | |  |  |  |  |  |  |
| Controlled | 108 | 1 |  | 1 |  | 142 | 1 |  | 1 | |  | 123 | 1 |  | 1 |  |
| Uncontrolled | 45 | 1·67 (1·13-2·48) | 0·010 | 1·63 (1·07-2·48) | 0·022 | 164 | 1·54 (1·20-1·98) | <0·001 | 1·67 (1·29-2·17) | | <0·001 | 247 | 1·53 (1·22-1·92) | <0·001 | 1·33 (1·04-1·68) | 0·02 |
| Targetable mutations* |  |  |  |  |  |  |  |  |  | |  |  |  |  |  |  |
| Yes | 77 | 1 |  | 1 |  | 167 | 1 |  | 1 | |  | 173 | 1 |  | 1 |  |
| No | 78 | 1·83 (1·26-2·66) | 0·002 | 2·03 (1·32-3·12) | <0·001 | 139 | 1·37 (1·07-1·75) | 0·012 | 1·16 (0·87-1·54) | | 0·323 | 197 | 1·36 (1·10-1·68) | 0·004 | 1·24 (0·97-1·59) | 0·080 |

Estimates from Cox proportional hazards models for the Surgery, SRT, and WBRT groups. The multivariable model comprises all clinical variables included in the unadjusted models. HR: Hazard ratio. CI: Confidence interval.SRT: Stereotactic radiosurgery. WBRT: Whole brain radiotherapy.

*Mutation or positivity for at least one of the following: ALK, EGFR, BRAF, HER-2, KRAS, MSI, NRAS, PD-L1, Progesterone, ROS1, Estrogen

**Supplementary Table 4. Median overall survival (95%CI) per treatment group and ECOG-status.**

|  | **Treatment groups** | | | | | |
| --- | --- | --- | --- | --- | --- | --- |
|  | All patients (*N*=896) | Surgery (*N*=153) | SRT  (N=302) | WBRT* (*N*=362) | Systemic  (*N*=30) | BSC  (*N*=49) |
| **ECOG-status** | **Median overall survival, months (95% CI)** | | | | | |
| 0-1 | 9·9 (8·5-11·3) | 13·7 (8·4-18·9) | 12·0 (9·3-14·6) | 5·2 (4·0-6·4) | 12·2 (10·5-13·9) | 1·1 (0·6-1·5) |
| 2 | 4·0 (3·4-4·7) | 12·6 (0·0-30·2) | 6·1 (4·1-8·0) | 2·9 (2·3-3·5) | 8·0 (1·5-14·5) | 1·0 (0·4-1·5) |
| 3-4 | 2·2 (1·8-2·5) | 5·7 (0·0-11·5) | 3·7 (1·2-6·2) | 2·1 (1·5-2-7) | 2·8 (0·0-9·3) | 1·2 (1·1-1·4) |

Survival calculated using the Kaplan-Meier analysis. SRT: Stereotactic radiotherapy. WBRT*: Whole brain radiotherapy (partial brain (n=4) included). ECOG: Eastern Cooperative Oncology Group performance status. CI: Confidence interval.

**Supplementary Table 5. Prognostic scores. DS-GPA, RPA, and simplified score for lung-, colorectal-, breast-, kidney cancer, and melanoma.**

|  | **DS-GPA** | | | | |
| --- | --- | --- | --- | --- | --- |
|  | **DS-GPA (lung-, breast-, colorectal-, kidney cancer, and melanoma)** | | | | |
|  | *N* | mOS | OS ≤3 mo | OS ≤6 mo | Alive>1 yr |
| **Total N** | 780 | 6.1 (5.3-6.9) | 231/780 (30%) | 388/780 (50%) | 250/780 (32%) |
| **Class 1 (0.0-1.0)** | 359/780 (46%) | 3.4 (2.9-3.8) | 166/359 (46%) | 254/359 (71%) | 43/359 (12%) |
| **Class 2 (1.5-2.0)** | 282/780 (36%) | 9.3 (7.5-11.0) | 56/282 (20%) | 108/282 (38%) | 116/282 (41%) |
| **Class 3 (2.5-3.0)** | 125/780 (16%) | 20.6 (10.4-30.9) | 9/125 (7%) | 26/125 (21%) | 78/125 (62%) |
| **Class 4 (3.5-4.0)** | 14/780 (2%) | 43.6 (29.2-58.1) | 0/14 (0%) | 0/14 (0%) | 13/14 (93%) |

|  | **RPA** | | | | |
| --- | --- | --- | --- | --- | --- |
|  | **RPA (lung-, breast-, colorectal-, kidney cancer, and melanoma)** | | | | |
|  | *N* | mOS | OS ≤3 mo | OS ≤6 mo | Alive>1 yr |
| **Total N** | 784 | 6.0 (5.2-6.7) | 233/784 (30%) | 392/784 (50%) | 250/784 (32%) |
| **RPA Class 1** | 30/784 (4%) | 28.2 (0.0-73.2) | 2/30 (7%) | 5/30 (17%) | 17/30 (57%) |
| **RPA Class 2** | 614/784 (78%) | 7.4 (6.5-8.2) | 140/614 (23%) | 272/614 (44%) | 223/614 (36%) |
| **RPA Class 3** | 140/784 (18%) | 2.1 (1.6-2.6) | 91/140 (65%) | 115/140 (82%) | 10/140 (7%) |

|  | **Simplified score** | | | | |
| --- | --- | --- | --- | --- | --- |
|  | **Simplified score (lung-, breast-, colorectal-, kidney cancer, and melanoma)** | | | | |
|  | N | mOS | OS ≤3 mo | OS ≤6 mo | Alive>1 yr |
| **Total N** | 784 | 6.0 (5.2-6.7) | 233/784 (30%) | 392/784 (50%) | 250/784 (32%) |
| 1-4 BM | 507/784 (65%) | 7.8 (6.6-9.1) | 116/507 (23%) | 210/507 (41%) | 193/507 (38%) |
| ≥5 BM | 273/784 (35%) | 3.8 (3.2-4.4) | 115/273 (42%) | 178/273 (65%) | 57/273 (21%) |
| **Group 1** | 223/784 (28%) | 13.1 (9.7-16.6) | 25/223 (11%) | 58/223 (26%) | 119/223 (53%) |
| 1-4 BM | 166/223 (74%) | 16.5 (10.5-22.5) | 12/166 (7%) | 35/166 (21%) | 96/166 (58%) |
| ≥5 BM | 57/223 (26%) | 7.9 (4.2-11.6) | 13/57 (23%) | 23/57 (40%) | 23/57 (40%) |
| **Group 2** | 353/784 (45%) | 6.1 (5.2-7.1) | 93/353 (26%) | 174/353 (49%) | 109/353 (31%) |
| 1-4 BM | 228/353 (65%) | 7.4 (6.0-8.8) | 47/228 (21%) | 96/228 (42%) | 79/228 (35%) |
| ≥5 BM | 122/353 (35%) | 4.0 (3.2-4.8) | 45/122 (37%) | 75/122 (62%) | 30/122 (25%) |
| **Group 2a** | 232/784 (30%) | 7.1 (6.0-8.3) | 47/232 (20%) | 103/232 (44%) | 84/232 (36%) |
| 1-4 BM | 145/232 (63%) | 7.8 (5.7-9.8) | 23/145 (16%) | 56/145 (39%) | 57/145 (39%) |
| ≥5 BM | 84/232 (36%) | 5.1 (3.2-7.0) | 23/84 (27%) | 44/84 (52%) | 27/84 (32%) |
| **Group 2b** | 121/784 (15%) | 4.4 (2.9-5.9) | 46/121 (38%) | 71/121 (59%) | 25/121 (21%) |
| 1-4 BM | 83/121 (69%) | 6.1 (4.6-7.6) | 24/83 (29%) | 40/83 (52%) | 22/83 (27%) |
| ≥5 BM | 38/121 (31%) | 2.1 (1.0-3.1) | 22/38 (58%) | 31/38 (82%) | 3/38 (8%) |
| **Group 3** | 208/784 (27%) | 2.7 (2.3-3.1) | 115/208 (55%) | 160/208 (77%) | 22/208 (11%) |
| 1-4 BM | 113/208 (54%) | 3.0 (2.5-3.5) | 57/113 (50%) | 79/113 (70%) | 18/113 (16%) |
| ≥5 BM | 94/208 (45%) | 2.2 (1.8-2.6) | 57/94 (61%) | 80/94 (85%) | 4/94 (4%) |

Survival calculated by Kaplan-Meier analyses. mOS: Median overall survival. CI: Confidence interval. BM: Brain metastases. ECM: Extra-cranial metastases. DS-GPA: Disease-specific graded prognostic assessment. RPA: Recursive partitioning analysis. Simplified scores: 1: ECOG 0-1/controlled ECM, 2a: ECOG 0-1/ECM uncontrolled, 2b: ECOG 2-4/ECM controlled, 3: ECOG 2-4/ECM uncontrolled.

**Supplementary Table 6. Patient-reported outcomes from inclusion to 12 months, by treatment group.**

|  | **Treatment** | **Inclusion** | **Month 1** | **Month 2** | **Month 3** | **Month 6** | **Month 9** | **Month 12** |
| --- | --- | --- | --- | --- | --- | --- | --- | --- |
|  | *N* | | | | | | | |
| Number of responders | All patients | 708 | 445 | 374 | 291 | 218 | 164 | 126 |
|  | Surgery | 121 | 103 | 94 | 78 | 63 | 44 | 36 |
|  | SRT | 240 | 157 | 145 | 124 | 97 | 75 | 58 |
|  | WBRT | 309 | 164 | 120 | 75 | 47 | 38 | 25 |
|  | Systemic | 19 | 17 | 14 | 13 | 11 | 7 | 7 |
|  | BSC | 19 | 4 | 1 | 1 | ·· | ·· | ·· |
| EORTC scale |  | **Mean (SD)** | | | | | | |
| Overall QoL | All patients | 55·3 (25·5) | 57·0 (24·6) | 55·4 (24·8) | 58·8 (23·0) | 60·9 (23·7) | 64·5 (22·3) | 66·8 (19·3) |
|  | Surgery | 61·8 (24·6) | 63·8 (22·9) | 62·3 (24·3) | 62·2 (23·4) | 62·4 (22·0) | 68·3 (18·6) | 69·0 (18·6) |
|  | SRT | 56·6 (24·2) | 57·9 (23·2) | 57·3 (23·6) | 62·0 (20·9) | 58·9 (25·5) | 60·7 (23·2) | 64·7 (20·5) |
|  | WBRT | 52·9 (25·9) | 51·2 (25·0) | 46·8 (24·3) | 48·4 (23·3) | 60·3 (23·2) | 67·6 (24·8) | 64·4 (18·0) |
|  | Systemic | 56·1 (29·5) | 68·6 (22·7) | 67·9 (19·8) | 69·2 (21·4) | 71·2 (16·8) | 66·7 (13·6) | 81·0 (11·5) |
|  | BSC | 36·8 (25·2) | 29·2 (39·4) | ·· | ·· | ·· | ·· | ·· |
| Physical function | All patients | 68·8 (27·7) | 69·0 (27·6) | 69·1 (28·8) | 73·8 (26·9) | 76·4 (25·8) | 79·0 (23·6) | 83·4 (17·9) |
|  | Surgery | 75·4 (24·6) | 76·1 (23·5) | 78·7 (20·8) | 79·1 (22·6) | 79·0 (22·9) | 82·3 (23·0) | 84·0 (15·1) |
|  | SRT | 71·5 (26·6) | 72·7 (24·7) | 72·0 (25·5) | 75·2 (26·7) | 75·0 (27·9) | 78·6 (21·9) | 82·9 (17·7) |
|  | WBRT | 66·0 (27·8) | 60·9 (29·9) | 56·6 (33·7) | 63·7 (30·0) | 74·0 (26·0) | 76·3 (27·1) | 80·4 (23·0) |
|  | Systemic | 74·9 (22·8) | 79·7 (25·8) | 84·9 (17·8) | 84·6 (22·0) | 84·8 (20·0) | 76·2 (28·3) | 95·2 (8·7) |
|  | BSC | 32·7 (32·4) | 38·9 (43·0) | ·· | ·· | ·· | ·· | ·· |
| Emotional function | All patients | 76·0 (25·3) | 78·8 (25·5) | 79·2 (24·5) | 81·0 (23·2) | 81·5 (22·7) | 83·9 (20·0) | 85·1 (17·6) |
|  | Surgery | 79·9 (24·3) | 81·7 (22·9) | 81·7 (22·4) | 82·5 (19·9) | 83·6 (18·6) | 84·5 (20·8) | 86·6 (17·3) |
|  | SRT | 75·9 (24·9) | 81·1 (24·1) | 79·6 (24·0) | 80·8 (23·6) | 78·5 (25·2) | 81·8 (21·6) | 82·5 (18·8) |
|  | WBRT | 74·9 (25·7) | 74·7 (28·3) | 76·3 (27·2) | 78·4 (26·4) | 83·7 (23·7) | 86·9 (16·3) | 85·3 (16·2) |
|  | Systemic | 79·8 (23·9) | 80·4 (22·2) | 85·7 (18·3) | 88·5 (17·2) | 86·4 (14·6) | 88·1 (15·9) | 97·6 (6·3) |
|  | BSC | 68·4 (29·3) | 79·2 (25·0) | ·· | ·· | ·· | ·· | ·· |
| Fatigue | All patients | 42·8 (27·1) | 47·5 (27·6) | 47·5 (27·4) | 41·0 (26·8) | 37·0 (25·7) | 35·7 (28·0) | 28·6 (21·7) |
|  | Surgery | 36·6 (26·0) | 38·6 (22·6) | 40·7 (24·6) | 38·0 (25·2) | 37·3 (23·9) | 30·7 (29·4) | 27·8 (22·9) |
|  | SRT | 42·3 (26·0) | 44·7 (26·7) | 43·7 (25·7) | 37·3 (25·6) | 37·3 (27·7) | 39·3 (26·2) | 29·6 (22·5) |
|  | WBRT | 44·8 (27·4) | 56·8 (28·2) | 59·0 (27·6) | 53·1 (26·7) | 38·3 (24·8) | 36·5 (31·1) | 30·0 (20·4) |
|  | Systemic | 36·0 (26·8) | 32·4 (25·3) | 31·0 (27·6) | 24·4 (26·9) | 27·3 (21·4) | 23·8 (13·1) | 19·0 (11·5) |
|  | BSC | 62·3 (30·9) | 58·3 (50·0) | ·· | ·· | ·· | ·· | ·· |
| Nausea/vomiting | All patients | 14·1 (24·8) | 14·5 (23·0) | 18·2 (26·4) | 15·6 (25·1) | 11·5 (21·4) | 11·3 (21·7) | 9·7 (16·9) |
|  | Surgery | 11·6 (24·2) | 10·9 (20·6) | 13·3 (21·5) | 15·0 (26·7) | 11·8 (21·0) | 8·3 (17·8) | 6·5 (13·4) |
|  | SRT | 10·9 (22·2) | 11·1 (20·6) | 13·3 (22·7) | 10·1 (18·6) | 9·6 (21·5) | 12·6 (23·9) | 11·7 (19·4) |
|  | WBRT | 17·1 (26·5) | 20·4 (26·1) | 28·5 (30·9) | 26·2 (30·7) | 17·0 (22·9) | 11·7 (21·1) | 12·0 (16·3) |
|  | Systemic | 15·8 (23·2) | 9·8 (15·7) | 16·7 (28·5) | 10·3 (16·0) | 3·0 (10·1) | 14·3 (26·2) | 0 (0) |
|  | BSC | 19·3 (27·9) | 16·7 (19·2) | ·· | ·· | ·· | ·· | ·· |
| Pain | All patients | 26·6 (30·2) | 24·2 (27·7) | 26·0 (29·5) | 24·2 (27·2) | 21·9 (26·3) | 20·3 (25·2) | 14·6 (20·2) |
|  | Surgery | 21·8 (25·3) | 21·8 (26·0) | 23·7 (27·5) | 20·7 (24·8) | 19·0 (24·5) | 15·5 (21·7) | 14·4 (19·2) |
|  | SRT | 25·9 (28·8) | 25·9 (28·3) | 26·2 (28·9) | 24·8 (27·5) | 24·4 (27·0) | 23·3 (25·0) | 16·7 (22·3) |
|  | WBRT | 27·0 (31·5) | 23·7 (27·1) | 27·6 (31·6) | 27·3 (28·8) | 20·2 (28·0) | 21·5 (29·0) | 11·3 (17·2) |
|  | Systemic | 28·9 (32·8) | 22·5 (30·0) | 21·4 (24·8) | 23·1 (28·5) | 24·2 (22·8) | 11·9 (24·9) | 9·5 (18·9) |
|  | BSC | 57·9 (34·9) | 54·2 (45·9) | ·· | ·· | ·· | ·· | ·· |
| Dyspnea | All patients | 24·8 (29·5) | 29·3 (30·4) | 27·2 (29·3) | 24·8 (27·4) | 22·0 (24·7) | 20·8 (24·4) | 15·9 (22·7) |
|  | Surgery | 19·0 (27·6) | 23·6 (28·3) | 21·5 (26·3) | 22·2 (26·1) | 19·6 (21·3) | 18·6 (19·7) | 19·4 (23·1) |
|  | SRT | 26·8 (29·7) | 29·0 (30·0) | 26·4 (28·8) | 23·6 (26·2) | 25·0 (28·6) | 23·1 (27·9) | 14·4 (21·7) |
|  | WBRT | 25·1 (29·4) | 33·7 (32·0) | 32·5 (31·1) | 30·2 (29·6) | 19·9 (20·5) | 17·1 (21·7) | 14·5 (26·3) |
|  | Systemic | 26·3 (30·6) | 19·6 (23·7) | 23·8 (30·5) | 23·1 (31·6) | 18·2 (22·9) | 28·6 (23·0) | 14·3 (17·8) |
|  | BSC | 29·8 (35·0) | 41·7 (31·9) | ·· | ·· | ·· | ·· | ·· |
| Sleep disturbance | All patients | 34·1 (31·6) | 28·1 (29·1) | 25·1 (28·4) | 26·5 (29·2) | 22·6 (26·5) | 24·5 (28·0) | 20·0 (25·4) |
|  | Surgery | 36·1 (33·4) | 25·4 (27·9) | 25·8 (30·3) | 27·3 (31·9) | 23·8 (24·3) | 29·5 (28·9) | 23·1 (28·5) |
|  | SRT | 34·3 (30·7) | 26·1 (27·0) | 24·4 (25·2) | 27·6 (28·2) | 21·0 (26·5) | 25·3 (28·4) | 19·3 (24·4) |
|  | WBRT | 33·3 (31·8) | 32·7 (31·4) | 26·0 (30·5) | 27·1 (29·4) | 25·5 (31·2) | 17·6 (27·0) | 20·0 (23·6) |
|  | Systemic | 29·8 (24·6) | 15·7 (17·1) | 14·3 (21·5) | 10·3 (16·0) | 18·2 (17·4) | 19·0 (17·8) | 9·5 (25·2) |
|  | BSC | 36·8 (36·7) | 41·7 (50·0) | ·· | ·· | ·· | ·· | ·· |
| Appetite loss | All patients | 20·7 (30·9) | 24·2 (31·2) | 27·9 (32·9) | 23·2 (29·6) | 19·4 (28·4) | 18·9 (29·6) | 15·5 (24·9) |
|  | Surgery | 17·8 (31·4) | 16·5 (25·2) | 18·3 (26·2) | 22·1 (29·9) | 19·0 (30·9) | 12·1 (25·0) | 11·1 (19·5) |
|  | SRT | 16·7 (27·6) | 20·8 (28·8) | 21·4 (27·7) | 15·4 (24·3) | 15·8 (26·4) | 21·3 (31·3) | 17·0 (27·5) |
|  | WBRT | 22·3 (31·1) | 32·7 (35·1) | 44·0 (37·6) | 39·1 (32·6) | 29·8 (28·9) | 22·8 (31·1) | 20·0 (25·5) |
|  | Systemic | 33·3 (33·3) | 15·7 (29·1) | 21·4 (33·6) | 12·8 (21·7) | 9·1 (15·6) | 14·3 (26·2) | 9·5 (25·2) |
|  | BSC | 50·9 (40·6) | 33·3 (27·2) | ·· | ·· | ·· | ·· | ·· |
| Constipation | All patients | 25·3 (31·4) | 24·0 (30·7) | 22·9 (29·8) | 22·6 (27·1) | 20·5 (28·0) | 20·0 (26·1) | 22·0 (26·4) |
|  | Surgery | 15·4 (25·8) | 17·2 (24·3) | 18·6 (24·8) | 15·0 (20·6) | 17·5 (23·8) | 15·9 (23·3) | 17·6 (27·0) |
|  | SRT | 23·3 (29·5) | 21·8 (30·4) | 19·3 (27·7) | 22·2 (26·5) | 17·9 (28·9) | 21·3 (28·8) | 20·7 (26·4) |
|  | WBRT | 29·9 (33·3)* | 30·9 (34·4) | 32·5 (34·7) | 32·0 (31·6) | 29·8 (31·3) | 21·6 (23·9) | 34·7 (24·5) |
|  | Systemic | 14·0 (25·6) | 17·6 (20·8) | 7·1 (14·2) | 17·9 (17·3) | 21·2 (22·5) | 23·8 (25·2) | 9·5 (16·3) |
|  | BSC | 49·1 (37·5) | 25·0 (16·7) | ·· | ·· | ·· | ·· | ·· |
| Headaches | All patients | 18·8 (26·7) | 18·0 (23·9) | 17·6 (23·8) | 18·5 (23·7) | 16·0 (24·9) | 15·2 (22·3) | 13·0 (21·7) |
|  | Surgery | 21·9 (29·8) | 19·7 (24·7) | 19·9 (26·5) | 23·1 (27·0) | 17·2 (27·5) | 12·9 (23·0) | 15·7 (24·5) |
|  | SRT | 15·7 (23·2) | 19·5 (25·0) | 15·0 (21·5) | 15·1 (23·0) | 14·8 (24·5) | 16·4 (22·2) | 12·6 (19·6) |
|  | WBRT | 19·8 (27·8) | 15·8 (23·0) | 18·6 (24·4) | 20·1 (21·3) | 16·7 (23·0) | 14·0 (21·4) | 12·0 (21·3) |
|  | Systemic | 15·8 (20·4) | 15·7 (17·1) | 19·0 (21·5) | 15·4 (17·3) | 18·2 (22·9) | 23·8 (25·2) | 4·8 (12·6) |
|  | BSC | 24·6 (33·0) | 8·3 (16·7) | ·· | ·· | ·· | ·· | ·· |
| Visual disorders | All patients | 14·2 (21·5) | 13·3 (19·8) | 13·1 (19·6) | 12·1 (19·2) | 10·3 (17·0) | 12·4 (20·8) | 8·7 (14·1) |
|  | Surgery | 11·8 (19·0) | 9·8 (18·7) | 11·7 (19·3) | 10·8 (17·4) | 9·7 (15·8) | 6·9 (11·6) | 9·0 (17·0) |
|  | SRT | 12·4 (19·8) | 12·5 (18·8) | 12·4 (18·3) | 10·3 (18·1) | 10·3 (17·8) | 11·9 (18·7) | 7·3 (12·8) |
|  | WBRT | 17·0 (23·3) | 17·2 (21·4) | 16·7 (21·8) | 17·6 (22·9) | 12·6 (17·9) | 20·5 (30·0) | 13·1 (13·5) |
|  | Systemic | 14·6 (23·7) | 6·5 (15·2) | 2·4 (8·9) | 6·0 (13·3) | 4·0 (10·3) | 9·5 (16·3) | 3·2 (5·4) |
|  | BSC | 6·4 (18·3) | 5·6 (11·1) | ·· | ·· | ·· | ·· | ·· |
| Seizures | All patients | 5·2 (16·1) | 6·7 (18·6) | 4·7 (14·4) | 4·5 (13·3) | 5·8 (16·2) | 4·7 (13·8) | 3·2 (10·7) |
|  | Surgery | 2·5 (9·8) | 1·6 (8·6) | 1·4 (6·8) | 2·1 (8·2) | 4·8 (14·5) | 2·3 (8·6) | 1·9 (7·7) |
|  | SRT | 5·8 (17·7) | 8·8 (20·8) | 5·6 (15·3) | 6·5 (16·3) | 6·9 (18·0) | 6·7 (17·3) | 3·4 (12·0) |
|  | WBRT | 6·2 (17·5) | 8·1 (20·7) | 6·7 (17·7) | 3·7 (11·9) | 4·3 (13·2) | 3·5 (10·4) | 5·3 (12·5) |
|  | Systemic | 3·5 (10·5) | 3·9 (11·1) | 2·4 (8·9) | 5·6 (13·0) | 9·1 (21·6) | 4·8 (12·6) | 0 (0) |
|  | BSC | 0 (0) | 16·7 (33·3) | ·· | ·· | ·· | ·· | ·· |
| Motor dysfunction | All patients | 27·8 (25·4) | 24·9 (24·2) | 24·9 (26·0) | 22·7 (25·2) | 21·3 (24·1) | 19·0 (19·9) | 15·3 (16·8) |
|  | Surgery | 21·8 (22·8) | 18·8 (21·4) | 18·8 (18·6) | 17·9 (21·2) | 21·5 (24·2) | 15·2 (18·9) | 12·3 (15·4) |
|  | SRT | 26·9 (26·5) | 21·9 (21·8) | 21·6 (23·5) | 21·8 (26·9) | 21·4 (26·4) | 19·1 (21·5) | 17·2 (19·7) |
|  | WBRT | 31·0 (25·1) | 32·8 (26·1) | 35·3 (30·9) | 31·7 (25·3) | 23·4 (20·5) | 23·7 (17·9) | 17·3 (11·6) |
|  | Systemic | 10·5 (12·0) | 9·8 (18·4) | 6·3 (11·3) | 6·8 (12·5) | 10·1 (11·6) | 14·3 (15·3) | 6·3 (10·8) |
|  | BSC | 43·3 (25·1) | 41·7 (31·9) | ·· | ·· | ·· | ·· | ·· |
| Communication deficit | All patients | 14·8 (20·7) | 12·2 (19·3) | 12·8 (20·6) | 12·4 (20·7) | 11·0 (19·1) | 9·1 (15·2) | 8·6 (15·3) |
|  | Surgery | 11·8 (18·2) | 8·2 (15·6) | 9·6 (15·1) | 9·1 (14·8) | 13·2 (22·9) | 9·3 (15·7) | 9·6 (14·3) |
|  | SRT | 13·2 (19·9) | 10·2 (17·9) | 10·9 (19·7) | 10·0 (20·0) | 10·9 (18·0) | 7·3 (14·2) | 9·4 (17·0) |
|  | WBRT | 17·8 (22·1) | 17·4 (22·3) | 18·8 (24·6) | 21·8 (25·8) | 10·2 (17·5) | 13·2 (16·1) | 7·6 (14·2) |
|  | Systemic | 5·3 (10·1) | 5·9 (11·2) | 2·4 (4·7) | 1·7 (4·2) | 2·0 (4·5) | 6·3 (16·8) | 0 (0) |
|  | BSC | 17·0 (25·2) | 8·3 (16·7) | ·· | ·· | ·· | ·· | ·· |
| Drowsiness | All patients | 35·4 (27·9) | 41·4 (27·7) | 40·9 (28·2) | 35·2 (26·6) | 33·6 (26·0) | 29·7 (26·6) | 25·6 (22·5) |
|  | Surgery | 28·6 (27·1) | 35·3 (24·3) | 31·9 (22·4) | 33·8 (23·1) | 31·7 (24·7) | 25·0 (26·0) | 22·9 (22·5) |
|  | SRT | 34·6 (27·6) | 37·8 (26·5) | 36·3 (25·1) | 30·1 (25·3) | 33·7 (27·1) | 32·0 (26·0) | 27·0 (22·9) |
|  | WBRT | 38·4 (28·1) | 50·0 (28·1) | 54·2 (30·6) | 48·9 (27·3) | 39·7 (24·7) | 33·3 (29·0) | 30·7 (21·3) |
|  | Systemic | 29·8 (24·6) | 27·5 (29·4) | 30·8 (31·8) | 15·4 (25·9) | 18·2 (22·9) | 14·3 (17·8) | 9·5 (16·3) |
|  | BSC | 47·4 (27·9) | 50·0 (43·0) | ·· | ·· | ·· | ·· | ·· |
| Weakness of legs | All patients | 27·3 (31·5) | 34·9 (33·2) | 33·0 (32·9) | 30·2 (32·3) | 26·0 (28·3) | 23·2 (28·4) | 16·3 (21·8) |
|  | Surgery | 17·5 (27·5) | 24·5 (28·9) | 22·2 (27·1) | 23·1 (30·1) | 26·5 (30·0) | 17·5 (28·7) | 13·0 (18·3) |
|  | SRT | 27·6 (30·9) | 32·5 (32·1) | 39·9 (29·7) | 27·7 (30·6) | 25·1 (27·7) | 26·1 (27·7) | 17·0 (21·9) |
|  | WBRT | 29·8 (31·7) | 45·0 (33·9) | 46·7 (36·5) | 45·7 (34·0) | 29·0 (27·8) | 25·4 (29·4) | 22·7 (26·7) |
|  | Systemic | 19·3 (30·1) | 20·8 (34·2) | 14·3 (21·5) | 7·7 (14·6) | 18·2 (27·3) | 14·3 (26·2) | 4·8 (12·6) |
|  | BSC | 52·6 (40·5) | 50·0 (43·0) | ·· | ·· | ·· | ·· | ·· |

Scores (SD) for all responders at each assessment. Note: Higher scores for overall quality of life and functioning indicate better quality of life and function, higher scores for symptoms indicate higher symptom intensity. QoL: Quality of life. SRT: Stereotactic radiotherapy; WBRT: Whole brain radiotherapy; BSC: Best supportive care.

**Supplementary Table 7. Number of study participants and PRO responders at each assessment (attrition)**

|  | **Inclusion** | **Month 1** | **Month 2** | **Month 3** | **Month 6** | **Month 9** | **Month 12** |
| --- | --- | --- | --- | --- | --- | --- | --- |
| Number of study participants alive | 912 | 867 | 754 | 641 | 454 | 353 | 283 |
| Number of PRO responders alive | 740 | 715 | 642 | 553 | 393 | 315 | 260 |
| Total number of PRO responders | 708 | 445 | 374 | 291 | 218 | 164 | 126 |

PROs: Patient-reported outcomes**Supplementary Table 8. Change in patient-reported outcomes from inclusion to month 2 for complete responders at M0-M2, by ECOG-status at inclusion, surgery group.**

| **EORTC Scale** | **ECOG** | **Inclusion** | **Month 1** | **Month 2** | **Mean change (CI)**  **Inclusion vs. Month 2** | **≥ 10 points**  **change** |
| --- | --- | --- | --- | --- | --- | --- |
|  | | **Mean (SD)** | | |  |  |
| **Overall QoL** | ECOG 0 | 71·7 (24·8) | 76·7 (20·5) | 62·5 (27·5) | -9·2 (-22·9, 4·6) |  |
|  | ECOG 1 | 67·5 (22·6) | 61·8 (22·9) | 62·7 (23·1) | -4·8 (-13·5, 3·8) |  |
|  | ECOG 2 | 51·3 (23·0) | 64·1 (16·5) | 62·8 (22·7) | 11·5 (-6·6, 29·6) | X |
|  | ECOG 3-4 | 40·0 (27·9) | 43·3 (19·0) | 53·3 (24·7) | 13·3 (-36·1, 62·7) | X |
| **Physical function** | ECOG 0 | 88·4 (16·3) | 86·7 (19·9) | 86·2 (12·1) | -2·1 (-8·6, 4·4) |  |
|  | ECOG 1 | 83·9 (13·1) | 80·1 (14·6) | 77·8 (19·0) | -6·1 (-12·5, 0·3) |  |
|  | ECOG 2 | 65·9 (24·1) | 68·8 (24·6) | 76·1 (23·5) | 10·3 (-8·6, 29·1) | X |
|  | ECOG 3-4 | 22·2 (15·7) | 44·4 (36·0) | 55·6 (35·1) | 33·3 (-13·5, 80·1) | X |
| **Emotional function** | ECOG 0 | 78·6 (22·4) | 87·5 (21·5) | 84·1 (22·0) | 5·6 (-1·4, 12·5) |  |
|  | ECOG 1 | 84·6 (20·6) | 80·7 (24·4) | 86·0 (15·8) | 1·3 (-5·3, 8·0) |  |
|  | ECOG 2 | 75·6 (25·1) | 80·8 (20·2) | 79·5 (24·7) | 3·8 (-17·2, 24·9) |  |
|  | ECOG 3-4 | 66·7 (20·4) | 66·7 (26·4) | 70·0 (27·4) | 3·3 (-23·6, 30·3) |  |
| **Fatigue** | ECOG 0 | 25·4 (22·7) | 27·5 (19·7) | 32·5 (25·0) | 7·1 (-5·7, 20·0) |  |
|  | ECOG 1 | 32·9 (22·4) | 41·7 (24·7) | 40·8 (25·3) | 7·9 (-0·3, 16·1) |  |
|  | ECOG 2 | 52·6 (25·3) | 42·3 (17·5) | 47·4 (19·1) | -5·1 (-19·6, 9·3) |  |
|  | ECOG 3-4 | 46·7 (36·1) | 46·7 (29·8) | 23·3 (19·0) | -23·3 (-66·2, 19·6,) | X |
| **Nausea/ vomiting** | ECOG 0 | 11·1 (19·2) | 10·0 (19·0) | 11·1 (19·2) | 0·0 (-6·8, 6·8) |  |
|  | ECOG 1 | 4·4 (11·4) | 10·5 (20·7) | 12·3 (22·5) | 8·9 (0·5, 15·3) |  |
|  | ECOG 2 | 12·8 (25·6) | 5·1 (18·5) | 12·8 (25·6) | 0·0 (-16·4, 16·4) |  |
|  | ECOG 3-4 | 46·7 (44·7) | 13·3 (18·3) | 6·7 (14·9) | -40·0 (-94·0, 14·0) | X |
| **Pain** | ECOG 0 | 17·5 (23·3) | 16·7 (24·2) | 23·8 (26·1) | 6·3 (-4·5, 17·2) |  |
|  | ECOG 1 | 21·1 (22·8) | 27·2 (30·9) | 21·9 (24·8) | 0·9 (-7·8, 9·5) |  |
|  | ECOG 2 | 29·5 (33·4) | 20·5 (18·2) | 23·1 (33·7) | -6·4 (-32·6, 19·8) |  |
|  | ECOG 3-4 | 23·3 (27·9) | 13·3 (21·7) | 20·0 (29·8) | -3·3 (-26·0, 19·3) |  |
| **Dyspnea** | ECOG 0 | 23·8 (23·9) | 21·7 (29·2) | 25·4 (29·6) | 1·6 (-7·4, 10·5) |  |
|  | ECOG 1 | 18·4 (28·7) | 19·8 (28·8) | 20·2 (25·2) | 1·8 (-7·4, 10·9) |  |
|  | ECOG 2 | 17·9 (32·2) | 28·2 (32·9) | 20·5 (25·6) | 2·6 (-12·7, 17·9) |  |
|  | ECOG 3-4 | 8·3 (16·7) | 13·3 (18·3) | 16·7 (33·3) | 8·3 (-58·4, 75·1) |  |
| **Sleep disturbance** | ECOG 0 | 34·9 (35·7) | 20·0 (22·7) | 27·0 (30·9) | -7·9 (-19·6, 3·7) |  |
|  | ECOG 1 | 36·8 (33·6) | 31·6 (31·9) | 24·6 (29·7) | -12·3 (-25·6, 1·1) | X |
|  | ECOG 2 | 48·7 (35·0) | 17·9 (25·9) | 17·9 (22·0) | -30·8 (-53·2, -8·3) | X |
|  | ECOG 3-4 | 60·0 (36·5) | 46·7 (29·8) | 33·3 (33·3) | -26·7 (-88·1, 34·7) | X |
| **Appetite loss** | ECOG 0 | 6·3 (17·1) | 6·7 (20·5) | 12·7 (22·3) | 6·3 (-4·0, 16·7) |  |
|  | ECOG 1 | 5·4 (14·7) | 13·2 (21·3) | 17·1 (24·4) | 11·7 (-2·9, -20·5) | X |
|  | ECOG 2 | 23·1 (34·4) | 20·5 (25·6) | 28·2 (32·9) | 5·1 (-17·9, 28·2) |  |
|  | ECOG 3-4 | 46·7 (29·8) | 46·7 (29·8) | 33·3 (40·8) | -13·3 (-60·5, 33·9) | X |
| **Constipation** | ECOG 0 | 6·3 (17·1) | 5·0 (12·2) | 9·5 (18·7) | 3·2 (-9·4, 15·8) |  |
|  | ECOG 1 | 18·4 (25·3) | 22·8 (27·0) | 21·1 (28·4) | 2·6 (-9·4, 14·7) |  |
|  | ECOG 2 | 18·4 (25·3) | 7·7 (14·6) | 21·1 (28·4) | 2·6 (-17·9, 12·7) |  |
|  | ECOG 3-4 | 40·0 (43·5) | 46·7 (44·7) | 33·3 (23·6) | -6·7 (-52·0, 38·7) |  |
| **Headaches** | ECOG 0 | 15·9 (22·7) | 18·3 (22·9) | 20·6 (30·7) | 4·8 (-9·9, 19·4) |  |
|  | ECOG 1 | 19·3 (22·8) | 22·5 (27·3) | 19·3 (27·5) | 0·0 (-8·8, 8·8) |  |
|  | ECOG 2 | 38·1 (41·0) | 21·4 (21·1) | 19·0 (28·4) | -19·0 (-49·0, 10·9) | X |
|  | ECOG 3-4 | 40·0 (43·5) | 20·0 (44·7) | 13·3 (18·3) | -26·7 (-61·3, 8·0) | X |
| **Visual disorders** | ECOG 0 | 10·1 (20·2) | 7·9 (17·3) | 9·0 (17·4) | -1·1 (-12·2, 10·1) |  |
|  | ECOG 1 | 13·0 (19·4) | 12·9 (20·4) | 13·5 (23·1) | 0·4 (-5·3, 6·1) |  |
|  | ECOG 2 | 11·1 (21·8) | 15·9 (24·9) | 15·1 (18·3) | 4·0 (-8·5, 16·5) |  |
|  | ECOG 3-4 | 2·2 (5·0) | 4·4 (9·9) | 4·4 (9·9) | 2·2 (-3·9, 8·4) |  |
| **Seizures** | ECOG 0 | 1·6 (7·3) | 3·2 (14·5) | 3·2 (10·0) | 1·6 (-1·7, 4·9) |  |
|  | ECOG 1 | 1·8 (7·5) | 1·8 (7·6) | 0·9 (5·4) | -0·9 (-4·0, 2·2) |  |
|  | ECOG 2 | 2·4 (8·9) | 0·0 (0·0) | 0·0 (0·0) | -2·4 (-7·5, 2·8) |  |
|  | ECOG 3-4 | 6·7 (14·9) | 0·0 (0·0) | 0·0 (0·0) | -6·7 (-25·2, 11·8) |  |
| **Motor dysfunction** | ECOG 0 | 10·6 (14·7) | 12·7 (16·2) | 13·2 (14·7) | 2·6 (-3·7, 9·0) |  |
|  | ECOG 1 | 16·4 (16·3) | 17·1 (19·9) | 17·5 (17·6) | 1·2 (-4·4, 6·8) |  |
|  | ECOG 2 | 33·3 (27·6) | 28·6 (20·3) | 28·6 (20·8) | -4·8 (-19·2, 9·6) |  |
|  | ECOG 3-4 | 53·3 (31·8) | 42·2 (33·7) | 28·9 (31·0) | -24·4 (-54·4, 5·5) | X |
| **Communication deficit** | ECOG 0 | 4·2 (7·4) | 3·2 (8·7) | 4·8 (10·3) | 0·5 (-3·8, 4·9) |  |
|  | ECOG 1 | 11·1 (14·8) | 9·3 (14·2) | 9·4 (15·0) | -1·8 (-6·5, 3·0) |  |
|  | ECOG 2 | 12·7 (13·7) | 13·5 (17·5) | 11·9 (12·7) | -0·8 (-8·5, 6·9) |  |
|  | ECOG 3-4 | 13·3 (18·3) | 4·4 (6·1) | 6·7 (9·9) | -6·7 (-19·0, 5·7) |  |
| **Drowsiness** | ECOG 0 | 19·0 (22·5) | 25·4 (20·8) | 25·4 (20·8) | 6·3 (-6·0, 18·7) |  |
|  | ECOG 1 | 24·6 (22·8) | 37·8 (23·8) | 31·6 (21·8) | 7·0 (-1·1, 15·1) |  |
|  | ECOG 2 | 35·7 (24·3) | 40·5 (23·3) | 28·6 (17·8) | -7·1 (-20·6, 6·3) |  |
|  | ECOG 3-4 | 40·0 (27·9) | 33·3 (33·3) | 26·7 (14·9) | -13·3 (-36·0, 9·3) | X |
| **Weakness of legs** | ECOG 0 | 11·7 (24·8) | 12·7 (22·3) | 16·7 (22·9) | 5·0 (-8·7, 18·7) |  |
|  | ECOG 1 | 13·2 (19·8) | 25·2 (28·8) | 23·7 (27·8) | 10·5 (1·7, 19·4) | X |
|  | ECOG 2 | 23·8 (27·5) | 40·5 (29·8) | 19·0 (31·3) | -4·8 (-32·9, 23·3) |  |
|  | ECOG 3-4 | 46·7 (50·6) | 40·0 (36·5) | 33·3 (33·3) | -13·3 (-88·5, 61·9) | X |

Number of responders: ECOG 0: 21; ECOG 38: 51; ECOG 2: 14; ECOG 3-4: 5. Note: Higher scores for overall quality of life and functioning indicate better quality of life and function, higher scores for symptoms indicate higher symptom intensity. “X” indicates clinically important change in scores (≥10 points) from inclusion to month 2. QoL: Quality of life.

**Supplementary Table 9. Changes in patient-reported outcomes from inclusion to month 2 for complete responders at M0-M2, by ECOG-status at inclusion, SRT group.**

| **EORTC Scale** | **ECOG** | **Inclusion** | **Month 1** | **Month 2** | **Mean change (CI)**  **Inclusion vs. Month 2** | **≥ 10 points**  **change** |
| --- | --- | --- | --- | --- | --- | --- |
|  | | **Mean (SD)** | | |  |  |
| **Overall QoL** | ECOG 0 | 70·7 (17·3) | 69·3 (16·2) | 66·7 (19·1) | -4·5 (-10·9, 1·9) |  |
|  | ECOG 1 | 59·8 (23·8) | 59·2 (23·3) | 56·7 (24·7) | -2·3 (-9·4, 4·8) |  |
|  | ECOG 2 | 46·8 (22·1) | 48·3 (23·5) | 50·0 (27·4) | 3·2 (-8·0, 14·3) |  |
|  | ECOG 3-4 | 41·7 (19·6) | 53·3 (24·6) | 35·0 (20·0) | -6·7 (-27·1, 13·8) |  |
| **Physical function** | ECOG 0 | 90·1 (12·1) | 88·9 (12·2) | 84·9 (17·9) | -6·2 (-12·3, 0·0) |  |
|  | ECOG 1 | 81·6 (18·4) | 75·3 (19·8) | 74·4 (22·9) | -7·0 (-13·8, -0·2) |  |
|  | ECOG 2 | 60·3 (24·5) | 63·8 (23·1) | 51·3 (31·5) | -9·0 (-23·5, 5·5) |  |
|  | ECOG 3-4 | 25·6 (21·0) | 48·9 (25·2) | 44·4 (24·0) | 18·9 (-2·0, 39·8) | X |
| **Emotional function** | ECOG 0 | 86·0 (17·4) | 89·7 (16·0) | 85·5 (19·2) | -0·5 (-5·9, 5·0) |  |
|  | ECOG 1 | 74·2 (26·3) | 80·3 (23·0) | 79·7 (25·7) | 6·0 (-0·6, 12·6) |  |
|  | ECOG 2 | 73·8 (22·7) | 77·0 (27·1) | 71·4 (30·3) | -2·4 (-9·3, 4·5) |  |
|  | ECOG 3-4 | 68·3 (27·7) | 66·7 (30·4) | 66·7 (24·8) | -1·7 (-8·4, 5·1) |  |
| **Fatigue** | ECOG 0 | 27·0 (19·8) | 33·3 (25·1) | 33·3 (26·8) | 7·7 (-0·5, 15·8) |  |
|  | ECOG 1 | 42·5 (23·9) | 44·9 (26·6) | 43·8 (24·3) | 1·3 (-6·1, 8·7) |  |
|  | ECOG 2 | 52·4 (27·5) | 50·0 (24·7) | 53·2 (28·7) | 0·8 (-12·9, 14·5) |  |
|  | ECOG 3-4 | 35·0 (26·6) | 58·3 (31·7) | 60·0 (19·6) | 25·0 (3·1, 46·9) | X |
| **Nausea/ vomiting** | ECOG 0 | 6·3 (15·4) | 11·1 (20·7) | 13·7 (22·6) | 7·2 (2·6, 11·8) |  |
|  | ECOG 1 | 10·0 (19·3) | 8·8 (17·7) | 13·1 (25·0) | 1·3 (-5·0, 7·7) |  |
|  | ECOG 2 | 12·7 (26·8) | 8·3 (21·3) | 11·1 (21·9) | -1·6 (-12·8, 9·6) |  |
|  | ECOG 3-4 | 13·3 (17·2) | 16·7 (23·6) | 16·7 (17·6) | 3·3 (-10·2, 16·9) |  |
| **Pain** | ECOG 0 | 15·8 (18·4) | 20·1 (21·4) | 22·2 (22·4) | 7·7 (1·4, 13·9) |  |
|  | ECOG 1 | 19·0 (27·7) | 24·5 (28·3) | 22·5 (28·1) | 3·6 (-3·7, 10·9) |  |
|  | ECOG 2 | 28·6 (32·6) | 19·8 (29·6) | 19·8 (27·2) | -8·7 (-21·8, 4·3) |  |
|  | ECOG 3-4 | 38·3 (28·4) | 38·3 (33·4) | 56·7 (35·3) | 18·3 (-3·8, 40·4) | X |
| **Dyspnea** | ECOG 0 | 16·2 (24·4) | 21·4 (23·6) | 20·5 (26·1) | 4·5 (-2·0, 11·0) |  |
|  | ECOG 1 | 24·7 (21·1) | 29·9 (27·7) | 24·8 (24·8) | 0·0 (-6·1, 6·1) |  |
|  | ECOG 2 | 31·7 (34·1) | 28·6 (28·5) | 39·7 (37·4) | 7·9 (-4·7, 20·5) |  |
|  | ECOG 3-4 | 16·7 (36·0) | 26·7 (34·4) | 30·0 (33·1) | 13·3 (-12·3, 39·0) | X |
| **Sleep disturbance** | ECOG 0 | 22·5 (20·9) | 17·9 (22·7) | 18·8 (23·9) | -3·6 (-10·4, 3·2) |  |
|  | ECOG 1 | 30·1 (30·7) | 27·8 (27·8) | 21·6 (22·9) | -8·5 (-16·4, -0·6) |  |
|  | ECOG 2 | 39·7 (34·3) | 30·2 (29·6) | 27·0 (27·1) | -12·7 (-30·3, 4·9) | X |
|  | ECOG 3-4 | 40·0 (30·6) | 43·3 (31·6) | 33·3 (27·2) | -6·7 (-28·6, 15·2) |  |
| **Appetite loss** | ECOG 0 | 2·7 (9·2) | 12·0 (19·5) | 16·2 (24·0) | 12·6 (5·0, 20·2) | X |
|  | ECOG 1 | 16·0 (28·0) | 18·4 (26·4) | 21·6 (28·1) | 6·0 (-3·3, 15·3) |  |
|  | ECOG 2 | 25·4 (33·2) | 25·4 (34·8) | 28·6 (32·1) | 3·2 (-12·7, 19·0) |  |
|  | ECOG 3-4 | 20·0 (17·2) | 16·7 (23·6) | 26·7 (34·4) | 6·7 (-12·1, 25·5) |  |
| **Constipation** | ECOG 0 | 14·4 (25·5) | 14·5 (29·4) | 12·8 (22·4) | -1·8 (-9·6, 6·0) |  |
|  | ECOG 1 | 24·0 (27·8) | 20·8 (31·2) | 20·3 (29·1) | -3·3 (-13·0, 6·3) |  |
|  | ECOG 2 | 22·2 (24·3) | 17·5 (22·7) | 20·6 (28·8) | -1·6 (-13·8, 10·6) |  |
|  | ECOG 3-4 | 53·3 (35·8) | 43·3 (38·7) | 40·0 (34·4) | -13·3 (-41·3, 14·7) | X |
| **Headaches** | ECOG 0 | 13·7 (19·8) | 24·8 (23·8) | 17·9 (21·4) | 4·3 (-3·2, 11·8) |  |
|  | ECOG 1 | 14·7 (26·2) | 14·4 (22·4) | 16·0 (26·3) | 1·3 (-5·6, 8·2) |  |
|  | ECOG 2 | 14·3 (19·9) | 22·2 (21·9) | 14·3 (16·9) | 0·0 (-9·6, 9·6) |  |
|  | ECOG 3-4 | 10·0 (16·1) | 20·0 (32·2) | 10·0 (16·1) | 0·0 (-15·9, 15·9) |  |
| **Visual disorders** | ECOG 0 | 6·0 (14·8) | 8·8 (16·9) | 6·3 (11·3) | 0·3 (-4·7, 5·3) |  |
|  | ECOG 1 | 12·9 (22·1) | 11·8 (19·9) | 15·1 (22·7) | 2·1 (-3·8, 8·0) |  |
|  | ECOG 2 | 24·3 (21·6) | 13·8 (15·7) | 14·8 (16·6) | -9·5 (-18·3, -0·7) |  |
|  | ECOG 3-4 | 22·2 (25·7) | 25·6 (25·7) | 20·0 (18·7) | -2·2 (-16·6, 12·2) |  |
| **Seizures** | ECOG 0 | 2·6 (11·8) | 3·4 (10·2) | 0·9 (5·3) | -1·7 (-5·2, 1·8) |  |
|  | ECOG 1 | 4·2 (16·3) | 10·0 (21·6) | 7·3 (18·2) | 3·5 (-3·3, 10·4) |  |
|  | ECOG 2 | 5·0 (12·2) | 17·5 (27·1) | 11·1 (19·2) | 5·0 (-5·5, 15·5) |  |
|  | ECOG 3-4 | 6·7 (21·1) | 10·0 (22·5) | 3·3 (10·5) | -3·3 (-10·9, 4·2) |  |
| **Motor dysfunction** | ECOG 0 | 7·0 (13·7) | 8·8 (12·5) | 10·0 (14·6) | 3·0 (-2·6, 8·6) |  |
|  | ECOG 1 | 21·1 (23·2) | 20·7 (19·9) | 19·4 (23·2) | -1·6 (-7·3, 4·0) |  |
|  | ECOG 2 | 33·3 (21·9) | 31·7 (19·3) | 40·5 (27·5) | 7·1 (-5·2, 19·5) |  |
|  | ECOG 3-4 | 68·9 (23·9) | 43·3 (24·3) | 43·3 (25·9) | -25·6 (-47·1, -4·0) | X |
| **Communication deficit** | ECOG 0 | 7·9 (15·3) | 6·6 (12·9) | 4·8 (11·3) | -2·9 (-6·4, 0·5) |  |
|  | ECOG 1 | 13·0 (21·7) | 9·6 (15·4) | 11·5 (20·5) | -1·4 (-5·7, 2·9) |  |
|  | ECOG 2 | 27·5 (29·1) | 16·9 (29·1) | 18·5 (28·8) | -9·0 (-24·0, 6·0) |  |
|  | ECOG 3-4 | 17·8 (27·3) | 13·3 (20·8) | 18·9 (25·7) | 1·1 (-17·4, 19·6) |  |
| **Drowsiness** | ECOG 0 | 20·7 (21·3) | 26·5 (25·6) | 28·2 (22·3) | 8·1 (1·0, 15·2) |  |
|  | ECOG 1 | 30·7 (25·9) | 37·3 (25·8) | 35·3 (23·5) | 4·0 (-4·0, 12·0) |  |
|  | ECOG 2 | 50·8 (32·7) | 41·3 (20·8) | 42·9 (28·2) | -7·9 (-21·4, 5·6) |  |
|  | ECOG 3-4 | 46·7 (28·1) | 56·7 (31·6) | 60·0 (26·3) | 13·3 (-9·7, 36·4) | X |
| **Weakness of legs** | ECOG 0 | 8·8 (16·8) | 17·1 (20·0) | 14·9 (22·9) | 6·3 (-1·5, 14·1) |  |
|  | ECOG 1 | 19·0 (24·5) | 34·7 (34·3) | 31·4 (30·1) | 12·2 (3·1, 21·3) | X |
|  | ECOG 2 | 40·0 (33·5) | 39·7 (32·7) | 42·9 (33·6) | 3·3 (-12·6, 19·3) |  |
|  | ECOG 3-4 | 40·0 (41·0) | 43·3 (22·5) | 43·3 (31·6) | 3·3 (-39·4, 46·1) |  |

Number of responders: ECOG 0: 39; ECOG 1: 51; ECOG 2: 21; ECOG 3-4: 10. Note: Higher scores for overall quality of life and functioning indicate better quality of life and function, higher scores for symptoms indicate higher symptom intensity. “X” indicates clinically meaningful change in scores (≥10 points) from inclusion to month 2. SRT: Stereotactic radiotherapy.QoL: Quality of life.

**Supplementary Table 10. Changes in patient-reported outcomes from inclusion to month 2 for complete responders at M0-M2, by ECOG-status at inclusion, WBRT group.**

| **EORTC Scale** | **ECOG** | **Inclusion** | **Month 1** | **Month 2** | **Mean change (CI)**  **Inclusion vs. Month 2** | **≥ 10 points**  **change** |
| --- | --- | --- | --- | --- | --- | --- |
|  | | **Mean (SD***)* | | |  |  |
| **Overall QoL** | ECOG 0 | 71·0 (18·3) | 60·1 (24·0) | 53·6 (22·4) | -17·4 (-27·9, -6·9) | X |
|  | ECOG 1 | 58·9 (24·0) | 55·0 (25·3) | 53·9 (20·9) | -5·0 (-12·5, 2·5) |  |
|  | ECOG 2 | 42·4 (22·3) | 44·9 (22·7) | 31·8 (26·6) | -10·6 (-25·3, 4·1) | X |
|  | ECOG 3-4 | 55·6 (17·2) | 44·4 (22·8) | 41·7 (27·4) | -13·9 (-55·9, 28·1) | X |
| **Physical function** | ECOG 0 | 93·2 (7·3) | 80·7 (20·2) | 72·5 (27·6) | -20·8 (-31·5, -10·0) | X |
|  | ECOG 1 | 75·9 (18·0) | 68·1 (25·8) | 65·7 (31·2) | -10·2 (-20·0, -0·4) | X |
|  | ECOG 2 | 62·6 (22·9) | 46·3 (30·5) | 28·8 (31·6) | -33·8 (-47·0, -20·6) | X |
|  | ECOG 3-4 | 37·0 (19·5) | 36·1 (30·2) | 29·6 (34·2) | -7·4 (-44·0, 29·2) |  |
| **Emotional function** | ECOG 0 | 84·8 (15·4) | 80·4 (26·4) | 82·6 (22·7) | -2·3 (-10·3, 5·7) |  |
|  | ECOG 1 | 77·0 (21·3) | 79·2 (24·9) | 81·2 (24·2) | 4·3 (-2·0, 10·5) |  |
|  | ECOG 2 | 73·2 (24·5) | 71·5 (27·6) | 58·7 (31·7) | 2·4 (-22·1, 22·1) | X |
|  | ECOG 3-4 | 72·2 (31·0) | 61·1 (47·9) | 72·2 (37·5) | 0·0 (-22·1, -22·1) |  |
| **Fatigue** | ECOG 0 | 19·6 (15·6) | 46·4 (27·0) | 52·2 (26·7) | 32·6 (21·0, 44·2) | X |
|  | ECOG 1 | 38·7 (20·0) | 49·7 (26·3) | 53·5 (28·0) | 14·9 (6·2, 23·6) | X |
|  | ECOG 2 | 50·0 (25·1) | 67·4 (25·8) | 76·8 (20·3) | 26·8 (15·6, 38·0) | X |
|  | ECOG 3-4 | 63·3 (39·8) | 69·4 (26·7) | 53·3 (29·8) | -10·0 (-41·4, 21·3) | X |
| **Nausea/ vomiting** | ECOG 0 | 9·1 (15·2) | 20·3 (26·1) | 28·8 (33·0) | 19·7 (4·8, 34·6) | X |
|  | ECOG 1 | 11·6 (22·5) | 24·3 (27·3) | 26·8 (29·5) | 15·2 (4·4, 26·0) | X |
|  | ECOG 2 | 14·5 (22·1) | 20·3 (29·7) | 39·1 (32·8) | 24·6 (11·4, 37·8) | X |
|  | ECOG 3-4 | 27·8 (32·8) | 33·3 (29·8) | 16·7 (18·3) | -11·1 (-39·7, 17·5) | X |
| **Pain** | ECOG 0 | 7·2 (12·1) | 23·9 (25·5) | 25·4 (30·1) | 18·1 (5·3, 31·0) | X |
|  | ECOG 1 | 19·1 (25·5) | 14·9 (20·11) | 22·0 (29·3) | 2·8 (-4·8, 10·5) |  |
|  | ECOG 2 | 21·0 (25·2) | 26·4 (32·2) | 39·1 (35·4) | 18·1 (1·7, 34·5) | X |
|  | ECOG 3-4 | 33·3 (27·9) | 33·3 (27·9) | 38·9 (31·0) | 5·6 (-32·2, 43·3) |  |
| **Dyspnea** | ECOG 0 | 7·2 (14·1) | 21·7 (25·8) | 20·3 (26·1) | 13·0 (2·6, 23·5) | X |
|  | ECOG 1 | 24·8 (28·6) | 31·3 (30·3) | 29·1 (29·2) | 4·3 (-2·7, 11·2) |  |
|  | ECOG 2 | 17·4 (28·2) | 37·5 (33·1) | 47·8 (34·6) | 30·4 (12·0, 48·8) | X |
|  | ECOG 3-4 | 33·3 (29·8) | 50·0 (35·0) | 50·0 (27·9) | 16·7 (-20·0, 53·4) | X |
| **Sleep disturbance** | ECOG 0 | 24·6 (25·1) | 34·8 (33·3) | 24·6 (32·1) | 0·0 (-14·4, 14·4) |  |
|  | ECOG 1 | 35·5 (27·6) | 35·4 (33·3) | 22·5 (29·9) | -13·0 (-22·9, -3·1) | X |
|  | ECOG 2 | 34·8 (35·5) | 33·3 (35·4) | 29·0 (32·3) | -5·8 (-21·9, 10·3) |  |
|  | ECOG 3-4 | 27·8 (25·1) | 27·8 (32·8) | 27·8 (32·8) | 0·0 (-22·1, 22·1) |  |
| **Appetite loss** | ECOG 0 | 11·6 (23·8) | 33·3 (33·3) | 49·3 (34·6) | 37·7 (23·1, 52·3) | X |
|  | ECOG 1 | 14·2 (25·8) | 22·9 (30·1) | 32·6 (34·4) | 18·4 (5·9, 31·0) | X |
|  | ECOG 2 | 17·4 (31·6) | 36·1 (36·7) | 68·1 (36·1) | 50·7 (31·4, 70·1) | X |
|  | ECOG 3-4 | 44·4 (40·4) | 33·3 (29·8) | 38·9 (32·8) | -5·6 (-46·5, 35·3) |  |
| **Constipation** | ECOG 0 | 30·4 (30·0) | 34·8 (34·1) | 34·8 (35·5) | 4·3 (-10·3, 19·0) |  |
|  | ECOG 1 | 19·6 (25·9) | 25·7 (30·2) | 31·2 (34·0) | 11·6 (0·5, 22·7) | X |
|  | ECOG 2 | 29·0 (36·7) | 25·0 (26·5) | 36·2 (36·1) | 7·2 (-11·2, 25·7) |  |
|  | ECOG 3-4 | 33·3 (42·2) | 50·0 (40·8) | 44·4 (40·4) | 11·1 (-25·0, 47·2) | X |
| **Headaches** | ECOG 0 | 14·5 (19·7) | 14·5 (22·1) | 20·3 (28·0) | 5·8 (-6·2, 17·8) |  |
|  | ECOG 1 | 18·1 (25·7) | 17·7 (21·8) | 18·8 (22·7) | 0·7 (-5·5, 6·8) |  |
|  | ECOG 2 | 17·4 (26·3) | 13·9 (21·8) | 18·8 (26·3) | 1·4 (-13·9, 16·8) |  |
|  | ECOG 3-4 | 27·8 (25·1) | 27·8 (32·8) | 22·2 (27·2) | -5·6 (-46·5, 35·3) |  |
| **Visual disorders** | ECOG 0 | 6·6 (14·4) | 12·8 (21·5) | 12·9 (20·1) | 6·3 (0·1, 12·5) |  |
|  | ECOG 1 | 13·5 (18·5) | 13·2 (18·9) | 14·2 (18·3) | 0·7 (-4·1, 5·5) |  |
|  | ECOG 2 | 14·8 (18·7) | 16·2 (17·7) | 23·8 (21·7) | 9·0 (-0·3, 18·3) |  |
|  | ECOG 3-4 | 24·1 (29·3) | 27·8 (39·0) | 27·8 (39·0) | 3·7 (-21·5, 28·9) |  |
| **Seizures** | ECOG 0 | 5·8 (16·4) | 11·6 (23·8) | 7·2 (17·3) | 1·4 (-9·6, 12·5) |  |
|  | ECOG 1 | 4·2 (13·1) | 7·1 (20·8) | 4·2 (13·1) | 0·0 (-4·0, 4·0) |  |
|  | ECOG 2 | 9·1 (21·0) | 11·6 (25·8) | 12·1 (28·3) | 3·0 (-8·1, 14·1) |  |
|  | ECOG 3-4 | 11·1 (27·2) | 5·6 (13·6) | 0·0 (0·0) | -11·1 (-39·7, 17·5) | X |
| **Motor dysfunction** | ECOG 0 | 7·2 (9·2) | 18·4 (24·8) | 26·1 (26·9) | 18·8 (8·9, 28·8) | X |
|  | ECOG 1 | 27·5 (23·5) | 29·8 (22·0) | 29·6 (28·1) | 2·1 (-7·6, 11·8) |  |
|  | ECOG 2 | 33·6 (24·4) | 44·2 (26·6) | 51·0 (32·0) | 17·4 (5·4, 29·4) | X |
|  | ECOG 3-4 | 31·5 (21·6) | 42·6 (36·8) | 42·6 (30·2) | 11·1 (-13·3, 35·6) | X |
| **Communication deficit** | ECOG 0 | 6·3 (13·3) | 11·1 (21·5) | 11·1 (17·1) | 4·8 (-1·6, 11·3) |  |
|  | ECOG 1 | 15·5 (17·4) | 14·2 (16·5) | 16·7 (20·6) | 1·2 (-4·8, 7·1) |  |
|  | ECOG 2 | 17·9 (29·2) | 21·5 (27·3) | 32·1 (30·1) | 14·3 (-1·8, 30·3) | X |
|  | ECOG 3-4 | 20·4 (28·5) | 35·2 (39·4) | 25·9 (28·7) | 5·6 (-23·7, 34·8) |  |
| **Drowsiness** | ECOG 0 | 15·9 (17·0) | 39·3 (23·9) | 50·7 (31·6) | 34·8 (20·1, 49·5) | X |
|  | ECOG 1 | 32·6 (20·0) | 44·7 (28·0) | 45·1 (26·2) | 12·5 (4·9, 20·1) | X |
|  | ECOG 2 | 43·5 (30·9) | 61·1 (28·9) | 75·4 (27·0) | 31·9 (12·7, 51·1) | X |
|  | ECOG 3-4 | 61·1 (39·0) | 66·7 (29·8) | 55·6 (34·4) | -5·6 (-46·5, 35·3) |  |
| **Weakness of legs** | ECOG 0 | 7·2 (14·1) | 26·1 (30·1) | 36·2 (33·2) | 29·0 (15·7, 42·2) | X |
|  | ECOG 1 | 23·6 (24·8) | 40·6 (32·9) | 38·2 (32·2) | 14·6 (5·0, 24·1) | X |
|  | ECOG 2 | 34·8 (35·5) | 59·7 (36·8) | 66·7 (37·6) | 31·9 (11·7, 52·0) | X |
|  | ECOG 3-4 | 44·4 (17·2) | 72·2 (25·1) | 61·1 (39·0) | 16·7 (-31·6, 64·9) | X |

Number of responders: ECOG 0: 23; ECOG 1: 48; ECOG 2: 24; ECOG 3-4: 6. Note: Higher scores for overall quality of life and functioning indicate better quality of life and function, higher scores for symptoms indicate higher symptom intensity. “X” indicates clinically meaningful change in scores (≥10 points) from inclusion to month 2. WBRT: Whole brain radiotherapy. QoL: Quality of life.

**Supplementary Table 11. Changes in patient-reported outcomes from inclusion to month 2 for complete responders at M0-M2 by survival groups according to survival after date of BM diagnosis.**

| **EORTC scale** | **Survival groups** | **Inclusion** | **Month 1** | **Month 2** | **Mean change (CI)**  **Inclusion vs. Month 2** | **≥ 10 points**  **change** |
| --- | --- | --- | --- | --- | --- | --- |
|  |  | Mean (SD) | | |  |  |
| **Overall quality of life** | Alive < 3 months | 55·6 (22·1) | 27·8 (19·2) | 30·6 (22·3) | -25·0 (-44·9, -5·1) | X |
|  | Alive 3-6 months | 56·2 (24·0) | 53·2 (23·0) | 43·3 (26·9) | -12·9 (-20·7, -5·1) | X |
|  | Alive 6-12 months | 57·4 (23·8) | 59·1 (23·6) | 56·7 (24·0) | -0·7 (-7·0, 5·6) |  |
|  | Alive > 12 months | 62·7 (23·9) | 63·6 (22·1) | 61·0 (21·9) | -1·6 (-5·3, 2·0) |  |
| **Physical function** | Alive < 3 months | 60·7 (29·6) | 25·2 (21·8) | 20·5 (19·7) | -40·2 (-58·7, -21·7) | X |
|  | Alive 3-6 months | 67·6 (26·5) | 58·8 (30·1) | 42·6 (33·0) | -25·0 (-33·9, -16·1) | X |
|  | Alive 6-12 months | 71·1 (24·1) | 75·4 (21·0) | 70·3 (25·5) | -0·8 (-7·4, 5·8) |  |
|  | Alive > 12 months | 81·6 (20·8) | 79·4 (18·9) | 80·6 (18·7) | -0·9 (-3·9, 2·1) |  |
| **Emotional function** | Alive < 3 months | 71·8 (27·5) | 52·6 (25·2) | 48·7 (30·0) | -23·1 (-39·3, -6·9) | X |
|  | Alive 3-6 months | 78·7 (22·1) | 78·6 (27·8) | 75·7 (27·0) | -3·0 (-9·3, 3·3) |  |
|  | Alive 6-12 months | 78·3 (21·9) | 81·2 (24·6) | 79·8 (26·0) | 1·4 (-3·9, 6·7) |  |
|  | Alive > 12 months | 78·3 (23·2) | 82·4 (21·7) | 83·1 (20·9) | 4·9 (1·7, 8·1) |  |
| **Fatigue** | Alive < 3 months | 47·4 (22·4) | 80·8 (22·4) | 84·6 (24·0) | 37·2 (15·4, 59·0) | X |
|  | Alive 3-6 months | 36·9 (25·5) | 53·2 (29·2) | 60·9 (27·9) | 24·0 (16·5, 31·5) | X |
|  | Alive 6-12 months | 40·7 (25·8) | 42·8 (23·3) | 43·1 (25·1) | 2·4 (-4·5, 9·3) |  |
|  | Alive > 12 months | 35·2 (24·1) | 40·7 (25·3) | 40·4 (24·4) | 5·3 (1·4, 9·1) |  |
| **Nausea / vomiting** | Alive < 3 months | 15·4 (25·9) | 20·5 (32·0) | 23·1 (34·4) | 7·7 (-17·2, 32·6) |  |
|  | Alive 3-6 months | 13·4 (23·0) | 17·2 (24·7) | 25·3 (31·2) | 11·8 (2·6, 21·0) | X |
|  | Alive 6-12 months | 8·2 (19·3) | 11·8 (21·4) | 14·0 (23·8) | 5·8 (0·0, 11·6) |  |
|  | Alive > 12 months | 10·0 (19·6) | 13·3 (22·3) | 16·0 (23·5) | 5·9 (2·3, 9·6) |  |
| **Pain** | Alive < 3 months | 46·2 (39·8) | 51·3 (46·9) | 53·8 (43·1) | 7·7 (-25·6, 41·0) |  |
|  | Alive 3-6 months | 18·5 (23·2) | 22·8 (24·7) | 36·3 (32·7) | 17·7 (9·8, 25·7) | X |
|  | Alive 6-12 months | 21·2 (26·5) | 19·3 (27·1) | 21·9 (27·6) | 0·7 (-4·4, 5·8) |  |
|  | Alive > 12 months | 19·1 (24·2) | 21·3 (23·9) | 21·3 (24·9) | 2·2 (-1·8, 6·2) |  |
| **Dyspnea** | Alive < 3 months | 15·4 (25·9) | 51·3 (35·0) | 59·0 (33·8) | 43·6 (19·8, 67·4) | X |
|  | Alive 3-6 months | 21·5 (24·2) | 31·7 (31·9) | 31·7 (31·6) | 10·2 (2·9, 17·5) |  |
|  | Alive 6-12 months | 25·1 (33·5) | 26·6 (31·1) | 29·0 (32·8) | 3·9 (-2·0, 9·8) |  |
|  | Alive > 12 months | 19·1 (24·5) | 23·2 (24·9) | 22·0 (24·3) | 2·8 (-0·9, 6·6) |  |
| **Sleep disturbance** | Alive < 3 months | 25·6 (36·4) | 30·8 (39·6) | 38·5 (42·7) | 12·8 (-8·2, 33·8) | X |
|  | Alive 3-6 months | 30·6 (31·2) | 32·3 (32·8) | 26·2 (31·7) | -4·4 (-13·5, 4·8) |  |
|  | Alive 6-12 months | 35·2 (27·7) | 31·9 (28·8) | 26·2 (26·6) | -9·0 (-17·0, -1·1) |  |
|  | Alive > 12 months | 34·3 (31·1) | 26·8 (28·8) | 20·4 (24·9) | -13·9 (-18·7, -9·2) | X |
| **Appetite loss** | Alive < 3 months | 25·6 (43·4) | 46·2 (42·0) | 43·6 (39·4) | 17·9 (-17·5, 53·4) | X |
|  | Alive 3-6 months | 16·1 (27·5) | 30·2 (32·6) | 48·4 (38·5) | 32·3 (20·4, 44·1) | X |
|  | Alive 6-12 months | 12·9 (22·9) | 16·9 (24·7) | 22·4 (28·2) | 9·5 (3·2, 15·9) |  |
|  | Alive > 12 months | 13·1 (24·7) | 15·7 (24·5) | 22·3 (28·7) | 9·2 (4·5, 13·9) |  |
| **Constipation** | Alive < 3 months | 43·6 (39·4) | 41·0 (30·9) | 46·2 (34·8) | 2·6 (-16·7, 21·8) |  |
|  | Alive 3-6 months | 28·0 (35·8) | 31·2 (33·3) | 41·4 (39·4) | 13·4 (1·0, 25·8) | X |
|  | Alive 6-12 months | 15·7 (21·0) | 18·8 (26·5) | 18·1 (25·8) | 2·4 (-4·0, 8·8) |  |
|  | Alive > 12 months | 20·3 (27·3) | 19·8 (29·0) | 17·0 (23·5) | -3·3 (-7·5, 1·0) |  |
| **Headaches** | Alive < 3 months | 10·3 (25·0) | 17·9 (22·0) | 17·9 (22·0) | 7·7 (-12·7, 28·1) |  |
|  | Alive 3-6 months | 16·1 (23·2) | 15·1 (24·6) | 19·4 (28·0) | 3·2 (-3·7, 10·2) |  |
|  | Alive 6-12 months | 15·5 (25·1) | 15·9 (22·6) | 16·4 (21·0) | 0·9 (-5·5, 7·3) |  |
|  | Alive > 12 months | 18·8 (25·5) | 21·2 (23·8) | 18·0 (24·7) | -0·8 (-5·1, 3·5) |  |
| **Visual disorder** | Alive < 3 months | 18·2 (24·0) | 13·6 (24·3) | 17·7 (26·9) | -0·5 (-11·9, 10·9) |  |
|  | Alive 3-6 months | 14·0 (21·7) | 15·6 (25·2) | 20·0 (24·6) | 6·0 (1·4, 10·6) |  |
|  | Alive 6-12 months | 11·6 (19·7) | 11·6 (16·8) | 13·2 (18·5) | 1·6 (-3·7, 6·9) |  |
|  | Alive > 12 months | 11·7 (18·9) | 11·5 (18·0) | 10·6 (17·3) | -1·1 (-3·6, 1·5) |  |
| **Seizures** | Alive < 3 months | 2·8 (9·6) | 8·3 (20·7) | 8·3 (20·7) | 5·6 (-9·6, 20·8) |  |
|  | Alive 3-6 months | 4·3 (14·1) | 7·5 (19·5) | 5·4 (18·3) | 1·1 (-3·8, 5·9) |  |
|  | Alive 6-12 months | 1·0 (5·6) | 2·9 (12·4) | 4·8 (15·4) | 3·9 (0·4, 7·4) |  |
|  | Alive > 12 months | 5·5 (16·2) | 8·3 (20·2) | 3·8 (11·9) | -1·6 (-4·2, 1·0) |  |
| **Motor dysfunction** | Alive < 3 months | 36·3 (31·5) | 55·1 (31·3) | 56·8 (34·4) | 20·5 (6·1, 34·9) | X |
|  | Alive 3-6 months | 26·0 (22·4) | 30·9 (24·6) | 38·4 (29·5) | 12·4 (3·8, 21·0) | X |
|  | Alive 6-12 months | 27·2 (26·1) | 26·3 (23·5) | 27·9 (24·5) | 0·7 (-5·0, 6·4) |  |
|  | Alive > 12 months | 18·0 (22·2) | 17·1 (18·9) | 16·5 (20·0) | -1·5 (-4·4, 1·5) |  |
| **Communication deficit** | Alive < 3 months | 20·5 (21·2) | 37·2 (25·0) | 45·7 (27·6) | 25·2 (10·9, 39·5) | X |
|  | Alive 3-6 months | 14·7 (22·0) | 15·8 (22·7) | 21·5 (24·9) | 6·8 (0·8, 12·8) |  |
|  | Alive 6-12 months | 14·1 (21·7) | 11·1 (19·7) | 12·1 (18·9) | -2·0 (-7·4, 3·3) |  |
|  | Alive > 12 months | 11·5 (18·8) | 8·1 (14·4) | 7·7 (14·5) | -3·8 (-6·2, -1·5) |  |
| **Drowsiness** | Alive < 3 months | 41·0 (33·8) | 76·9 (28·5) | 79·5 (25·6) | 38·5 (9·0, 67·9) | X |
|  | Alive 3-6 months | 33·3 (27·7) | 45·2 (30·2) | 56·5 (32·3) | 23·1 (13·8, 32·4) | X |
|  | Alive 6-12 months | 36·6 (27·7) | 39·5 (24·3) | 36·6 (25·3) | 0·0 (-6·9, 6·9) |  |
|  | Alive > 12 months | 26·6 (23·4) | 34·5 (24·5) | 32·5 (21·9) | 5·9 (2·3, 9·5) |  |
| **Weakness of legs** | Alive < 3 months | 38·5 (30·0) | 64·1 (37·2) | 74·4 (27·7) | 35·9 (12·0, 59·8) | X |
|  | Alive 3-6 months | 30·6 (30·4) | 45·7 (33·7) | 55·4 (33·0) | 24·7 (14·0, 35·5) | X |
|  | Alive 6-12 months | 22·4 (28·2) | 33·3 (31·8) | 30·0 (32·2) | 7·6 (-0·5, 15·8) |  |
|  | Alive > 12 months | 15·5 (24·6) | 26·5 (28·8) | 22·1 (26·8) | 6·5 (1·8, 11·3) |  |

Number of responders: Alive < 3 months: 13; Alive 3-6 months: 63; Alive 6-12 months: 71; Alive > 12 months: 168. Note: Higher scores for overall quality of life and functioning indicate better quality of life and function, higher scores for symptoms indicate higher symptom intensity. “X” indicates clinically meaningful change in scores (≥10 points) from inclusion to month 2. QoL: Quality of life.

**Supplementary Table 12. Change in patient-reported outcomes from inclusion to month 2 for complete responders at M0-M2, by survival groups according to survival after date of BM diagnosis, lung cancer group.**

| **EORTC Scale** | **Survival group** | **Inclusion** | **Month 1** | **Month 2** | **Mean change (CI)** | **≥ 10 points**  **change** |
| --- | --- | --- | --- | --- | --- | --- |
|  | | **Mean (SD)** | | |  |  |
| **Overall Quality of Life** | Alive < 3 months | 54·7 (18·5) | 33·3 (21·5) | 38·1 (24·9) | -16·7 (-47·5, 14·2) | X |
|  | Alive 3-6 months | 57·7 (26·6) | 52·4 (23·4) | 41·7 (25·9) | -16·1 (-28·6, -3·6) | X |
|  | Alive 6-12 months | 59·5 (25·6) | 60·3 (23·3) | 54·0 (23·5) | -5·6 (-16·9, 5·8) |  |
|  | Alive > 12 months | 59·4 (24·5) | 63·8 (21·3) | 60·9 (20·8) | 1·4 (-4·5, 7·4) |  |
| **Physical function** | Alive < 3 months | 69·4 (24·3) | 27·1 (23·7) | 23·6 (19·2) | -45·8 (-65·3, -26·3) | X |
|  | Alive 3-6 months | 70·8 (22·9) | 55·9 (27·6) | 41·2 (32·6) | -29·6 (-42·6, -16·6) | X |
|  | Alive 6-12 months | 73·0 (21·8) | 73·5 (21·2) | 64·6 (29·3) | -8·5 (-24·0, 7·0) |  |
|  | Alive > 12 months | 79·2 (24·4) | 77·9 (19·6) | 81·3 (19·8) | 2·1 (-2·9, 7·1) |  |
| **Fatigue** | Alive < 3 months | 43·8 (23·5) | 77·1 (28·1) | 81·3 (30·1) | 37·5 (-0·3, 75·3) | X |
|  | Alive 3-6 months | 35·1 (23·7) | 60·9 (27·6) | 64·9 (28·1) | 29·8 (18·5, 41·1) | X |
|  | Alive 6-12 months | 46·0 (25·8) | 44·4 (19·2) | 46·8 (25·1) | 0·8 (-12·7, 14·3) |  |
|  | Alive > 12 months | 38·3 (26·1) | 43·0 (26·7) | 41·4 (25·3) | 3·1 (-3·2, 9·4) |  |

Number of responders: Alive < 3 months: 8; Alive 3-6 months: 28; Alive 6-12 months: 11; Alive > 12 months: 69. Note: Higher scores for overall quality of life and functioning indicate better quality of life and function, higher scores for symptoms indicate higher symptom intensity. “X” indicates clinically meaningful change in scores (≥10 points) from inclusion to month 2.

**Supplementary Table 13. Change in patient-reported outcomes from inclusion to month 2 for complete responders at M0-M2, by survival groups according to survival after date of BM diagnosis, non-lung cancer group.**

| **EORTC Scale** | **Survival group** | **Inclusion** | **Month 1** | **Month 2** | **Mean change (CI)** | **≥ 10 points**  **change** |
| --- | --- | --- | --- | --- | --- | --- |
|  | | **Mean (SD)** | | |  |  |
| **Overall Quality of Life** | Alive < 3 months | 56·7 (30·3) | 20·0 (13·9) | 20·0 (13·9) | -36·7 (-70·7, -2·7) | X |
|  | Alive 3-6 months | 54·9 (21·9) | 53·9 (22·9) | 44·6 (28·0) | -10·3(-20·7, 0·1) | X |
|  | Alive 6-12 months | 56·5 (23·3) | 58·5 (24·0) | 57·8 (24·6) | 1·4 (-6·4, 9·1) |  |
|  | Alive > 12 months | 65·1 (23·2) | 63·4 (22·8) | 61·2 (23·0) | -3·9 (-8·5, 0·7) |  |
| **Physical function** | Alive < 3 months | 46·7 (34·6) | 22·2 (20·8) | 15·6 (21·7) | -31·1 (-82·2, 20·0) | X |
|  | Alive 3-6 months | 65·0 (29·6) | 61·3 (32·3) | 43·8 (33·8) | -21·2 (-33·9, -8·6) | X |
|  | Alive 6-12 months | 70·3 (25·2) | 76·2 (21·1) | 72·8 (23·8) | 2·5 (-4·5, 9·5) |  |
|  | Alive > 12 months | 83·3 (17·8) | 80·4 (18·5) | 80·1 (18·1) | -3·1 (-6·8, 0·5) |  |
| **Fatigue** | Alive < 3 months | 53·3 (21·7) | 86·7 (7·5) | 90·0 (9·1) | 36·7 (14·0, 59·3) | X |
|  | Alive 3-6 months | 38·4 (27·2) | 46·6 (29·2) | 57·6 (27·7) | 19·2 (8·9, 29·4) | X |
|  | Alive 6-12 months | 38·4 (25·7) | 42·0 (25·0) | 41·5 (25·3) | 3·1 (-5·3, 11·4) |  |
|  | Alive > 12 months | 32·8 (22·3) | 38·9 (24·1) | 39·6 (23·8) | 6·8 (1·9, 11·8) |  |

Number of responders: Alive < 3 months: 5; Alive 3-6 months: 34; Alive 6-12 months: 49; Alive > 12 months: 95. Note: Higher scores for overall quality of life and functioning indicate better quality of life and function, higher scores for symptoms indicate higher symptom intensity. “X” indicates clinically meaningful change in scores (≥10 points) from inclusion to month 2.

**Supplementary Table 14. Change in patient-reported outcomes from inclusion to month 2 for complete responders at M0-M2, by treatment group**

| **EORTC Scale** | **Treatment groups** | **Inclusion** | **Month 1** | **Month 2** | **Mean change (CI)**  **Inclusion vs· Month 2** | **≥ 10 points**  **change** | |
| --- | --- | --- | --- | --- | --- | --- | --- |
|  |  | **Mean (SD)** | | |  |  | |
| **Overall Quality of Life** | Surgery | 64·1 (24·8) | 65·2 (22·3) | 62·3 (23·9) | -1·7 (-8·3, 4·9) |  | |
|  | SRT | 59·0 (22·9) | 60·0 (22·3) | 56·7 (24·6) | -2·2 (-6·5, 2·0) |  | |
|  | WBRT | 57·7 (23·8) | 53·3 (24·3) | 48·0 (24·1) | -9·6 (-15·1, -4·2) |  | |
|  | Systemic | 64·6 (30·1) | 68·8 (24·3) | 64·6 (22·6) | 0·0 (-24·7, 24·7) |  | |
| **Physical function** | Surgery | 77·9 (23·2) | 77·6 (21·9) | 78·3 (20·3) | 0·4 (-5·0, 5·8) |  | |
|  | SRT | 75·2 (26·0) | 75·2 (22·2) | 70·4 (27·0) | -4·8 (-9·3, -0·2) |  | |
|  | WBRT | 74·0 (22·5) | 64·4 (28·8) | 57·1 (34·7) | -16·9 (-23·2, -10·6) | X | |
|  | Systemic | 83·3 (19·7) | 80·6 (22·0) | 86·1 (11·5) | 2·8 (-14·2, 19·8) |  | |
| **Emotional function** | Surgery | 80·3 (21·9) | 81·6 (23·0) | 83·5 (20·0) | 3·2 (-1·7, 8·1) |  | |
|  | SRT | 77·3 (23·8) | 81·7 (23·1) | 79·1 (25·1) | 1·8 (-1·7, 5·3) |  | |
|  | WBRT | 77·1 (22·0) | 77·3 (27·4) | 76·0 (27·6) | -1·1 (-6·3, 4·0) |  | |
|  | Systemic | 79·2 (29·2) | 83·3 (15·4) | 91·7 (8·9) | 12·5 (-15·1, 40·1) | X | |
| **Fatigue** | Surgery | 35·3 (25·2) | 38·1 (23·2) | 38·5 (24·2) | 3·2 (-2·9, 9·3) |  | |
|  | SRT | 38·5 (25·2) | 43·3 (27·0) | 44·2 (26·4) | 5·7 (0·7, 10·7) |  | |
|  | WBRT | 38·4 (24·5) | 53·8 (27·3) | 58·0 (27·7) | 19·6 (13·8, 25·4) | X | |
|  | Systemic | 22·9 (15·3) | 27·1 (17·7) | 22·9 (17·7) | 0·0 (-21·1, 21·1) |  | |
| **Nausea/vomiting** | Surgery | 10·3 (21·7) | 9·5 (19·4) | 12·0 (21·5) | 1·7 (-4·1, 7·5) |  | |
|  | SRT | 9·5 (19·5) | 10·1 (19·7) | 12·3 (21·6) | 2·8 (-0·9, 6·5) |  | |
|  | WBRT | 12·2 (21·5) | 23·0 (27·2) | 29·4 (30·7) | 17·2 (10·4, 23·9) | X | |
|  | Systemic | 8·3 (15·4) | 4·2 (11·8) | 8·3 (15·4) | 0·0 (-14·9, 14·9) |  | |
| **Pain** | Surgery | 21·4 (25·0) | 22·3 (26·7) | 22·4 (26·4) | 1·1 (-5·4, 7·5) |  | |
|  | SRT | 21·1 (26·7) | 23·2 (27·0) | 25·7 (28·7) | 4·6 (-0·2, 9·4) |  | |
|  | WBRT | 18·8 (24·3) | 20·5 (25·3) | 27·8 (31·3) | 9·0 (3·0, 15·1) |  | |
|  | Systemic | 20·8 (31·8) | 27·1 (28·1) | 25·0 (28·1) | 4·2 (-13·7, 22·0) |  | |
| **Dyspnea** | Surgery | 19·0 (27·3) | 21·1 (28·7) | 21·2 (26·4) | 2·2 (-3·6, 8·0) |  | |
|  | SRT | 22·4 (26·4) | 26·3 (27·1) | 26·3 (29·1) | 3·9 (-0·3, 8·2) |  | |
|  | WBRT | 20·1 (27·0) | 31·4 (30·3) | 32·7 (31·0) | 12·6 (6·6, 18·7) | X | |
|  | Systemic | 25·0 (29·5) | 25·0 (29·5) | 16·7 (25·2) | -8·3 (-28·0, 11·4) |  | |
| **Sleep disturbance** | Surgery | 39·7 (34·4) | 26·8 (29·1) | 24·4 (28·8) | -15·4 (-23·7, -7·0) | X | |
|  | SRT | 30·0 (29·1) | 26·1 (27·5) | 22·5 (24·5) | -7·5 (-12·6, -2·4) |  | |
|  | WBRT | 33·7 (29·5) | 34·6 (33·1) | 24·5 (30·4) | -9·2 (-15·9, -2·4) |  | |
|  | Systemic | 25·0 (15·4) | 20·8 (24·8) | 12·5 (17·3) | -12·5 (-26·9, 1·9) | X | |
| **Appetite loss** | Surgery | 11·3 (23·3) | 14·7 (23·9) | 18·6 (26·8) | 7·4 (0·9, 13·8) |  | |
|  | SRT | 13·7 (25·1) | 17·2 (25·9) | 21·3 (28·4) | 7·6 (2·2, 12·9) |  | |
|  | WBRT | 17·2 (28·7) | 28·6 (32·5) | 45·0 (36·7) | 27·8 (19·3, 36·3) | X | |
|  | Systemic | 12·5 (24·8) | 4·2 (11·8) | 16·7 (25·2) | 4·2 (-27·2, 35·5) |  | |
| **Constipation** | Surgery | 17·1 (27·8) | 17·3 (25·7) | 17·1 (24·5) | 0·0 (-7·7, 7·7) |  | |
|  | SRT | 23·0 (28·7) | 20·2 (30·4) | 19·6 (28·3) | -3·4 (-8·8, 2·1) |  | |
|  | WBRT | 24·8 (30·6) | 29·8 (31·7) | 33·7 (34·6) | 8·8 (-1·6, -16·0) |  | |
|  | Systemic | 4·2 (11·8) | 20·8 (24·8) | 8·3 (15·4) | 4·2 (-13·7, 22·0) |  | |
| **Headaches** | Surgery | 22·8 (29·0) | 20·8 (26·0) | 19·0 (27·6) | -3·8 (-11·5, 3·9) |  | |
|  | SRT | 13·8 (22·2) | 19·4 (23·8) | 15·7 (22·4) | 1·9 (-2·2, 6·0) |  | |
|  | WBRT | 17·6 (24·6) | 16·3 (22·3) | 19·2 (24·4) | 1·6 (-3·8, 7·1) |  | |
|  | Systemic | 8·3 (15·4) | 16·7 (17·8) | 20·8 (24·8) | 12·5 (-1·9, 26·9) | X | |
| **Visual disorder** | Surgery | 11·0 (19·3) | 11·4 (19·9) | 11·8 (20·1) | 0·8 (-3·6, 5·1) |  | |
|  | SRT | 13·3 (21·1) | 12·2 (19·1) | 12·4 (18·7) | -0·9 (-4·3, 2·5) |  | |
|  | WBRT | 12·7 (18·6) | 14·2 (20·6) | 16·4 (21·2) | 3·7 (0·3, 7·1) |  | |
|  | Systemic | 8·3 (23·6) | 4·1 (11·8) | 4·2 (11·8) | -4·2 (-14·0, 5·7) |  | |
| **Seizures** | Surgery | 2·1 (8·2) | 1·7 (9·1) | 1·3 (6·4) | -0·8 (-2·9, 1·2) |  | |
|  | SRT | 4·0 (14·6) | 9·1 (20·2) | 5·4 (15·1) | 1·4 (-2·0, 4·9) |  | |
|  | WBRT | 5·8 (16·5) | 8·7 (21·9) | 6·1 (17·9) | 0·3 (-3·6, 4·2) |  | |
|  | Systemic | 4·2 (11·8) | 4·2 (11·8) | 4·2 (11·8) | 0·0 (-14·9, 14·9) |  | |
| **Motor dysfunction** | Surgery | 20·3 (22·2) | 19·5 (21·1) | 19·0 (18·9) | -1·3 (-5·6, 3·0) |  | |
|  | SRT | 22·8 (26·1) | 20·7 (20·7) | 22·1 (24·7) | -0·6 (-4·7, 3·5) |  | |
|  | WBRT | 24·3 (22·7) | 30·8 (26·0) | 34·0 (29·9) | 9·7 (3·4, 15·6) |  | |
|  | Systemic | 6·9 (11·8) | 2·8 (5·1) | 6·9 (10·2) | 0·0 (-8·6, 8·6) |  | |
| **Communication deficit** | Surgery | 9·6 (13·4) | 8·0 (13·5) | 8·3 (13·1) | -1·3 (-4·1, 1·6) |  | |
|  | SRT | 14·7 (23·1) | 10·4 (18·4) | 11·5 (20·8) | -3·3 (-6·8, 0·2) |  | |
|  | WBRT | 14·7 (21·4) | 16·1 (22·4) | 18·9 (23·6) | 4·1 (-0·9, 9·1) |  | |
|  | Systemic | 0·0 (0·0) | 1·4 (3·9) | 2·8 (5·1) | 2·8 (-1·5, 7·1) |  | |
| **Drowsiness** | Surgery | 25·7 (23·8) | 34·6 (23·7) | 29·1 (20·2) | 3·4 (-2·3, 9·1) |  | |
|  | SRT | 32·5 (27·9) | 36·4 (26·5) | 36·7 (25·5) | 4·2 (-0·8, 9·2) |  | |
|  | WBRT | 33·0 (25·7) | 48·1 (28·2) | 53·8 (29·9) | 20·8 (14·1, 27·6) | X | |
|  | Systemic | 28·6 (23·0) | 16·7 (17·8) | 14·3 (17·8) | -14·3 (-30·8, 2·2) | X | |
| **Weakness of legs** | Surgery | 17·1 (26·2) | 25·6 (28·9) | 21·8 (27·3) | 4·7 (-3·1, 12·5) |  | |
|  | SRT | 21·7 (28·5) | 30·6 (30·3) | 29·6 (30·6) | 8·0 (2·0, 14·0) |  | |
|  | WBRT | 23·1 (27·1) | 43·0 (34·8) | 45·5 (36·0) | 22·4 (15·3, 29·6) | X | |
|  | Systemic | 16·7 (25·2) | 19·0 (17·8) | 12·5 (17·3) | -4·2 (-31·8, 23·5) |  |  |

Number of responders: Surgery: 79; SRT: 122; WBRT: 105; Systemic: 8. Note: Higher scores for overall quality of life and functioning indicate better quality of life and function, higher scores for symptoms indicate higher symptom intensity. “X” indicates clinically meaningful change in scores (≥10 points) from inclusion to month 2. SRT: Stereotactic radiotherapy; WBRT: Whole brain radiotherapy. QoL: Quality of life.

**Supplementary Table 15. Change in patient-reported outcomes from inclusion to month 2 for complete responders at M0-M2, by ECOG-status at inclusion, lung cancer group.**

| **EORTC Scale** | **ECOG** | **Inclusion** | **Month 1** | **Month 2** | **Mean change (CI)** | **≥ 10 points**  **change** |
| --- | --- | --- | --- | --- | --- | --- |
|  | | **Mean (SD)** | | |  |  |
| **Overall Quality of Life** | ECOG 0 | 73·7 (17·7) | 67·9 (19·7) | 60·9 (21·1) | -12·8 (-21·6, -4·0) | X |
|  | ECOG 1 | 61·7 (23·0) | 61·9 (22·8) | 57·8 (23·1) | -3·9 (-11·0, 3·3) |  |
|  | ECOG 2 | 41·3 (26·8) | 50·6 (21·3) | 46·0 (26·0) | 4·7 (-8·9, 18·2) |  |
|  | ECOG 3-4 | 48·5 (17·4) | 40·9 (26·2) | 37·9 (21·2) | -10·6 (-28·8, 7·6) | X |
| **Physical function** | ECOG 0 | 93·6 (9·0) | 84·5 (17·7) | 76·5 (26·2) | -17·1 (-27·8, -6·4) | X |
|  | ECOG 1 | 80·6 (18·9) | 72·6 (22·5) | 73·0 (28·1) | -7·6 (-15·2, 0·0) |  |
|  | ECOG 2 | 65·3 (21·1) | 56·2 (27·2) | 49·3 (34·4) | -16·0 (-30·2, -1·8) | X |
|  | ECOG 3-4 | 35·4 (19·1) | 40·4 (26·9) | 43·4 (31·2) | 8·1 (-14·8, 31·0) |  |
| **Fatigue** | ECOG 0 | 23·7 (19·5) | 41·7 (27·4) | 48·1 (35·1) | 24·4 (12·7, 36·0) | X |
|  | ECOG 1 | 38·5 (21·2) | 46·4 (27·6) | 44·3 (25·1) | 5·7 (-1·8, 13·3) |  |
|  | ECOG 2 | 56·4 (28·7) | 58·6 (22·3) | 64·7 (25·5) | 8·3 (-6·2, 22·9) |  |
|  | ECOG 3-4 | 36·4 (29·6) | 63·6 (33·2) | 51·5 (32·9) | 15·2 (-8·6, 38·9) | X |

Number of responders: ECOG 0: 26; ECOG 1: 61; ECOG 2: 26; ECOG 3-4: 11. Note: Higher scores for overall quality of life and functioning indicate better quality of life and function, higher scores for symptoms indicate higher symptom intensity. “X” indicates clinically meaningful change in scores (≥10 points) from inclusion to month 2. ECOG: Eastern Cooperative Oncology Group.

**Supplementary Table 16. Change in patient-reported outcomes from inclusion to month 2 for complete responders at M0-M2, by ECOG-status at inclusion, non-lung cancer group.**

| **EORTC Scale** | **ECOG** | **Inclusion** | **Month 1** | **Month 2** | **Mean change (CI)** | **≥ 10 points**  **change** |
| --- | --- | --- | --- | --- | --- | --- |
|  | | **Mean (SD)** | | |  |  |
| **Overall Quality of Life** | ECOG 0 | 69·9 (20·8) | 69·3 (20·8) | 61·7 (23·4) | -8·2 (-14·8, -1·5) |  |
|  | ECOG 1 | 61·2 (24·4) | 56·5 (24·7) | 58·2 (23·6) | -3·0 (-8·3, 2·4) |  |
|  | ECOG 2 | 50·0 (17·2) | 50·6 (24·2) | 45·7 (30·4) | -4·3 (-14·5, 5·9) |  |
|  | ECOG 3-4 | 41·7 (23·0) | 50·0 (21·3) | 40·3 (27·0) | -1·4 (-26·3, 23·5) |  |
| **Physical function** | ECOG 0 | 89·5 (13·2) | 86·7 (16·2) | 83·4 (17·1) | -6·0 (-10·3, -1·8) |  |
|  | ECOG 1 | 80·1 (15·7) | 75·9 (20·4) | 72·9 (23·4) | -7·2 (-12·6, -1·9) |  |
|  | ECOG 2 | 60·2 (25·6) | 59·0 (29·1) | 47·3 (35·4) | -12·9 (-26·1, 0·3) | X |
|  | ECOG 3-4 | 22·2 (17·1) | 43·1 (31·3) | 41·7 (31·8) | 19·4 (-2·9, 41·8) | X |
| **Fatigue** | ECOG 0 | 24·7 (19·3) | 32·5 (23·0) | 34·8 (21·2) | 10·1 (3·1, 17·0) | X |
|  | ECOG 1 | 37·6 (23·5) | 43·9 (25·2) | 46·4 (27·8) | 8·9 (2·9, 14·8) |  |
|  | ECOG 2 | 47·3 (22·4) | 52·7 (28·3) | 58·6 (27·2) | 11·3 (2·2, 20·4) | X |
|  | ECOG 3-4 | 53·0 (32·3) | 55·6 (27·8) | 48·5 (24·1) | -4·5 (-28·6, 19·5) |  |

Number of responders: ECOG 0: 58; ECOG 1: 79; ECOG 2: 31; ECOG 3-4: 12. Note: Higher scores for overall quality of life and functioning indicate better quality of life and function, higher scores for symptoms indicate higher symptom intensity. “X” indicates clinically meaningful change in scores (≥10 points) from inclusion to month 2. ECOG: Eastern Cooperative Oncology Group.

**Supplementary Table 17 Change in patient-reported outcomes from inclusion to month 2 for complete responders at M0-M2, by DS-GPA group.**

| **EORTC Scale** | **DS-GPA group** | **Inclusion** | **Month 1** | **Month 2** | **Mean change (CI)** | **≥ 10 points**  **change** |
| --- | --- | --- | --- | --- | --- | --- |
|  | | **Mean (SD)** | | |  |  |
| **Overall Quality of Life** | 0-1·0 | 55·7 (24·2) | 53·5 (22·7) | 47·4 (24·6) | -8·3 (-14·9, -1·7) |  |
|  | 1·5-2·0 | 61·7 (22·3) | 59·0 (22·9) | 60·0 (23·5) | -1·7 (-6·6, 3·1) |  |
|  | 2·5-3·0 | 61·9 (24·0) | 66·7 (21·4) | 56·8 (25·0) | -5·1 (-11·0, 0·9) |  |
|  | 3·5-4·0 | 61·9 (18·5) | 69·4 (22·2) | 69·0 (17·8) | 7·1 (-12·5, 26·8) |  |
| **Physical function** | 0-1·0 | 66·4 (27·9) | 57·6 (28·8) | 51·6 (33·7) | -14·8 (-22·4, -7·2) | X |
|  | 1·5-2·0 | 80·7 (22·1) | 77·7 (21·9) | 75·0 (25·6) | -5·7 (-10·7, -0·7) |  |
|  | 2·5-3·0 | 82·7 (13·7) | 80·9 (17·9) | 76·5 (21·5) | -6·2 (-11·8, -0·7) |  |
|  | 3·5-4·0 | 95·2 (5·9) | 90·7 (13·0) | 92·1 (5·4) | -3·2 (-10·9, 4·6) |  |
| **Fatigue** | 0-1·0 | 41·7 (25·8) | 55·6 (26·5) | 57·0 (30·4) | 15·4 (8·2, 22·6) | X |
|  | 1·5-2·0 | 36·8 (26·1) | 43·4 (26·8) | 42·9 (25·7) | 6·1 (1·2, 11·1) |  |
|  | 2·5-3·0 | 33·1 (20·9) | 35·2 (22·6) | 41·7 (23·7) | 8·6 (2·1, 15·1) |  |
|  | 3·5-4·0 | 28·6 (8·1) | 36·1 (26·7) | 31·0 (15·0) | 2·4 (-8·3, 13·0) |  |

Number of responders: 0-1·0: 90; 1·5-2·0: 105; 2·5-3·0: 59; 3·5-4·0: 7. Note: Higher scores for overall quality of life and functioning indicate better quality of life and function, higher scores for symptoms indicate higher symptom intensity. “X” indicates clinically meaningful change in scores (≥10 points) from inclusion to month 2.

**Supplementary Table 18. Change in patient-reported outcomes from inclusion to month 2 for complete responders at M0-M2, by RPA group.**

| **EORTC Scale** | **RPA class** | **Inclusion** | **Month 1** | **Month 2** | **Mean change (CI)** | **≥ 10 points**  **change** |
| --- | --- | --- | --- | --- | --- | --- |
|  | | **Mean (SD)** | | |  |  |
| **Overall Quality of Life** | 1 | 48·8 (24·0) | 59·7 (27·9) | 51·2 (28·8) | 2·4 (-12·6, 17·4) |  |
|  | 2 | 61·7 (23·1) | 60·7 (22·3) | 56·8 (24·4) | -4·9 (-8·3, -1·4) |  |
|  | 3 | 46·7 (20·7) | 42·5 (23·2) | 41·7 (23·3) | -5·0 (-20·8, 10·8) |  |
| **Physical function** | 1 | 69·8 (29·0) | 74·1 (14·5) | 65·9 (26·3) | -4·0 (-27·4, 19·4) |  |
|  | 2 | 81·4 (18·4) | 74·5 (24·1) | 70·1 (29·3) | -11·2 (-14·7, -7·8) | X |
|  | 3 | 28·3 (19·6) | 39·7 (29·4) | 43·3 (30·6) | 15·0 (-1·9, 31·9) | X |
| **Fatigue** | 1 | 46·4 (6·8) | 41·7 (24·1) | 57·1 (29·8) | 10·7 (-6·0, 27·4) | X |
|  | 2 | 36·3 (28·0) | 44·4 (26·6) | 46·3 (27·5) | 10·0 (6·4, 13·7) | X |
|  | 3 | 42·1 (31·1) | 59·2 (30·3) | 47·4 (29·0) | 5·3 (-13·3, 23·8) |  |

Number of responders: 1: 14; 2: 228; 3: 20. Note: Higher scores for overall quality of life and functioning indicate better quality of life and function, higher scores for symptoms indicate higher symptom intensity. “X” indicates clinically meaningful change in scores (≥10 points) from inclusion to month 2.

**Supplementary Table 19. Change in patient-reported outcomes from inclusion to month 2 for complete responders at M0-M2, by Simplified score group.**

| **EORTC Scale** | **Simplified score group** | **Inclusion** | **Month 1** | **Month 2** | **Mean change (CI)** | **≥ 10 points**  **change** |
| --- | --- | --- | --- | --- | --- | --- |
|  | | **Mean (SD)** | | |  |  |
| **Overall Quality of Life** | 1 | 62·8 (21·8) | 62·6 (23·0) | 58·1 (22·8) | -4·7 (-9·2, -0·1) |  |
|  | 2a | 67·2 (21·6) | 63·3 (21·2) | 60·0 (23·8) | -7·3 (-13·0, -1·6) |  |
|  | 2b | 43·6 (20·9) | 48·0 (23·1) | 46·6 (28·4) | 2·9 (-8·6, 14·5) |  |
|  | 3 | 48·0 (23·8) | 49·5 (22·3) | 43·6 (25·0) | -4·4 (-14·9, 6·0) |  |
| **Physical function** | 1 | 84·0 (16·0) | 79·1 (20·3) | 76·2 (23·9) | -7·7 (-12·1, -3·4) |  |
|  | 2a | 86·0 (15·1) | 78·2 (20·7) | 73·9 (26·3) | -12·0 (-17·8, -6·2) | X |
|  | 2b | 54·6 (28·5) | 54·9 (30·5) | 53·9 (32·4) | -0·7 (-14·3, 13·0) |  |
|  | 3 | 52·3 (26·9) | 50·9 (28·9) | 39·9 (33·1) | -12·4 (-25·6, 0·8) | X |
| **Fatigue** | 1 | 34·1 (22·5) | 42·2 (24·9) | 42·9 (26·2) | 8·8 (4·2, 13·4) |  |
|  | 2a | 32·2 (22·1) | 41·4 (27·9) | 44·3 (28·5) | 12·1 (5·6, 18·6) | X |
|  | 2b | 51·5 (29·9) | 56·6 (27·3) | 55·1 (30·8) | 3·5 (-9·3, 16·4) |  |
|  | 3 | 46·2 (26·2) | 54·5 (26·2) | 58·6 (23·7) | 12·4 (2·1, 22·6) | X |

Number of responders: 01: 108; 2a: 88; 2b: 34; 3: 35. Note: Higher scores for overall quality of life and functioning indicate better quality of life and function, higher scores for symptoms indicate higher symptom intensity. “X” indicates clinically significant change in scores (≥10 points) from inclusion to month 2.
