# Supplementary material for: Survival and quality of life after first-time diagnosis of brain metastases: a multicenter, prospective, observational study
Source: Lancet Reg Health Eur. 2024 Dec 19;49:101181. doi: 10.1016/j.lanepe.2024.101181 (PMC11728971; doi:10.1016/j.lanepe.2024.101181)
Supplement: Study Protocol [file mmc2.pdf]

Brain metastases in Norway - Improved classification and treatment

-a prospective cohort study

Principal investigator: PhD/MD Olav Erich Yri[1]

Co-principal investigator: Prof./PhD, MD Stein Kaasa[1,2,3]

Study management group: Prof./PhD MD Jon Håvard Loge[4,5], Prof./PhD Marianne J. Hjermstad[3,4], Prof./PhD MD Nina Aass[1,2,4,]

[1]Dept. of Oncology, Oslo University Hospital (OUS), [2]Faculty of Medicine, University of Oslo, [3]European Palliative Care Research Centre, NTNU, Norway, [4]Regional Advisory Unit for Palliative Care, Dept. of Oncology, Oslo University Hospital (OUS), [5]Dept. of Behavioral Sciences in Medicine, University of Oslo (UiO)

Content

1. Introduction 3

2. Background 3

3. Materials and methods 7

3.1. Study population 7

3.1.1. Incidence estimations and identification of eligible participants 8

3.2.1. Inclusion criteria 8

3.2.2. Exclusion criteria 9

3.3. Study centers 9

3.4. Data recording 9

3.4.1. Linking of data from other registries or sources 10

3.4.2. Inclusion of patients and follow-up 10

3.4.3. Registration at time of inclusion 10

3.4.4. Registration at follow-up 14

3.4.5. Registration at time of death 16

3.4.6. Long-term follow-up after 24 months or more after inclusion 17

3.4.7. Flow chart for registrations 19

3.5. Biobanking and analysis of tissue, including genetic analyses 20

3.6. Procedure for informing the patient if genetic analyses reveal conditions that may influence the patient's health 20

3.7. Procedure for issuing patient questionnaires 21

4. Ethical considerations 21

4.1. Ethics approval 21

4.2. Informed consent 21

4.3. Treatment of patients 22

4.4. Risk 22

4.5. Data collection, data storage, data exchange, data linking, and patient confidentiality 23

4.6. Liability and insurance 24

5. Study management 24

6. Quality assurance 26

7. Definition of end of inclusion and end of study 26

8. Finances and conflict of interest 26

9. Publication and dissemination 27

10. References 28

1. Introduction

Brain metastases (BMs) are the most common brain tumors and represent a major cause of morbidity and mortality in cancer patients. The incidence of BM is increasing but despite several and also new treatment options and a substantial use of health care resources, the scientific basis on how to manage patients with BMs remains meager. Core questions are when and in what order the different modalities should be administered, but also when to stop tumor directed treatment. Presently, classification according to prognosis, which should guide treatment decisions, is being partly based upon clinical “gut-feeling” as opposed to a solid evidence base. Patients are therefore at risk of both “over- and under-treatment”, resulting in suboptimal use of health care resources and unnecessary burdening of the patients and their caregivers.

Treatment options for BM are steroids, surgery, stereotactic radiosurgery, or whole brain radiotherapy, used either separate or in combinations. However, no national guideline on treatment of BM is established in Norway and the scientific basis of current international guidelines is weak and derived from retrospective studies and a limited number of randomized clinical trials. All guidelines point to limitations in the data they are based upon. Because of this, the treatment practice in Norway is most likely variable but remains uncertain as there is no national reporting on the management of BM at present. To what extent surgery and radiotherapy is offered to patients with BM in Norway is not known as is the outcome for those being offered the different treatment options. Therefore, we will conduct a prospective, population-based study by consecutively including all target patients in the South-Eastern and Middle health regions. The overall aim of the study is to gain insight into the current management of patients with BM. Data on patient and cancer characteristics, treatment, survival, and patient reported outcomes will be registered prospectively, establishing a complete BM study registry that will provide information to guide decision-making for clinicians and health care providers, for the development of an improved prognostic classification system, and for hypothesis generation for future trials.

2. Background

In Norway, approximately 33 000 persons are diagnosed with cancer each year. Although 5-year survival rates and mortality from cancer is improving, almost 11 000 persons died from cancer in Norway in 2014 (1). An estimated 10-40% of patients diagnosed with cancer eventually develop BM (2). Due to an increased cancer incidence in general, improved use of

high-resolution diagnostics such as MRI and PET, and increased survival from improvements in systemic therapy, the incidence of brain metastases (BM) is presumed to be rising (3). As such, clinically BM is now observed even in patients with cancers that previously were not associated with BM such as prostate cancer. This indicates that health care resources needed to treat BM will increase, and it is imperative that these resources be used rationally to ensure optimal patient care.

BM imposes a significant disease burden on patients and their relatives. Many patients have cognitive impairments such as memory loss and reduced executive functions (4). Optimal care and management of BM patients is therefore warranted. Treatment options include steroids, surgery, stereotactic surgery (SRS), whole brain radiation therapy (WBRT), and systemic treatments such as chemotherapy. Although several BM treatment guidelines are published (5, 6), the coherence to them is variable (7), and the scientific basis is mostly data from retrospective studies and a relatively limited number of RCTs that tend to be older, underpowered, and use different end-points. In addition, RCTs and retrospective reports most often include only patients referred to larger hospitals with radiation and/or neurosurgical resources. This may lead to a significant selection bias, leaving out the most vulnerable and frail patients that may have the most to gain from an evidence-based approach to end-of-life care. As a result, BM treatment decision making is often driven by institutional tradition and routine and the preferences of the individual physician. There are no national guidelines or consensus on the best BM treatment approach in Norway and hence there is a substantial risk of both over- and under-treatment that may cause unwanted complications, morbidity, and mortality for the patients and an inappropriate use of health care resources.

Historically, median survival in BM is reported to one month untreated, increasing to two months by treatment with steroids alone and three to six months by treatment with WBRT combined with steroids (8). These observations probably mirror a selection to type of treatments, despite lack of agreements on any formal classification systems for BM internationally. WBRT is traditionally regarded as the “standard” treatment for BM, especially for patients with multiple metastases and a poor prognosis. However, WBRT has recently been shown to be ineffective in terms of overall survival and quality of life (QoL) in NSCLC patients with poor performance status (9), and WBRT has been reported to be associated with brain atrophy, declined neurocognitive functioning, and reduced QoL after treatment (10-13).

Given the potential side effects of WBRT and in an effort to improve survival rates, surgery and SRS have been explored as treatment options in patients with limited disease (up to 4 metastases). In single BM, trials have shown that surgery followed by WBRT increase local control compared to WBRT alone, but with limited improvement in survival and with substantial heterogeneity between trials (14). Conversely, a later RCT comparing surgery alone to surgery followed by WBRT found that adding WBRT after surgery improved intracranial control but did not improve survival or time of functional independence (15).

In recent years, the use of SRS has been introduced as an alternative to surgery in limited BM, and several RCTs have been conducted on patients with up to 4 BMs. From available meta-analyses of these trials, it is evident that combining SRS with WBRT improves intracranial control but does not improve overall survival compared to either SRS or WBRT alone (16, 17). Lately, SRS is suggested feasible for treatment for multiple lesions (>4) as well (18), and is the subject of an on-going RCT (NCT01592968). Then, what remains unanswered is whether WBRT should be added after initial treatment with SRS or saved as an option to patients that have intracranial recurrence or progression.

This unanswered issue, combined with the fact that the needs of information and supportive care for patients diagnosed with BM and their caregivers have been poorly addressed (19), has encouraged recent trials to use neurocognitive function and QoL as primary outcomes rather than survival times and intracranial control rates. The results from these trials are divergent and difficult to compare as they often use different designs and end-points. Also, there are considerable confounding factors and challenges with compliance to protocol procedures in QoL-studies involving a patient group with a considerable disease burden. However, in general, WBRT is associated with a more severe reduction in neurocognitive functioning and quality of life than treatment with SRS (11-13), and SRS alone with surveillance and salvage therapy if intracranial progression occurs is now recommended for patients with limited number of BM in the 2014 ASTRO “Choosing Wisely” statements (20). With this strategy, a considerable proportion of patients may achieve intracranial control without exposure to WBRT and its possible detrimental effects (12). However, due to the divergent results of the recent trials on the effects of WBRT, its role in treatment of BM is heavily debated (21, 22).

Systemic treatments have had an insignificant effect on BM to date, but novel agents such as tyrosine kinase inhibitors (TKI) and immunotherapy show promising effects (23).

Most likely, the same treatment strategy should not be applied to all patients with BM. When discussing the treatment of choice, the attending physician should take into account factors related both to the patient and the intracranial and extracranial disease status. However, which factors that should be considered are not clear, especially in the rapidly evolving era of personalized medicine with systemic treatment based on tumor-specific markers. Several classification systems have been proposed to assess the prognosis in patients with BM and some even validated in various patient cohorts (24). To what extent these systems are used by clinicians is most likely variable and they may not be applicable with current and future treatment options.

To the best of our knowledge, there is no current national or regional prospective registration of patients diagnosed with BM in Norway. To what extent and when surgery, SRS, and WBRT are offered is not known. The characteristics and outcomes of this heterogeneous and increasing patient group are largely unknown, and the available publications nationally and internationally are mostly on selected patient groups and not population-based. On this background, we will conduct a comprehensive, population-based, prospective clinical study collecting data on patient characteristics, tumor specific markers, treatment data, and patient reported outcome measures (PROMS) that will constitute a study registry. This prospective, clinical study registry will provide a source for improved understanding of the patients and the disease. One goal is to establish an international classification system for these patients and to establish a better basis for future clinical intervention trials. The collection of data will be made in a pragmatic and feasible manner ensuring a consecutive inclusion of patients with BM from solid cancers in all somatic hospitals in two health regions.

The overall aim and primary outcome of this study will be a descriptive analysis of the current treatment practice of BM in Norway. Specifically, it may give answers to the following research questions:

- What is the true incidence of BM in Norway?
- How are patients with BM treated at present?
- Do treatments differ between hospitals?
- How do treatments impact quality of life of the patients?
- Which factors (treatment, tumor and host variables) can explain disease control, survival, symptom relief, and general functions?
- How can BM staging be improved?

Secondary outcomes are:

- Assessment of patient symptoms, functions, and QoL
- Assessment of survival times after initial treatment of BM
- Health care related costs analysis of the treatments offered to the patients

3. Materials and methods

3.1. Study population

Any patient diagnosed with BM from solid cancers in the Middle- and South-East health regions of Norway from the date of start of the study is eligible for inclusion. The patients will be identified by results from radiology, by the attending physician or by other health care personnel involved in the patient's disease trajectory. They will then be invited to participate at the participating study center by the attending physician or by study personnel when a diagnosis of BM is done or at referral study centers if patients are referred to radiotherapy or surgery for their BM. Reports from the Norwegian Patient Register (NPR) will be used to identify patients not previously reported or included. In order to increase accuracy of incidence estimation patients identified in this way will be registered anonymously by recording the primary diagnosis and date of BM only. Potential participants will receive oral and written information and written consent must be provided prior to inclusion.

A proportion of eligible patients will be physically affected of disease so that a full procedure of oral and written information and consent may be challenging. Informed consent must nevertheless be provided in these situations. The informed consent may be confirmed by the patient's relatives, *i.e.* if the patient has difficulty in signing the consent form the ability.

Another group of patients may have cognitive impairment related to disease. If the patient is considered unable to receive adequate information and provide informed consent (according to "Pasient- og brukerrettighetsloven" §4-3), complete oral and written information may be given to the patient's relatives who then may give written consent on behalf of the patient.

Separate versions of written information have been developed for these situations.

3.1.1. Incidence estimations and identification of eligible participants

As described in 3.1, participants may be identified by results from radiology. Radiology reports from CT- and/or MRI of the brain will be the best source to identify patients diagnosed with brain metastases. To provide estimates of the incidence of brain metastases and the proportion and representativeness of participants that eventually are recruited to the study, for a restricted period of time, all radiology reports from CT/MRI – scans of the brain in selected hospitals will be screened for descriptions of brain metastases. One co-worker at the radiology departments at each hospital will print all reports from CT/MRI-scans weekly. These printed reports will be screened by project co-workers for descriptions of brain metastases. Reports from scans of patients that do not have brain metastases will be deleted immediately, as will reports that describe patients previously diagnosed with brain metastases. Reports that describe brain metastases diagnosed for the first time will be stored in accordance with regulations at the Oslo University Hospital. For these patients, the following data will be registered: Social security number, name, gender, date of CT/MRI, primary cancer diagnosis. These data will be stored separately in in accordance with regulations at the Oslo University Hospital using a separate code list. Patients that are included in the study will be removed from the data code list. For patients that are not recruited to the study, the following data will be stored: Age at time of brain metastases, gender, primary cancer. The code list will then be deleted two months after the registration period is ended.

3.2.1. Inclusion criteria

- Verified cancer diagnosis of solid tumors (based on radiological, histological/cytological or operative evidence).
- Brain metastases verified by computer tomography (CT), contrast-enhanced magnetic resonance imaging (MRI), or surgical biopsies
- Age ≥ 18 years
- Able to comply with study procedures
- Able to provide written informed consent after information in Norwegian given according to procedures described in the protocol

3.2.2. Exclusion criteria

- Primary brain tumors
- Primary hematological malignancies (lymphomas, leukemias)
- Previous diagnosis and/or treatment of BM
- Unable to produce written informed consent after information in Norwegian given according to procedures described in the protocol

3.3. Study centers

All somatic hospitals in the Middle- and South-East health regions that treat cancer patients will be invited to participate in the execution of the study. The study will start registration at the Oslo and St. Olav University Hospitals and will include the other centers consecutively. Each center must appoint a local primary investigator (PI) and should have access to study nurse or other support staff.

3.4. Data recording

Study data will be registered electronically through a web-based platform to be approved by the sponsor institution (Oslo University Hospital). This platform will ensure secure log-in by investigators and encryption of data before transfer, providing full confidentiality. Only the Chief Principal Investigator and the study nurse at the study center at Oslo University Hospital will have access to the data file generated by the electronic registration. Participating centers may choose to send data by paper-based clinical report forms (CRF), which will then be registered electronically by the CPI or study nurse at Oslo University Hospital. A study ID-number will be assigned to each patient at inclusion. The PI at each participating center is responsible for keeping a code list linking personal data of the included patients with the study ID-number. This code list must contain full name, social security number, address and phone number of the included patients. Only the study ID-number will be entered on the paper CRFs or in the web-based registration. Secure storage of the code list is the responsibility of the PI at each participating center. See also *4.5 Data collection, data storage, data exchange, and patient confidentiality.*

3.4.1. Linking of data from other registries or sources

After end of study, linking patient data with individual data from the following registries may be performed: Norwegian Patient Registry (NPR) with data on diagnostic information, medical treatment, hospital visits and admissions, discharge, etc., the Norwegian Cancer Registry with data from the primary cancer diagnosis, pathology reports, initial treatment reports, etc., and The Norwegian Cause of Death Registry with data for date of death, cause of death, and place of death. Information about linking patient and registry data is included in the patient information given to all patients prior to inclusion and in the application for ethics approval. See also *4.5 Data collection, data storage, data exchange, and patient confidentiality*.

3.4.2. Inclusion of patients and follow-up

To obtain an accurate estimation of the incidence, a complete inclusion of patients diagnosed with BM must be facilitated. Therefore, inclusion and initial registration will be made as “low-threshold” and practical as possible, and the initial data registration required by the local study center (see below) will be at a minimum. Completing data from time of inclusion and from time of follow-up will then be recorded by study personnel at the local study center or by the study center at Oslo University Hospital by correspondence with the local study center. At regular time intervals, the study management will perform reviews of data and the inclusion rate and inform participating centers on inclusion progression.

3.4.3. Registration at time of inclusion

To simplify inclusion and increase the likelihood for complete inclusion, the data required to be provided by health care professionals (HCP) at the local study site and by the patient are limited and must be considered a minimum requirement for including the patient in the study. Then, collecting and entering completing data from the time of inclusion will be the responsibility of study personnel at the local study center or the study center at Oslo University Hospital by correspondence with the local study center. These data will will contain further information on the patient’s medical history, oncology treatments, primary cancer, and BM.

a. Data to be provided by local study center health care professionals (HCP)

The HCP (local PI, doctor, or study nurse) on each participating site must give oral and written information to the patients about the study and obtain written informed consent in addition to registration of basic information about the patient, the primary cancer, and the diagnosis of BM. Patient name, gender, address, telephone number, and social security number must be entered into the study ID-code list (see *3.4 Data recording).* The code list and the signed informed consent forms must be properly stored.

Specifically, the HCP must provide:

1. Written informed consent
2. Patient name, gender, address, telephone number, and social security number

This information must be properly stored at the local study center.

The CRF at inclusion (paper or electronically) must contain:

1. Patient weight and height
2. Estimation of Eastern Cooperative Oncology Group (ECOG) – performance status at time of inclusion
3. Primary cancer type (lung, breast, etc.)
4. Date of BM diagnosis
5. BM treatment (surgery, radiotherapy (SRS or WBRT), systemic anti-cancer treatment, steroids)
6. Referred to other hospital (Yes/no)
7. Steroid dosage

This information will be registered on paper CRFs and sent to the study center at Oslo University Hospital by mail or electronically via the web-based tool (see above).

b. Data to be provided by patients

Patients will be asked to fill out a brief questionnaire on sociodemographic variables and life style related issues (marital status, living condition, employment status, smoking status, alcohol consumption, and education level). They will also be asked to fill out a set of widely used and validated quality of life (QoL) and symptoms questionnaires. The QLQ-C15-PAL, was

developed by the European Organization for Research and Treatment of Cancer (EORTC). It contains items on physical and emotional function and symptoms and is a widely used QoL questionnaire in cancer. The ***QLQ-BN20*** is a supplemental questionnaire to the QLQ-C30. It was originally developed for patients with primary brain tumors but is frequently used in BM studies and contains items related to function, disease symptoms, and future uncertainty. ***The EQ-5D*** is a widely used questionnaire to measure health state and calculation of cost-effectiveness and estimation of quality-adjusted life-years (QUALY).

The completed questionnaires must be sent by mail to the study center at Oslo University Hospital either by the patient or by the HCP at the local study center. Pre-addressed envelopes will be provided for this purpose.

c. Data to be provided by local study center or by Oslo University Hospital study center

These data will contain further information on the patient's medical history, oncology treatments, primary cancer, and BM. They will be collected by correspondence with the local study center and recorded electronically by the Oslo University Hospital study center.

Specifically, the following data will be collected:

1. Current medications
2. Oncologic history
    a. Date of primary cancer diagnosis
    b. TNM stage (or corresponding staging, *e.g.* FIGO or others) at diagnosis of primary cancer
    c. BM present at primary diagnosis (Yes/No)
    d. Sub-categorization of primary cancer in terms of histological subtype and result of analyses on mutations and/or protein expression as applicable
        i. Lung cancer [*e.g.* non-small cell/small cell, adenocarcinoma/ squamous/other, PD-1 (defined cut-off values), ALK/EGFR-analyses (wild-type/mutated/not analyzed)];
        ii. Breast cancer [*e.g.* ductal/lobular/tubular/other (specify), Estrogen/progesterone receptor (with quantification if available), HER-2 (positive/negative)];
        iii. Melanoma [*e.g.* BRAF (positive/negative/not analyzed)];

    iv. Gastrointestinal [*e.g.* mucinary, other, RAS/NRAS/MSI (positive/negative/not analyzed)]
    v. Kidney cancer [*e.g.* clear cell, other (specify)]
    vi. Gynecological cancer [*e.g.* cervical, ovarian, uterine, other (specify), other markers]
    vii. Other, specified

e. Previous and current cancer sub-type specific treatment(s) in pre-defined categories:
    i. Chemotherapy [a) Platinum derivatives, b) antimetabolites, c) alkaloids, d] taxanes, c] anthracyclines, d] cytotoxic antibiotics, e) other]
    ii. Protein kinase inhibitors [*e.g.* inhibitors of a) EGFR, b) ALK, c) HER-2/erb2, d) mTOR, e) VEGF/PDGFR, f) MEK, g) BRAF, h) other]
    iii. Immunotherapy [*e.g.* a) ipilimumab, b) PD1-inhibitors, c) PD-1 ligands, d) other]
    iv. Monoclonal antibodies [*e.g.* a) anti-HER-2, b) anti-VEGF, c) anti-EGFR, d) others]
    v. Others, specified

f. Extent of extracranial metastases at primary diagnosis [number of organs, which organs in predefined categories (lung, liver, bone, lymph nodes, other)]
g. Estimation of primary disease status at BM diagnosis (absent, response, stable, progression)
h. Extent of extracranial metastases at BM diagnosis [number of organs, which organs in predefined categories (lung, liver, bone, lymph nodes, other)]
i. Estimation of extracranial metastases status at BM diagnosis (response, stable, progression)

3. BM characteristics
    a. Department responsible for treatment at diagnosis of BM (pulmonary, palliative, oncology, gynecology, other, specified)
    b. Out-patient or in-patient at BM diagnosis
    c. Cause for BM discovery (symptoms, incidental/routine procedure)
    d. Symptoms and signs leading to BM diagnosis, in Yes/No predefined categories (cognitive decline, behavioral changes, seizures, nausea, vomiting, headaches, motory or sensory symptoms, other, specify)
    e. Mode of BM discovery (CT, MRI, both)

    f. Number of brain metastases
    g. Location of brain metastases (supra/infratentorial, left/right hemisphere, brain stem, leptomeningeal)
    h. Diameter of largest metastasis (mm)
    i. Classification system used to assess prognosis of survival after BM diagnosis (Yes/no, multiple choice)
    j. Place of care at BM diagnosis (home, hospice/care center)
    k. BM treatment data (surgery reports [where, when, result (complete resection, partial), complications], radiotherapy reports [SRS/WBRT, dosage, fractionation])
4. Dedicated palliative specialists involved in patient care (Yes/no)
5. Radiology reports of the latest evaluation of systemic disease and of BM diagnostics
6. Results of blood samples analyzed at the participating hospital's local clinical chemical laboratory at the time point closest to inclusion
    a. Hemoglobin, platelets, leucocytes, creatinine, albumin, CRP, ALAT, ASAT, GT, ALP, LD, bilirubin

3.4.4. Registration at follow-up

a. Patient questionnaires

Patient questionnaires will be sent by mail to patients every month for up to 12 months. The patients will be sent the following questionnaires: QLQ-C15-PAL, QLQ-BN20, and EQ-5D. The questionnaires will be sent to the patients by the study center at Oslo University Hospital and returned in a pre-addressed envelope for registration.

b. Clinical data

Follow-up time-points for clinical data will be at approximately every 3 months for up to ~~12~~ 24 months after inclusion or death, whichever comes first. Data from follow-up points will consist of a minimum of information from the patient's hospital record entered by health care professionals at the local site (if applicable at the corresponding time of follow-up), and data collected and recorded by the local study center or the Oslo University study center by correspondence with the local study center.

Local study center health care professionals (HCP)

When the patient is seen by their HCP, the following must be recorded in the patient's hospital records:

1. Patient weight
2. ECOG-status
3. Steroid use and dosage
4. Current place of care (home, hospital, palliative care unit, hospice/care center)

The local study center or the Oslo University study center

The following data will be collected from the patient's hospital records and registered by the local study center or main study center (if entered in the patient's hospital record close to (within 1-2 weeks) the corresponding time of follow-up):

1. Hospitalizations since last follow-up (number of admissions, date(s), cause(s) in pre-defined categories (infection, pain, intracranial symptoms (headache, nausea/vomiting, cranial nerve related symptoms, reduced cognitive function), general deterioration related to cancer, other)
2. Current medication
3. Current cancer treatment and planned changes in cancer treatment (if applicable)
4. Salvage treatment(s) of intracranial disease if applicable [steroids (including dosage), surgery, radiotherapy (SRS, WBRT), best supportive care (including steroids only)]
5. Status of systemic disease *as evident by clinical and/or radiological examinations as indicated* (response, stable, progression)
6. Status of intracranial disease *as evident by clinical and/or radiological examinations as indicated* (response, stable, progression, local progression, distal intracranial progression)
7. Dedicated palliative specialists involved in patient care (Yes/no)
8. Radiology reports of the latest evaluation of systemic disease and of BM
9. Results of blood samples analyzed at the participating hospital's local clinical chemical laboratory at the time point closest to the follow-up time point (within 1-2 weeks)

a) Hemoglobin, platelets, leucocytes, creatinine, albumin, CRP, ALAT, ASAT, GT, ALP, LD, bilirubin

3.4.5. Registration at time of death

a. The local study center or the Oslo University study center

The following will be collected and registered at time of death:

1. Hospitalizations since last follow-up (number of admissions, date(s), cause(s) in pre-defined categories (infection, pain, intracranial symptoms (headache, nausea/vomiting, cranial nerve related symptoms, reduced cognitive function), general deterioration related to cancer, other)
2. Steroid use and dosage
3. Date of last administration of systemic treatment
4. Salvage treatment(s) of intracranial disease if applicable [steroids (including dosage), surgery, radiotherapy (SRS, WBRT), best supportive care (including steroids only)]
5. Status of systemic disease at the time point closest to time of death (response, stable, progression)
6. Status of intracranial disease at the time point closest to time of death (response, stable, progression, local progression, distal intracranial progression)
7. Dedicated palliative specialists involved in patient care (Yes/no)
8. Radiology reports of the latest evaluation of systemic disease and of BM
9. Results of blood samples analyzed at the participating hospital's local clinical chemical laboratory at the time point closest to time of death
    a) Hemoglobin, platelets, leucocytes, creatinine, albumin, CRP, ALAT, ASAT, GT, ALP, LD, bilirubin
10. Time of death
11. Likely cause of death (extracranial, intracranial, other)
12. Place of death (home, hospital, palliative care unit, hospice/care center, other)

3.4.6 Long-term follow-up after 24 months or more after inclusion

A proportion of study participants live beyond the 24 month follow-up period. These patients will be invited to a long-term follow-up consisting of clinical examination, neuro-cognitive assessment, radiology and registration of patient-reported outcomes, described in the following. The majority of the study patients have received treatment that may have detrimental long-term effects on cognitive and physical functioning and activities of daily living. Studies investigating this are scarce. Thus, there is a need for better knowledge about the long-term development of disease and treatment effects. This will be examined by objective, clinical examinations coupled with patients' self-report of symptoms, problems and quality of life.

a. Invitation to participate

All patients eligible for this long-term follow-up (approximately 50) receive a letter of invitation by post. The letter explains the rationale for long-term follow-up, which is to improve our follow-up and care to patients who live for a long time with brain metastases. The content of the clinical and cognitive examinations will be described. All patients have previously consented to participate in the study. However, an additional information and consent form for this follow-up study is part of the letter of invitation. They consent to participate by returning a signed version of the extension of the patient information. If the patients have not responded within three weeks, an additional invitation will be mailed. The patients may choose to participate through telephone- or video consultation instead of a physical appointment at the hospital.

b. Clinical examination

Participants will be interviewed and examined by a physician according to standard clinical procedure for patient consultations, including neurological performance status. Additionally, a semi-structured interview guide emphasizing coping and challenges in activities of daily life will be included in the consultation.

c. Neuro-cognitive assessment

Neuro-cognitive assessment will be performed using a set of selected standardized neuro-cognitive tests. These tests cover important areas for cognition and everyday functioning such as short- and long-term memory and executive functions. Specialists in neuro-cognitive testing are included in the project group. Careful selection of appropriate tests will be done to reduce patient burden and the testing will be cancelled if the patient is not able to complete all parts of the tests.

d. Radiology

MRI and/or CT-scans of the brain are part of routine follow-up of patients with brain metastases and are performed both at OUS and local hospitals. Regular CT-scans for extracranial disease is also performed routinely. Results of these routine radiology procedures will be recorded (see also *g. Clinical follow-up data*). The value of 18F-fluordeoxyglucose

positron emission tomography (FDG PET) in follow-up of these patients regarding characterization of brain metastases, progression, pseudo-progression and/or radionecrosis is not established. Additionally, FDG-PET is proposed as a potential biomarker for cognitive functioning by assessing pathological metabolism in the brain. FDG-PET will therefore be performed and compared to results of neuro-cognitive assessments and PROMS, as well as the routine MRI/CT-scans of the brain.

e. Procedure if pathological findings on FDG-PET

If new pathologic lesions or intra- or extracranial progression are suspected from FDG-PET analysis, the treating oncologist at the patient’s local hospital will be informed for adequate follow-up of the patient.

f. Patient reported outcomes (PROMs)

Participants will be asked to complete the same paper questionnaires at this follow-up consultation and every 3 months thereafter, as used in the first 24 months of study (EQ-5D, EORTC-QLQ 15 PAL and BN-20). This provides a unique opportunity to compare objective evaluations of functioning, neurocognitive test results imaging results and patients’ own perceptions of problems and functioning. We will also ask the patients to complete these PROMS every three months after this follow-up consultation. The procedure for distribution and return of questionnaires will be by post (identical to the procedure previously used in the study).

g. Clinical follow-up data

Clinical data follow-up data identical to those previously collected from patient records (disease development, treatments, etc., see 3.4.4.b) will be registered in order to describe the disease trajectory between last follow-up in the study (at 24 months) and the time of the long-term follow-up. We will also ask the participants for permission to collect and register clinical follow-up data up to 24 months after the time of the long-term follow-up.

3.4.7. Flow chart for registrations

	Time of assessment after inclusion					
	Inclusion	1 month	2 months	Every month until 12 months	Every 3 months until 24 months	Time of death
Sociodemographic factors, oncologic history, BM characteristics	X					
Weight	X				X	
Performance status	X				X	
Current medication	X				X	
Current systemic cancer treatment (if any)	X				X	
Steroid use and dosage	X				X	X
Status of systemic disease (estimated clinically and/or radiologically)*	X				X	X
Status of BM (estimated clinically and/or radiologically)*	X				X	X
BM treatment	X				X	
Hospitalizations					X	X
Salvage treatment of BM*					X	X
Questionnaire on sociodemographic factors	X					
EORTC QLQ-C30	X	X	X	X	X	
EORTC QLQ-BN20	X	X	X	X	X	
EQ-5D	X	X	X	X	X	
Palliative specialists involved in patient care	X				X	X
Radiology reports of evaluation of systemic disease and BM	X				X	X
Results of blood samples	X				X	X
Place of care	X				X	
Date of last administration of systemic cancer treatment						X
Time of death						X
Cause of death						X
Place of death						X

**If clinically indicated and/or available*

3.5. Biobanking and analysis of tissue, including genetic analyses

If patients included in the study undergo surgery either as diagnostic procedure (biopsy) or treatment (resection) for BM, the study will collect tissue samples from these biopsies/resected tissue. These tissue samples will be stored in a biobank established for this purpose, together with blood samples for germ line analysis and available biopsy tissue from previous diagnostic and/or therapeutic procedures. In the study, high-throughput, targeted genetic analyses will be performed on tumor tissue DNA and germ-line DNA to explore differences in genetic sequences between germ-line DNA and tumor DNA from brain metastases, the primary tumor, and extracranial metastases. The general objective is to characterize the genotype of brain metastases, and may include molecular and functional studies of the metastatic process. As they are targeted, and the aim is finding differences between normal DNA and tumor DNA, the analyses are not considered predictive and the results of the analyses will not be routinely brought back to the patients. If, co-incidentally, findings should indicate conditions that may influence the patient's health, or the patient request information on the results, specific action are to be taken, as described below.

3.6. Procedure for informing the patient if genetic analyses reveal conditions that may influence the patient's health

Should the genetic analyses described above (see *3.5 Biobanking and analysis of tissue, including genetic analyses*) reveal conditions that may influence the patient's health, or should the patient request information on the results of such analyses, the following action will take place:

1. The CPI is informed of the results or the patient's request
2. The CPI contacts and discuss the results with the Department of Genetics at Oslo University Hospital
3. If the decision to inform the patient is made after discussion with the Department of Genetics, the CPI is responsible for contacting the treating physician in order to facilitate that the patient is contacted and informed of the results and offered genetic counselling
4. The patient is then referred to such genetic counselling by the informer

3.7. Procedure for issuing patient questionnaires

The patient questionnaires (see 3.4.3.b and 3.4.4.a) will be sent to patients by mail monthly for up to twelve months. This will be administered by the main study center in Oslo. If the questionnaires are not returned the patient will not be reminded, but the questionnaires for the following month will be sent as planned. If these questionnaires are also not returned, the administration of further questionnaires will be terminated.

4. Ethical considerations

4.1. Ethics approval

The study will be carried out in accordance with the World Medical Association Declaration of Helsinki and its revisions. Ethical approval will be sought from the Regional Committees of Medical and Health Research Ethics in each participating health region. Authorization for data access will be applied for to the Data Protection Agency according to institutional procedures and only authorized investigators will have access to study data.

4.2. Informed consent

All patients will be given oral and written information about the study and a written consent must be collected before inclusion in the study. This will include consent for collection of clinical information, collection of samples for biobanking (see above), linkage between the study registry and other population based registries (see also *3.4 Data collection*), and the possibility for exchange of data to participating centers, including abroad, according to institutional regulations and procedures. The consent must be obtained by the investigator including the patient in the study. All participants are free to withdraw from the study at any given time without giving any reasons or prejudicing further treatment or clinical follow-up. Completed patient consent forms must be retained at each site and properly filed. All patients must receive a copy of the signed patient consent form for their own records. Consent will be sought after proper information and time for consideration.

Should any potential participant be identified through data from the Norwegian Patient Registry or any other source, and/or inclusion with written informed consent for any reason cannot be

done, the participant may be registered anonymously with primary diagnosis and date of BM only to increase data completeness for estimation of incidence.

If any patient does not wish to participate the study center must ensure that the patient will not be confronted by repeated requests of participation by recording that the patient has been asked for participation.

4.3. Treatment of patients

The study will not involve interventions with novel or experimental treatment procedures. The patients will be treated in accordance with current practices and guidelines and the patient's and physician's preferences (see also *3.4. Risk*).

4.4. Risk

This study includes no interventions that will increase the risk of the patient's health or well-being. Treatment options are chosen according to current guidelines and at the discretion of the attending physician and patient preferences. Study related procedures are considered safe. Blood samples for study purposes will be performed together with routine blood samples. No biopsies are needed for participation in the study and will only be performed during surgical procedures that are clinically indicated, *i.e.* surgical biopsies or surgical resection for diagnostic purposes or treatment of the metastases.

A proportion of patients will suffer from decline in physical and cognitive function as a consequence of BM and/or cancer progression. As a result, patients may find answering the repeated question forms cumbersome and not have the energy or feel able to answer them. This will not exclude patients from participating in the study. If a patient is unable to answer the distributed question forms, this will be entered as missing data and will not be considered a break of study protocol or reason for exclusion from the study. This is also emphasized in the patient information.

A consequence of the procedure for issuing patient questionnaires (see *3.7. Procedure for issuing patient questionnaires*) can be that questionnaires are sent to the patients after a time point when they feel unable to answer them or that they have died. The written information that

is given to the patient at inclusion and attached to every questionnaire contains contact information to the Chief Principal Investigator so that the CPI may be contacted in such cases.

4.5. Data collection, data storage, data exchange, data linking, and patient confidentiality

Case report forms (CRFs) will be developed and made available by the main sponsor center on paper and electronically. The study will collect data electronically through a web-based tool (web-CRF) developed by NTNU, Trondheim and currently used in many clinical studies. This tool allows secure transmission and storage of data through encryption and data output files do not contain the national social security number. Access to the encrypted data file will only be given to the CPI and the study nurse at the Oslo University Hospital study center.

If any study center wishes to enter data on paper CRFs rather than the TSD platform, the CRFs will be made available by the Oslo University Hospital study center. After registration on paper CRFs, the CRFs will then be sent by mail to the main study center where the data will be entered manually into the data file. The paper CRFs will then be stored in a locked archive for later data checks purposes.

Health data will be collected and stored securely in accordance with institutional, national and international ethical standards. Current institutional guidelines for storage, transmission, and disclosure of patient information will be followed at all times. Study records and files for each patient will be maintained with a unique study ID-number to identify patients in the study data files. A code list ("koblingsnøkkel") will link personal data with the corresponding study number. The principal investigator at each site is responsible for keeping a code list of all included patients at the site. The code list contains full name, address, phone number, and social security number for the included patients. See also *3.4 Data recording.*

Additional data will be collected from The National Patient Registry (NPR), the Norwegian Cancer Registry, and the Norwegian Cause of Death Registry ("Dødsårsaksregisteret"). This will be done by linking data from the study with the relevant registry, according to standard procedures ensuring confidentiality. Relevant authorizations and accesses will be applied for and the utility of such registries is included in the ethics approval and the patient information and consent forms. See also *3.4.1 Linking of data from other registries or sources.*

Data from the study may also be exchanged with collaborating centers in Norway and abroad, both inside and outside of EU. Such data exchange will be made according to current

institutional guidelines at Oslo University Hospital and data will be un-identifiable, using study ID-number only without the corresponding code lists. Information on such data exchange is included in the ethics approval and the patient information and consent forms.

Representatives from the Study sponsor and regulatory authorities will be given access to the records that relate to the study, with full access to all data as required.

The data file containing all study data will be deleted 15 years after end of inclusion (2037), giving access to data for a total of 20 years. Deletion of data will be performed according to guidelines at each institution.

Results of the study may be communicated at scientific meetings and published in internationally peer-reviewed journals. The identification of any individual patient will not be made possible at any times.

4.6 Liability and insurance

The patients are insured according to the Patient Injury Act (Pasientskadeloven).

5. Study management

Allocation of study responsibilities

The main sponsor of this study is Stein Kaasa, Oslo University Hospital and NTNU. The sponsor’s responsibilities will be the overall administration and conduct of the study.

Principal investigators

A Chief Principle Investigator (CPI), Olav Erich Yri, is responsible for the management of the study. Co-principal investigator is Stein Kaasa. The main study centers will be Oslo University Hospital in Oslo and St Olav in Trondheim. The CPI is based at Oslo University Hospital. The main activities related to the development and conduct of the study is the responsibility of the CPI through the main study centers. Each participating local study center must have a local Principle Investigator (PI) that will serve as the link between the local study center and the main study centers and is responsible for ensuring protocol adherence and the availability of protocol and its amendments to local site investigators.

The PI at each participating study center is responsible for:

- Obtaining the appropriate local research regulatory board recommendations for the study
- Adherence to the study protocol and local regulatory procedures for study participation.
- The acts and omissions of its own staff and others engaged by it
- Ensuring the appropriate insurance is in place
- Ensuring that any employees involved in the study have contracts to cover access to patients and liability arrangements.
- Permitting trial related monitoring, audits, ethics review, and regulatory inspections as required by providing direct access to data and other relevant documents (patient records, study site files, et cetera).

Study Management group

A management group will be formed consisting of the CPI, the co-principal investigator, the PI at St. Olav Hospital study center, co-workers at Department of Oncology, Oslo University Hospital, and the study nurse(s) at both main study centers, and study statistician. The management group will be responsible for study administration, including development and administration of CRFs, monitoring of progress and data quality, feedback to PIs, administration of question forms, and preparation of the final study report. All study administration will be the responsibility of the main study centers (Oslo University Hospital and St. Olav, see above).

Study Steering group

The management group and the principal investigators at each participating site will form a Study steering group. The user representative will also be included in the group. The Study steering group will serve as a forum to discuss progress and challenges of the study and suggest adjustments to the protocol. The group may meet at regular intervals or by telecom conferences etc. to discuss such issues.

6. Quality assurance

Quality assurance will be accomplished by the following:

- The principal investigators (PIs) of the study sites and their study staff must ensure that the study is conducted in compliance with the protocol, good clinical practice (GCP) and relevant regulatory requirements
- The Study management group will assist the investigators at each site and assure compliance and adherence to the study protocol, GCP, and regulatory requirements by monitoring study documentation at monitoring visits. The Study management group will have the all-over responsibility for study compliance
- Central monitoring of study data completeness and accuracy will be performed by the Study management group
- The Study management group will control data consistency and data quality by reviewing the study database. Computerized and manual consistency checks will be performed and queries issued if inconsistency or missing information is revealed. A full audit trail of any changes to the database will be maintained.
- The Study Steering Group will ensure that the study is managed appropriately by the Study management group.

7. Definition of end of inclusion and end of study

The end of inclusion of the study is set at 31st of December, 2022. End of study for the individual patient is time from inclusion until death of the patient or 2 years after end of inclusion, whichever comes first.

The data file containing all study data will be deleted 15 years after end of inclusion (2037), giving access to data for a total of 20 years. Deletion of data will be performed according to guidelines at each institution.

8. Finances and conflict of interest

Financial support is granted from Helse Sør-Øst and Rosa Sløyfe for a period of 5 years. No commercial interests are involved in the study. No conflicts of interest have been identified. Patients receive no financial benefits for participation.

9. Publication and dissemination

A website related to the study will be established to provide general information about the study. The patients (via the user representative) and relevant patient-organizations will be included in dissemination activities. The results from the study will be presented at relevant national and international meetings and submitted to international peer-reviewed medical journals. Authorship will be defined according to the Vancouver Rules. All PIs at each participating center will be co-authors on the main publication from the study. Authorship on subsequent papers will be decided by the Study steering group.

10. References

1. Cancer in Norway 2015 - Cancer incidence, mortality, survival and prevalence in Norway. Cancer Registry of Norway 2015.
2. Fox BD, Cheung VJ, Patel AJ, Suki D, Rao G. Epidemiology of metastatic brain tumors. Neurosurgery clinics of North America. 2011;22(1):1-6, v.
3. Smedby KE, Brandt L, Backlund ML, Blomqvist P. Brain metastases admissions in Sweden between 1987 and 2006. British journal of cancer. 2009;101(11):1919-24.
4. Naehrig DN, Koh ES, Vogiatzis M, Yanagisawa W, Kwong C, Shepherd HL, et al. Impact of cognitive function on communication in patients with primary or secondary brain tumours. Journal of neuro-oncology. 2016;126(2):299-307.
5. Soffietti R, Abacioglu U, Baumert B, Combs SE, Kinhult S, Kros JM, et al. Diagnosis and treatment of brain metastases from solid tumors: guidelines from the European Association of Neuro-Oncology (EANO). Neuro-oncology. 2017;19(2):162-74.
6. Tsao MN, Rades D, Wirth A, Lo SS, Danielson BL, Gaspar LE, et al. Radiotherapeutic and surgical management for newly diagnosed brain metastasis(es): An American Society for Radiation Oncology evidence-based guideline. Practical radiation oncology. 2012;2(3):210-25.
7. Tsao MN, Rades D, Wirth A, Lo SS, Danielson BL, Vichare A, et al. International practice survey on the management of brain metastases: Third International Consensus Workshop on Palliative Radiotherapy and Symptom Control. Clinical oncology. 2012;24(6):e81-92.
8. Tsao MN, Lloyd N, Wong RK, Chow E, Rakovitch E, Laperriere N, et al. Whole brain radiotherapy for the treatment of newly diagnosed multiple brain metastases. The Cochrane database of systematic reviews. 2012(4):CD003869.
9. Mulvenna P, Nankivell M, Barton R, Faivre-Finn C, Wilson P, McColl E, et al. Dexamethasone and supportive care with or without whole brain radiotherapy in treating patients with non-small cell lung cancer with brain metastases unsuitable for resection or stereotactic radiotherapy (QUARTZ): results from a phase 3, non-inferiority, randomised trial. Lancet. 2016;388(10055):2004-14.
10. Shibamoto Y, Baba F, Oda K, Hayashi S, Kokubo M, Ishihara S, et al. Incidence of brain atrophy and decline in mini-mental state examination score after whole-brain radiotherapy in patients with brain metastases: a prospective study. International journal of radiation oncology, biology, physics. 2008;72(4):1168-73.
11. Brown PD, Jaeckle K, Ballman KV, Farace E, Cerhan JH, Anderson SK, et al. Effect of Radiosurgery Alone vs Radiosurgery With Whole Brain Radiation Therapy on Cognitive Function in Patients With 1 to 3 Brain Metastases: A Randomized Clinical Trial. Jama. 2016;316(4):401-9.
12. Chang EL, Wefel JS, Hess KR, Allen PK, Lang FF, Kornguth DG, et al. Neurocognition in patients with brain metastases treated with radiosurgery or radiosurgery plus whole-brain irradiation: a randomised controlled trial. The Lancet Oncology. 2009;10(11):1037-44.
13. Soffietti R, Kocher M, Abacioglu UM, Villa S, Fauchon F, Baumert BG, et al. A European Organisation for Research and Treatment of Cancer phase III trial of adjuvant whole-brain radiotherapy versus observation in patients with one to three brain metastases from solid tumors after surgical resection or radiosurgery: quality-of-life results. Journal of clinical oncology : official journal of the American Society of Clinical Oncology. 2013;31(1):65-72.

14. Hart MG, Grant R, Walker M, Dickinson H. Surgical resection and whole brain radiation therapy versus whole brain radiation therapy alone for single brain metastases. The Cochrane database of systematic reviews. 2005(1):CD003292.
15. Patchell RA, Tibbs PA, Regine WF, Dempsey RJ, Mohiuddin M, Kryscio RJ, et al. Postoperative radiotherapy in the treatment of single metastases to the brain: a randomized trial. Jama. 1998;280(17):1485-9.
16. Sahgal A, Aoyama H, Kocher M, Neupane B, Collette S, Tago M, et al. Phase 3 trials of stereotactic radiosurgery with or without whole-brain radiation therapy for 1 to 4 brain metastases: individual patient data meta-analysis. International journal of radiation oncology, biology, physics. 2015;91(4):710-7.
17. Tsao M, Xu W, Sahgal A. A meta-analysis evaluating stereotactic radiosurgery, whole-brain radiotherapy, or both for patients presenting with a limited number of brain metastases. Cancer. 2012;118(9):2486-93.
18. Yamamoto M, Serizawa T, Shuto T, Akabane A, Higuchi Y, Kawagishi J, et al. Stereotactic radiosurgery for patients with multiple brain metastases (JLGK0901): a multi-institutional prospective observational study. The Lancet Oncology. 2014;15(4):387-95.
19. Maqbool T, Agarwal A, Sium A, Trang A, Chung C, Papadakos J. Informational and Supportive Care Needs of Brain Metastases Patients and Caregivers: a Systematic Review. Journal of cancer education : the official journal of the American Association for Cancer Education. 2016.
20. ASTRO. Choosing Wisely. ASTRO releases second list of 5 radiation oncology treatments to question as part of national Choosing Wisely campaign. . http://wwwchoosingwiselyorg/astro-releases-second-list/. 2014.
21. Fogarty GB, Hong A, Gondi V, Burmeister B, Jacobsen K, Lo S, et al. Debate: adjuvant whole brain radiotherapy or not? More data is the wiser choice. BMC cancer. 2016;16:372.
22. Li J, Brown PD. The Diminishing Role of Whole-Brain Radiation Therapy in the Treatment of Brain Metastases. JAMA oncology. 2017.
23. D'Souza NM, Fang P, Logan J, Yang J, Jiang W, Li J. Combining Radiation Therapy with Immune Checkpoint Blockade for Central Nervous System Malignancies. Frontiers in oncology. 2016;6:212.
24. Rodrigues G, Bauman G, Palma D, Louie AV, Mocanu J, Senan S, et al. Systematic review of brain metastases prognostic indices. Practical radiation oncology. 2013;3(2):101-6.
